# Collective Strong Coupling Modifies Aggregation and Solvation

**DOI:** 10.1021/acs.jpclett.3c03506

**Published:** 2024-01-30

**Authors:** Matteo Castagnola, Tor S. Haugland, Enrico Ronca, Henrik Koch, Christian Schäfer

**Affiliations:** † Department of Chemistry, Norwegian University of Science and Technology, 7491 Trondheim, Norway; ‡ Dipartimento di Chimica, Biologia e Biotecnologie, Universitá degli Studi di Perugia, Via Elce di Sotto 8, 06123 Perugia, Italy; § Scuola Normale Superiore, Piazza dei Cavalieri 7, 56126 Pisa, Italy; ∥ Department of Physics, Chalmers University of Technology, 412 96 Göteborg, Sweden; ⊥ Department of Microtechnology and Nanoscience (MC2), Chalmers University of Technology, 412 96 Göteborg, Sweden

## Abstract

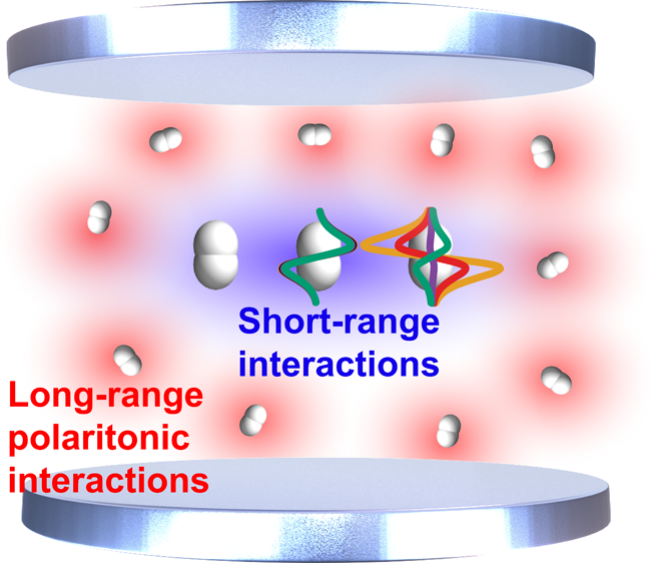

Intermolecular (Coulombic) interactions are pivotal for aggregation, solvation, and crystallization. We demonstrate that the collective strong coupling of several molecules to a single optical mode results in notable changes in the molecular excitations around a single perturbed molecule, thus representing an impurity in an otherwise ordered system. A competition between short-range coulombic and long-range photonic correlations inverts the local transition density in a polaritonic state, suggesting notable changes in the polarizability of the solvation shell. Our results provide an alternative perspective on recent work in polaritonic chemistry and pave the way for the rigorous treatment of cooperative effects in aggregation, solvation, and crystallization.

The coherent interaction of molecules with confined optical modes leads to hybrid light–matter states called polaritons.^1−3^ In the strong coupling regime, usually achieved by coupling several molecules to the optical cavity, experiments show significant modifications of chemical properties.^4−19^ As a result of their delocalized nature, polaritons in quantum optics are studied from a collective perspective where the molecules are modeled as few-level systems that indirectly interact solely through the photon field.^20−26^ However, chemistry is governed by local interactions, and the molecular complexity requires refined chemical methods to analyze processes like reactions. The behavior of molecules is, hence, susceptible to their immediate surroundings. For example, their spectral absorption and emission can be altered by their interplay with the solvent (solvatochromic) or by the close proximity to identical molecules (concentration-dependent). Experiments under strong coupling often use organic molecules that are sensitive to their surroundings, show intense excitations, or even form aggregates.^6−9,27−32^ The chemical environment can then play an active role and exert significant influence, as emphasized by several vibrational strong coupling (VSC) experiments showing modification of assembly^33−35^ and reactivity.^14,36,37^ For a clear chemical understanding of polaritonic processes, we must then investigate the coexisting roles of the molecule, the solvent (chemical environment), and the cavity (optical environment). To this end, *ab initio* quantum electrodynamics (QED) merges the knowledge of quantum optics and quantum chemistry to explore single-molecule effects retaining the chemical complexity.^38−50^ Even if collective states can show larger contributions from selected molecules,^45,51^ local changes tend to reduce as a result of collective delocalization and an increasing number of quasi-dark states.^52^ While recent work indicates a larger relevance of dynamic electronic polarization,^50,53,54^ it remains puzzling how the collective nature of polaritons enters chemical processes. The role of changes in the chemical environment (solvents and aggregates) under strong coupling has been widely disregarded up to this point.

In this letter, we add another facet to the understanding of polaritonic chemistry by reintroducing the chemical environment. Using QED coupled cluster (QED-CC),^46^ which is at present the most accurate approach for medium-sized molecular polaritons, we study the yet unexplored competition between photon and intermolecular interactions. We extracted the single-molecule response in collective ensembles and investigated the microscopic changes of aggregates or solvation systems in polaritonic chemistry. We demonstrate local response modifications unambiguously distinguishable from collective delocalization arising from the interplay between Coulomb and transverse fields. Moreover, our results show a slower decrease than conventional local effects in polaritonics, i.e., not with the total number of coupled molecules but with the number of affected solvation shells. As a result, the immediate surroundings of the impurity, representing a solute, nucleation, or reaction center, undergo notable changes. Structural changes in the chemical environment can then influence the dynamics of the impurity. Our simple model, a stretched molecule embedded in a perfectly ordered environment, can represent a solute in a solvent, a nucleation center, or an impurity in an aggregate. Indeed, these physical realizations are associated with intermolecular (Coulombic) interactions with the surrounding chemical environment. Therefore, solvation, aggregation, and nucleation are used interchangeably in the following as a result of their conceptual similarity.

*Modeling Solvation and Collective Coupling*. Molecular properties are significantly modified by chemical environments, such as polymer matrices, solvents, or aggregates. The interaction of a solute with its surrounding molecules leads to changes in the ground and excited electronic densities, which affect energy levels, molecular geometries, and even chemical reactivity. In addition, the solvent also directly contributes to properties, such as spectroscopic signals. To study the impact of polaritons on the complex chemical response, we must simultaneously include photon coupling and Coulomb interactions in the solute–solvent system. Therefore, we focus on a system with non-negligible intermolecular interactions as a prototype for Coulomb forces in supramolecular structures. Our model system is a chain of *N* hydrogen molecules that form a H-aggregate illustrated in Figure 1. An impurity (P), representing a solute or reactive molecule, is introduced by stretching the bond of the central hydrogen, and we investigate different intermolecular distances to alter the Coulomb forces. Figure 1 shows the absorption spectrum of the undressed (out-of-cavity) chain for different *N* and 5 Å intermolecular separation. The global response of the system is governed by the transition density ϱ_*j*0_(*x*, *y*, *z*), which is, for instance, connected to the optical properties via the transition dipole moment . To characterize the local behavior of each dimer, we integrate ϱ_*j*0_(*x*, *y*, *z*) perpendicularly to the excitation transition dipole moments (that is, in the *y* and *z* directions) around P, its nearest neighbor A1 (first solvation shell), and its next nearest neighbor A2. The integrated local transition densities ρ_*j*0_^*I*^(*x*) = ∫_–∞_^+∞^ d*z* ∫_*Y*_*I*_^(1)^_^*Y*_*I*_^(2)^^ d*y* ϱ_*j*0_(*x*, *y*, *z*) (*I* = P, A1, and A2) for the excited states E1 and E2 are depicted in Figure 1. The excitation E1 is mainly localized on P and A1 with anti-aligned local transition dipoles, while E2 is delocalized and shows an all-parallel transition moment alignment. In Figure 2, we show the polaritonic absorption spectrum with the photon resonant to the (isolated) unperturbed H_2_ excitation with light–matter coupling strength  = 0.005 au, corresponding to an effective mode volume *V* = 74.5 nm^3^, which is achievable with, e.g., plasmonic resonators. We will demonstrate in the following that the fundamental coupling strength is nonetheless of secondary relevance. Three polaritonic branches, the lower polariton (LP), middle polariton (MP), and upper polariton (UP) emerge. The electron and electron–photon correlation are accurately described by the *ab initio* QED-CC ansatz wave function, thus capturing any modification of inter- (dispersion) and intramolecular forces.^37,46,47,55,56^ Nevertheless, as a result of the low coupling strength, the molecular ground state, here, is essentially unaffected by the embedding in the optical environment and polaritonic effects effectively arise from collective coupling. The dimers are indirectly coupled through the cavity, establishing an interplay between longitudinal (Coulomb) and transverse (photon) fields. The simplicity and engineerability of our model allow us to separate intermolecular and cavity-induced effects, providing a satisfactory proof-of-concept model for investigating microscopic molecular responses in complex chemical and photonic environments.

**Figure 1:**
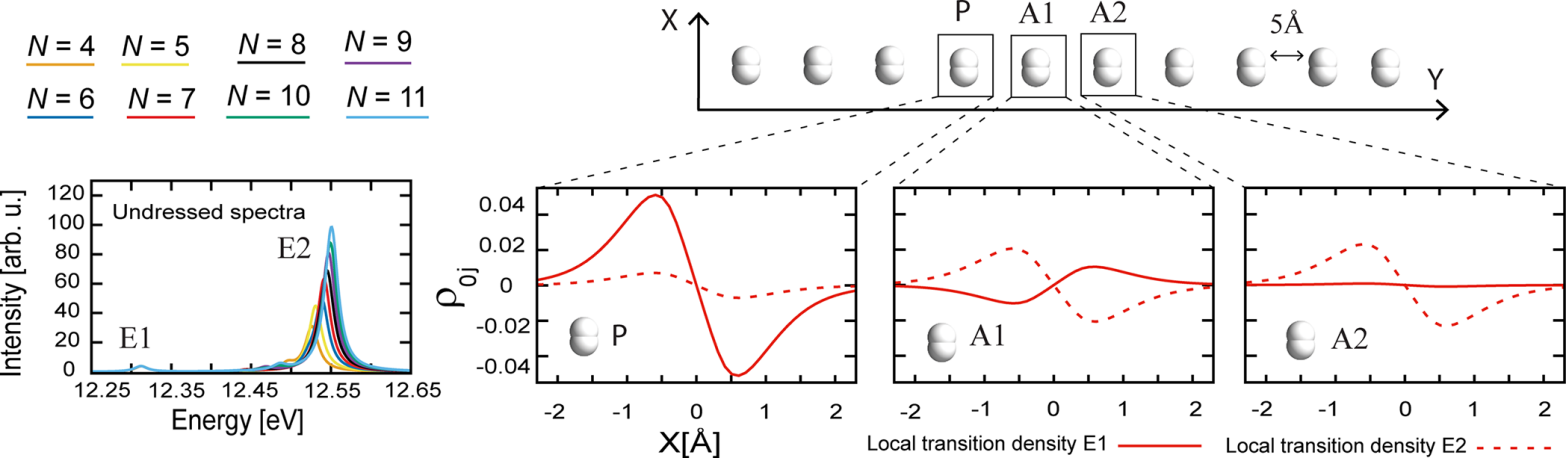
Structure of the (H_2_)_*N*_ aggregate and absorption spectra outside the cavity for 5 Å intermolecular separation. The local transition densities ρ_*j*0_(*x*) [i.e., the transition density ϱ_*j*0_(*x*, *y*, *z*) integrated perpendicularly to the dipole moment direction, that is, perpendicular to the H_2_ bond ρ_*j*0_^*I*^(*x*) = ∫_–∞_^+∞^ d *z* ∫_*Y*_*I*_–2 Å_^*Y*_*I*_+2 Å^ d*y* ϱ_*j*0_(*x*, *y*, *z*), where *I* = P, A1, and A2; see section 1 of the Supporting Information] of the undressed excitations E1 (solid lines) and E2 (dotted lines) of (H_2_)_7_ are shown, showing specific transition moment alignment patterns caused by intermolecular interactions. Only the left densities are shown because the right densities display the same behavior (see section 1 of the Supporting Information).

**Figure 2:**
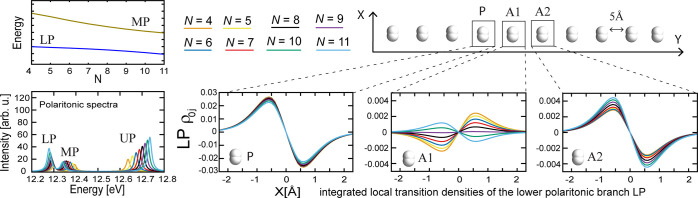
Polaritonic spectra for 5 Å intermolecular separation with coupling strength 0.005 au, polarization along the H_2_ bonds, and photon frequency tuned to the undressed excitation of the isolated unperturbed dimers. The energies of the lower and middle polaritons as a function of the number of dimers *N* in the chain, depicted in the top left panel, highlight an avoided crossing. The integrated local transition density of the lower polariton (LP) for P, A1, and A2, are shown. The Coulomb forces compete with the transverse field (photon) to determine the alignment pattern of the transition moments, generating a sign flip in the transition density of A1.

*Modification of Solvation*. In Figure 2, we plot the LP local transition densities for P, A1, and A2. As *N* increases, the A1 local transition density changes sign; i.e., the transition dipole of A1 aligns with the other molecules. This effect requires that both of the (undressed) excitations E1 and E2 contribute to the LP state and, hence, occur around the avoided crossing domain illustrated in Figure 2. While the alignment clearly originates from collective effects, its impact is evidently localized in the first solvation shell and requires explicit treatment of the solute–solvent interface. The local change arises from competition between the short-range intermolecular forces, which pattern E1 and E2, and the collective interaction to the optical mode, which tends to align the molecular transition moments (see also section 3 of the Supporting Information). Indeed, for larger intermolecular separations, the LP local moments are aligned (see section 5 of the Supporting Information), confirming that the sign change stems from the interplay between Coulomb and photon fields. Which effect prevails depends upon the setup, specifically the excitation energies (solute–solvent system), the chain length *N* (collective coupling), the light–matter coupling strength  (optical device features), and the intermolecular forces (solute–solvent system and intermolecular separation). Notice also that there is a collective strength for which the excitation of the nearest dimers is effectively quenched by collective strong coupling (*N* = 9 in Figure 2). Analogous quenching effects play an important role in limiting the refractive index of atomic media.^57^

The number of solvent molecules heavily outweighs the concentration of solutes. It is therefore instructive to verify how our observation scales in the thermodynamic limit *N*_e_, *V* → ∞, *N*_e_/*V* = constant, i.e., with fixed *N*_e_/*V* but increasing particle number *N*_e_ and quantization volume *V* (controlling the fundamental coupling strength). To this end, we resort to a simplified Tavis–Cummings (TC)–Kasha^58−61^ model (parametrized according to the electronic CC calculations), with a nearest neighbor transition dipole–dipole coupling between the molecular excitations (see sections 3.3 and 5.1 of the Supporting Information). The total number of molecules *N*_e_ = *N*_rep_*N* is given by the number of molecules *N* per aggregate (i.e., the same number used in Figures 1 and 2) and how many times the system appears in the cavity *N*_rep_. In Figure 3a, we report the eigenvector coefficients for single P and A1 to quantify how the individual molecules contribute to the collective excitations of the TC–Kasha model. The ratio *N*_e_/*V* is fixed; i.e., the Rabi splitting is kept constant while increasing *N*. The simulations for *N*_rep_ = 1 and 4 show analogous results and, thus, prove that the described behavior is qualitatively resistant to the thermodynamic limit. Still, the single-molecule contribution (i.e., the TC–Kasha eigenvector coefficients of a single dimer) decreases by approximately . Similar conclusions are drawn from panels b and c of Figure 3, where, for each panel, λ and *N*_rep_ are fixed while increasing *N*. This allows us to investigate the effect of changing the number of coupled molecules and, thus, the collective effects. Increasing the number *N* – 1 of coupled solvent molecules at fixed *N*_rep_ and λ shortens the critical intermolecular distance at which the A1 coefficient shows a change. Panels b1 and b2 of Figure 3, computed at different couplings, show similar behavior and order of magnitude, but the *N*-value for which the transition moment of A1 changes sign is shifted to longer chains for smaller couplings. Comparing panels b1 and c of Figure 3 shows the effect of increasing *N*_rep_/*N* while rescaling λ by , which thus retains the overall collective coupling. The panels show the same qualitative behavior, with coefficients rescaled by , as in Figure 3a. We also confirm this trend by simulating two H_2_ chains in our QED-CC calculations (see section 5.1 of the Supporting Information). Therefore, Figure 3 demonstrates that the magnitude of the change in the environmental response for fixed *N*_e_/*V* decreases with the number of activated species P, which is significantly smaller than the number of coupled molecules *N*_e_, and P does not need to be strongly coupled to the device. In other words, if the solvent exists in vast excess as in experiments showing modifications of crystallization^33−35^ and ionic conductivity,^62^ each solvation shell will experience a considerably larger effect from the cavity than the bulk of the solvent molecules. Moreover, keeping the solute–solvent ratio fixed (increasing *N*_rep_) shortens the critical length *N* as seen from panels b and c of Figure 3. Therefore, the intermolecular distance and chain length *N* for which the transition moment of A1 changes sign depend solely upon the solvent strong coupling defined by the ratio *N*_rep_(*N* – 1)/*V*.

**Figure 3:**
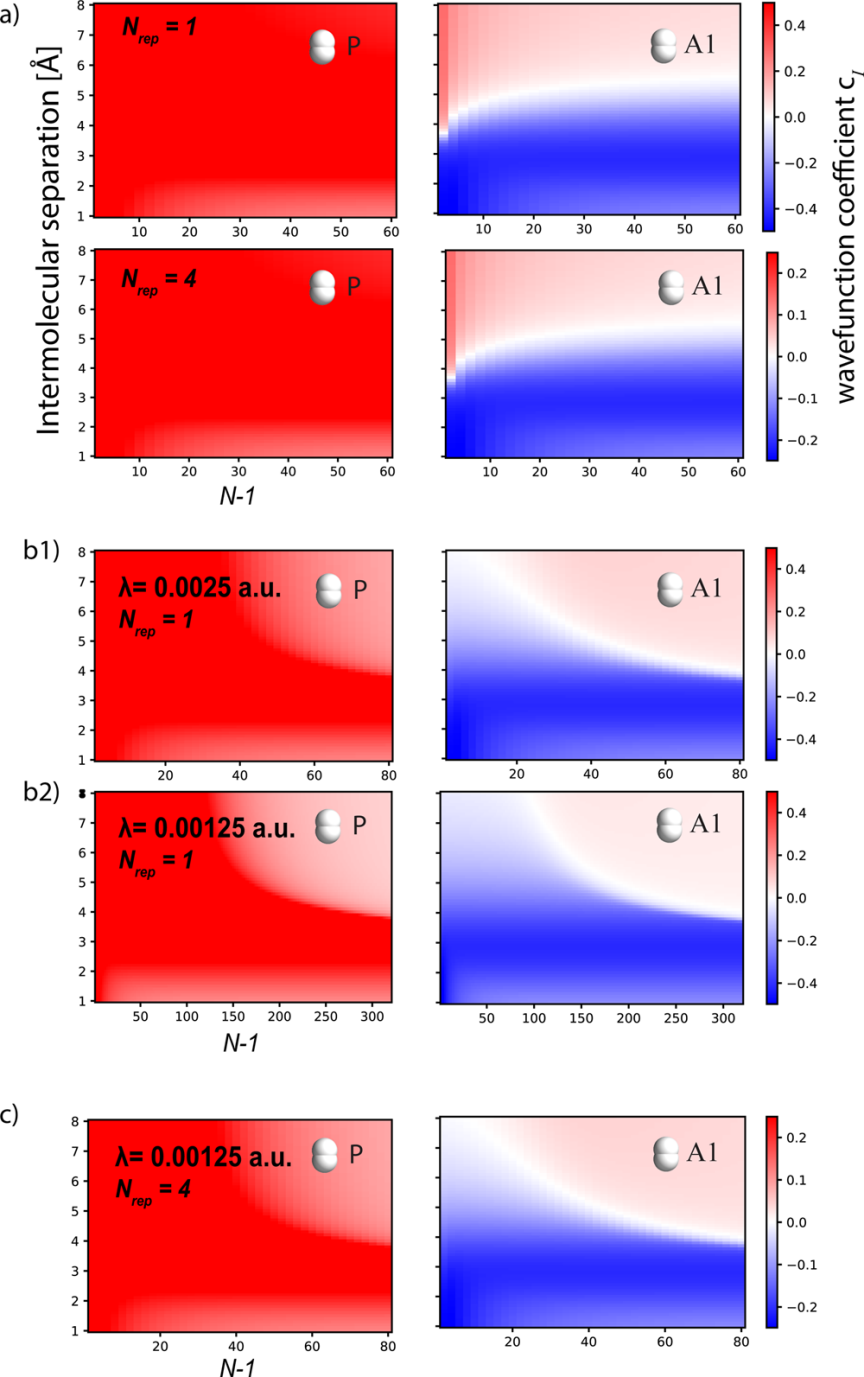
Wave function coefficients *c*_*I*_, where *I* = P and A1 from the TC–Kasha model as a function of the environment molecules *N* – 1 and the intermolecular separation. (a) The coupling strength is set to . We qualitatively recover the same sign flip for A1 as Figure 2 for *N*_rep_ = 1 (upper panel) and 4 (lower panel), but the coefficient values approximately scale as . Notably, such a decay is much smaller than . (b) Wave function coefficients obtained by fixing λ and *N*_rep_ = 1 as *N* varies. This is consistent with experimental setups where the same cavity (i.e., λ fixed) is employed at different molecular concentrations (here, the solvent molecules *N* – 1). (c) Same as panel b but for *N*_rep_ = 4. In comparison to panel b1, the overall collective coupling is maintained, but as a result of the larger number of solute molecules, the coefficients are rescaled by . The impurity concentration P then defines the decay of the wave function coefficients. Notice that P does not need to be in a strong coupling. See section 5.1 of the Supporting Information for more results.

Thus far, we considered all of the molecules at the same distance. However, analogous results are found for a small H_2_ cluster, with the other hydrogens at larger distances. This is illustrated in Figure 4 for (H_2_)_3_ with intermolecular distance 3.7 Å and slightly increased λ = 0.01 au to compensate for the larger blue shift of E1. The remaining *N* – 3 hydrogens are placed at a 20 Å distance. The results show the same behavior as in Figure 2 and emphasize the locality induced by the short-range Coulomb interactions, clearly highlighting the interplay between the chemical surroundings and the collective optical dressing.

**Figure 4:**
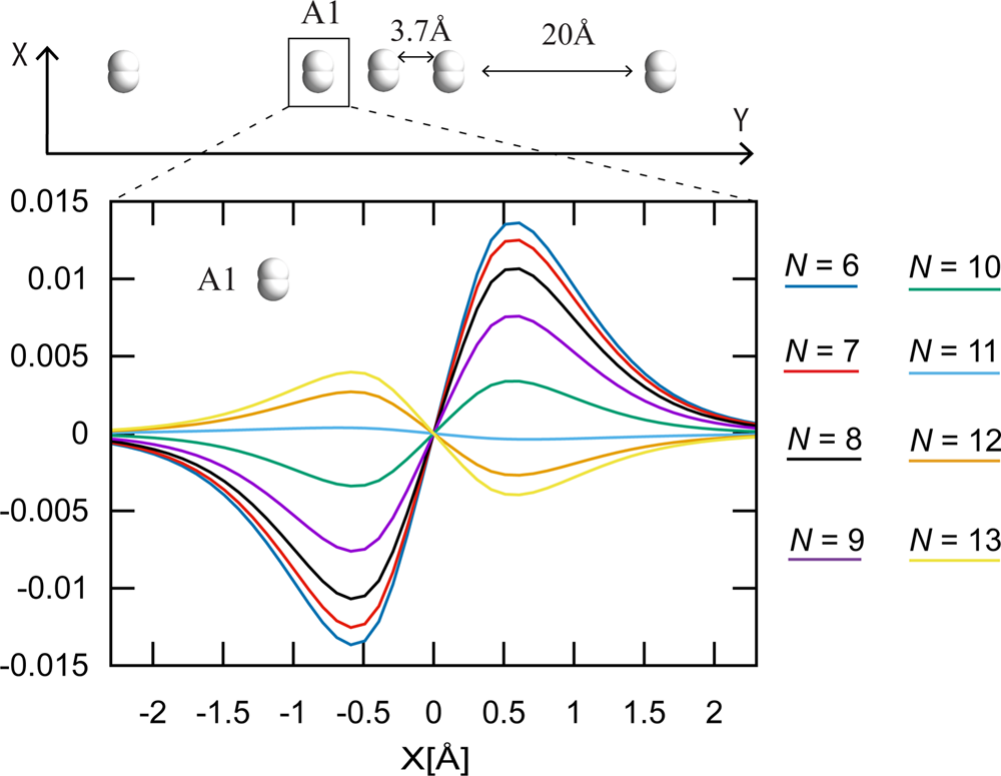
LP-integrated local transition densities for A1 for a cluster (H_2_)_3_ with intermolecular separation of 3.7 Å, together with *N* – 3 intermolecular dimers at a distance of 20 Å. As a result of the larger intermolecular forces between A1 and P, the excitation E1 is strongly blue-shifted. Therefore, we use a slightly larger coupling and a larger number *N* compared to Figure 2 to reach the avoided crossing region between the LP and MP. The results show the same qualitative behavior as Figure 2.

The TC–Kasha model successfully reproduces the modifications in the dynamic response of the first solvation shell. Because the model is parametrized with bare (electronic) transitions, it stresses the importance of collective behavior, which alters the LP microscopic response near P once a critical cooperative coupling strength is reached. Nevertheless, the TC–Kasha model has apparent limitations in that it will perform poorly in modeling the electronic dynamic of more complex systems and cannot account for nonlinear effects included in the QED-CC calculations; i.e., a self-consistent and more realistic treatment is likely to give similar or more favorable scaling. It should also be noted that the strength and structure of solvation vary widely with the type of intermolecular interaction such that the onset of this effect can change dramatically with the specific choice of solvent and solute. Quantum chemistry then provides a flexible tool set to explore such solvent effects beyond the heavily simplified dipole–dipole picture.^63−65^

*Disorder and Resonance Dependence*. While aggregation, solvation, and crystallization imply a somewhat structured environment, disorder (in the form of orientation or inhomogeneous broadening) is inevitable in chemistry under standard ambient conditions. Inhomogeneous broadening and the molecular orientation relative to the cavity polarization, for all solvent molecules besides A1 (long-range disorder), are included in our TC–Kasha model by sampling the excitation energies from a Gaussian distribution and assigning random H_2_ orientations (see section 5.1 of the Supporting Information for details). As demonstrated in Figure 5 and section 5.1 of the Supporting Information, the long-range disorder merely increases the required number of molecules *N*, but the overall solute–solvent effect remains unaffected. At the same time, local disorder severely impacts the intermolecular interactions because the Coulomb forces are strongly directional; i.e., other forms of aggregation will impact the solute differently. In the Supporting Information, we report the local transition densities for a J-aggregate (head–tail) (H_2_)_*N*_ configuration and show a sign flip for the local transition dipole of A1 in the MP instead of the LP. The modification of the solvent–solute response is conceptually identical and merely moves to a different polaritonic state. This stems from the different transition moment patterns in the undressed excitations of the J-aggregate. Hence, we can expect that a realistic solvation system will partially exhibit the discussed effect in the LP and MP.

**Figure 5:**
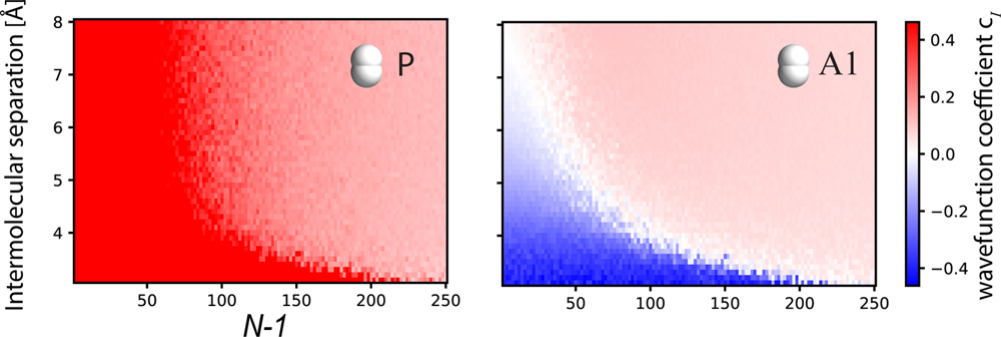
Wave function coefficients obtained from a disordered TC–Kasha model, where the first and second solvation shells around P are fixed, while orientational disorder and inhomogeneous broadening are applied to the other dimers. The coupling strength is set to 0.005 au. We still recover the same qualitative behavior and coefficient magnitude of Figures 2 and 3b although for larger *N* because of the smaller collective coupling to the device as a result of disorder. Analogous results are obtained for different coupling strengths; see section 5.1 of the Supporting Information for more results.

Lastly, the solute–solvent polarization features a clear resonant behavior. When the cavity is detuned from the E2 excitation (entirely off-resonant or tuned to E1), the local changes are negligible or indistinguishable from collective delocalization effects. If the field is tuned to E2 (instead of the excitation of isolated unperturbed H_2_), the results are in line with the foregoing discussion, although this requires a slightly higher *N* to achieve the sign flip in A1. These results are consistent with the position of the avoided crossing between the LP and MP, which depends upon the photon frequency and determines the mixing of E1 and E2 (and, thus, the local impact of the strong coupling regime).

*Conclusion*. Using state-of-the-art *ab initio* QED-CC and quantum optics models, we illustrate how the intermolecular forces interplay with collective coupling in cooperative systems and induce considerable changes in the dynamic polarizability of the first solvation shell. Notice that the employed coupling strength ( = 0.005 au) is, in principle, achievable in few-molecule experimental plasmonic cavities and ensures that the QED-CC computed molecular ground state is fundamentally electronic. Nevertheless, the thermodynamic limit study in Figure 3 and 5 shows that the microscopic response changes of the LP are a collective effect and, therefore, are also achieved for lower light–matter couplings and a larger number of molecules. Such changes arise from a competition between the collective interaction with the optical mode and the local Coulomb interactions, which favors aligned (J-aggregate) or anti-aligned (H-aggregate) patterns in the transition moments. Which effect prevails depends upon the local chemical environment around the solute and the *collective* coupling strength. The described effects are also expected for systems carrying other types of interactions, such as dipole–dipole and hydrogen bonds. Dipole–dipole forces are more long-ranged in comparison to the dispersion interactions of H_2_, and hence, such effects could also be observed at larger distances. On the other hand, hydrogen bonds are shorter ranged, favor a somewhat ordered local structure involving specific functional groups, and can substantially alter both the electronic and vibrational states. Although we do not expect a modification of the hydrogen bond itself (as a result of the low coupling strength), its competition with the transverse fields might alter the solvent response (e.g., by quenching the excitation of the surrounding solvent molecules as in Figure 2) and, thus, promote novel interesting effects in the framework of both electronic strong coupling (ESC) and VSC. Indeed, our work suggests that cooperative coupling to a solvent induces strong modifications in the dynamic polarization of the first solvation shell of the solute. A change in the dynamic polarization of the first solvation shell is then likely to affect the solute dynamics, possibly leading to changes in solvent rearrangement, chemical reactivity, nucleation, aggregation, and ionic conductivity. Such a mechanism could be experimentally investigated using ultrafast ultraviolet (UV) electronic spectroscopy.^31,32,66,67^ Moreover, our results suggest that these effects feature a clear resonance dependence and will be relevant even in the thermodynamic limit and for disordered systems. Hence, our work provides an alternative perspective on cooperative strong coupling and the underlying mechanism that modifies chemistry via changes in the interactions within solvents and aggregates. Several VSC experiments have shown that the resonant coupling can induce modifications in solvent effects^14,36,37^ and assembly,^33−35^ a feature proposed to emerge from collective coupling and structural reorganization rather than significant individual molecular changes.^68^ Longitudinal (coulombic) interactions are ubiquitous and fundamental in understanding chemical effects, and the concepts developed in this letter can help and inspire further discussions on both ESC and VSC. While we fixed the nuclear positions in this work, motivated by a separation of time scales, it is apparent that vibrational strong coupling will require the inclusion of nuclear motion. We can therefore expect that changes in the first solvation shell might result in reorganization of the solvation structure. These results also encourage the development and refinement of multiscale approaches^45,69^ to extend our study to experimentally investigated systems.

## Data Availability

The data of this study is freely available under https://zenodo.org/doi/10.5281/zenodo.10572145.

## Supporting Information Available

Computational details, left and right (QED-)CC local transition densities, ground and excited state densities, absorption spectra, additional model simulations for the J- and H-aggregates, and details on *ab initio* CC and QED-CC as well as the Jaynes–Cummings (JC), TC, Kasha, and TC–Kasha models (PDF)
Transparent Peer Review report available (PDF)
