# Supplementary material for: Collective Strong Coupling Modifies Aggregation and Solvation
Source: J Phys Chem Lett. 2024 Jan 30;15(5):1428–34. doi: 10.1021/acs.jpclett.3c03506 (PMC10860139; doi:10.1021/acs.jpclett.3c03506)
Supplement: Supplementary file 1 — jz3c03506_si_001.pdf [file jz3c03506_si_001.pdf]

Supplemental Information: Collective Strong Coupling Modifies Aggregation and Solvation

Matteo Castagnola,† Tor S. Haugland,† Enrico Ronca,‡ Henrik Koch,*,†,¶ and Christian Schäfer*,§,‖

†*Department of Chemistry, Norwegian University of Science and Technology, 7491 Trondheim, Norway*

‡*Dipartimento di Chimica, Biologia e Biotecnologie, Università degli Studi di Perugia, Via Elce di Sotto, 8,06123, Perugia, Italy*

¶*Scuola Normale Superiore, Piazza dei Cavalieri 7, 56126 Pisa, Italy*

§ *Department of Physics, Chalmers University of Technology, 412 96 Göteborg, Sweden*

‖ *Department of Microtechnology and Nanoscience, MC2, Chalmers University of Technology, 412 96 Göteborg, Sweden*

E-mail: henrik.koch@ntnu.no; christian.schaefer@chalmers.se

S1. Computational Details

The model system we focus on in this letter is a chain of N parallel equidistant molecules. The dimers are arranged either in an H-aggregate parallel-bond or a J-aggregate head-tail configuration as depicted in Figure S1, with constant intermolecular separation d. The central

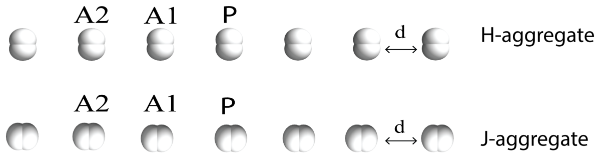

Figure S1: Configurations of the hydrogen aggregates.

dimer, labeled P, is slightly stretched with a bond length of 0.78Å, while the other chain dimers have a bond length of 0.76Å. The chain lies in the XY plane, with the hydrogens aligned on X. The first and second adjacent dimers of P are labeled A1 and A2. The system is modeled at CCSD and QED-CCSD-2 level with the aug-cc-pVDZ basis set (see for a brief description of the methods), and the light-matter coupling constant for the QED calculations is set to 0.005 a.u. with polarization along X. The stretched dimer has an (isolated and undressed) excitation energy of 12.337 eV, slightly lower than the unperturbed excitation of 12.498 eV. The transition dipole moment is along the bonds (X-axis). For each excitation, the transition densities $\varrho_{j0}(x, y, z)$ are computed and saved in a cube file with a grid of 0.1Å spacing. The local transition densities along X $\rho^I_{j0}(x)$ (direction of the transition moment) are computed by numerically integrating the cube on Z and Y around the hydrogen $I =$ P, A1, A2 (within 2.0 Å from each atom)

$$\rho^I_{j0}(x) = \int_{-\infty}^{+\infty} \mathrm{d}z \int_{Y_I - 2.0\,\text{Å}}^{Y_I + 2.0\,\text{Å}} \mathrm{d}y\ \varrho_{j0}(x, y, z) \quad I = \text{P, A1, A2}. \tag{1}$$

All the calculations are computed on a local branch of the $e^{\mathcal{T}}$ program[1]. The Hamiltonian in the dipole approximation and second quan-

tization is

$$H = \sum_{pq} h_{pq} E_{pq} + \frac{1}{2} \sum_{pqrs} g_{pqrs} e_{pqrs} + \sqrt{\frac{\omega}{2}} (\boldsymbol{\lambda} \cdot \boldsymbol{d})_{pq} E_{pq} (b^\dagger + b) \quad (2)$$

$$+ \frac{1}{2} \sum_{pqrs} (\boldsymbol{\lambda} \cdot \boldsymbol{d})_{pq} (\boldsymbol{\lambda} \cdot \boldsymbol{d})_{rs} E_{pq} E_{rs} + \omega \left(b^\dagger b + \frac{1}{2} \right) \quad (3)$$

where $b^\dagger$ (b) is the creation (annihilation) operator for the photon mode of frequency ω, p and q are indices for the one-electron orbitals, E_{pq} and e_{pqrs} are the standard one and two electron singlet excitation operators[2], $\boldsymbol{\lambda}$ is the light-matter coupling vector along the photon polarization, h_{pq} and g_{pqrs} are the one and two electron integrals, and $\boldsymbol{d}$ is the dipole operator.

S2. QED-CC

The QED-CC ansatz is

$$|\text{QED-CC}\rangle = \exp(T) |\text{HF}, 0\rangle, \quad (4)$$

where T is the cluster operator

$$T = T_e + T_p + T_{int}, \quad (5)$$

and $|\text{HF}, 0\rangle$ is the direct product of the QED-HF Slater determinant and the electromagnetic vacuum[3]. The electronic cluster T_e is an electronic excitation operator[2]

$$T_e = \sum_{\mu} t_\mu \tau_\mu \qquad \tau_\mu |\text{HF}\rangle = |\mu\rangle, \quad (6)$$

where $|\mu\rangle$ is an excited electronic determinant. The cluster operator T_p includes pure photonic excitations

$$T_p = \sum_{n=1}^{\bar{n}} \Gamma_n = \sum_{n=1}^{\bar{n}} \gamma_n (b^\dagger)^n, \quad (7)$$

where we considered a single cavity mode. Notice that the bosonic space must be truncated up to a finite number $\bar{n}$ of photon occupations. Finally, the operator T_{int} contains excitations of both matter and photons

$$T_{int} = \sum_{n=1}^{\bar{n}} (S_1^n + S_2^n + \cdots + S_{N_e}^n), \quad (8)$$

where for instance

$$S_1^n = \sum_{ai} s_{ai}^n E_{ai} (b^\dagger)^n \quad (9)$$

$$S_2^n = \frac{1}{2} \sum_{aibj} s_{aibj}^n E_{ai} E_{bj} (b^\dagger)^n. \quad (10)$$

Notice that QED-CC is defined for the Hamiltonian transformed with the QED-HF coherent-state transformation $U_{\text{QED-HF}}$ [3]

$$U_{\text{QED-HF}}^\dagger H \, U_{\text{QED-HF}} = \sum_{pq} h_{pq} E_{pq} + \frac{1}{2} \sum_{pqrs} g_{pqrs} e_{pqrs} + h_{nuc} + \sum_\alpha \sqrt{\frac{\omega_\alpha}{2}} (\boldsymbol{\lambda}_\alpha \cdot (\boldsymbol{d} - \langle \boldsymbol{d} \rangle_{\text{QED-HF}}))(b_\alpha^\dagger + b_\alpha) + \frac{1}{2} \sum_\alpha (\boldsymbol{\lambda}_\alpha \cdot (\boldsymbol{d} - \langle \boldsymbol{d} \rangle_{\text{QED-HF}}))^2 + \sum_\alpha \omega_\alpha b_\alpha^\dagger b_\alpha \quad (11)$$

$$U_{\text{QED-HF}} = \prod_\alpha \exp\left(- \frac{\boldsymbol{\lambda}_\alpha \cdot \langle \boldsymbol{d} \rangle_{\text{QED-HF}}}{\sqrt{2\omega_\alpha}} (b_\alpha^\dagger - b_\alpha) \right), \quad (12)$$

where $\langle \boldsymbol{d} \rangle_{\text{QED-HF}}$ is the mean QED-HF dipole moment. The QED-HF orbitals are obtained by minimizing the energy of the transformed Hamiltonian in Equation 11, with respect to the orbital coefficients. This leads to a modified Fock matrix such that Brillouin's theorem $F_{ia} = 0$ determines the coefficients

$$F_{pq} = F_{pq}^e + \frac{1}{2} \left(\sum_a (\boldsymbol{\lambda} \cdot \boldsymbol{d}_{pa})(\boldsymbol{\lambda} \cdot \boldsymbol{d}_{aq}) - \sum_i (\boldsymbol{\lambda} \cdot \boldsymbol{d}_{pi})(\boldsymbol{\lambda} \cdot \boldsymbol{d}_{iq}) \right), \quad (13)$$

where F_{pq}^e is the standard HF matrix[2], and a, i refer to virtual and occupied orbitals. The QED-CC state is then obtained by projection

of Equation 11 onto the space spanned by the electron-photon excitations $|\mu, n\rangle$[3]

$$\langle \mu, n| e^{-T} H e^{T} |\mathrm{HF}, 0\rangle = 0 \tag{14}$$
$$\langle \mathrm{HF}, 0| H |\text{QED-CC}\rangle = E_{\text{QED-CC}}. \tag{15}$$

The QED-CC dual state is defined as

$$\langle \Lambda| = \langle \mathrm{HF}, 0| + \sum \bar{t}_{\mu n} \langle \mu, n| \, e^{-T}, \tag{16}$$

where $\bar{t}_{\mu n}$ are the Lagrangian multipliers[2,3] obtained by the equation

$$\langle \Lambda| [H, \tau_\mu (b^\dagger)^n] |\text{QED-CC}\rangle = 0. \tag{17}$$

QED-CC is based on a hierarchy of approximations defined by a truncation of the cluster operators, such that the equations are projected only onto a subspace of the full Fock space[2,3]. In this letter, we make use of the QED-CCSD-2 method, in which the electronic cluster includes only single and double excitations

$$T_e = \sum_{ai} t_{ai} E_{ai} + \frac{1}{2} \sum_{aibj} t_{aibj} E_{ai} E_{bj}, \tag{18}$$

and the photonic operator includes single and double photonic creation operators

$$T_p = \gamma_1 b^\dagger + \gamma_2 (b^\dagger)^2. \tag{19}$$

The interaction cluster T_{int} includes all the products between the operators in T_p and T_e. The excited states are defined in the equation of motion (EOM) framework via a CI-like parametrization, which includes the same states as in the cluster operator

$$|\mathcal{R}\rangle = e^{T} \sum_{\mu,n} c_{\mu,n} |\mu, n\rangle \tag{20}$$
$$\langle \mathcal{L}| = e^{-T} \sum_{\mu,n} \tilde{c}_{\mu,n} \langle \mu, n| \, . \tag{21}$$

The left and right excited state coefficients are therefore obtained by diagonalization of the non-hermitian CC-transformed Hamiltonian[2,3]

$$\bar{H} = e^{-T} H e^{T} = \begin{pmatrix} E_{\text{QED-CC}} & \eta_{\mu,n} \\ 0 & A_{\mu n,\nu m} \end{pmatrix} \tag{22}$$

where

$$\eta_{\mu n} = \langle \mathrm{HF}, 0| e^{-T} H e^{T} |\mu, n\rangle \tag{23}$$
$$A_{\mu n,\nu m} = \langle \mu, n| [e^{-T} H e^{T}, \tau_\nu (b^\dagger)^m] |\mathrm{HF}, 0\rangle \, . \tag{24}$$

From the left $\langle \mathcal{L}_k|$ and right $|\mathcal{R}_k\rangle$ eigenvectors of Equation 22 we define the transition and state CC densities

$$D^{k}_{pq} = \langle \mathcal{L}_k| E_{pq} |\mathcal{R}_k\rangle \tag{25}$$
$$D^{0k}_{pq} = \langle \Lambda| E_{pq} |\mathcal{R}_k\rangle \tag{26}$$
$$D^{k0}_{pq} = \langle \mathcal{L}_k| E_{pq} |\text{QED-CC}\rangle \, , \tag{27}$$

from which we can compute excited and transition properties such as the oscillator strengths. Notice that since the CC theory is not hermitian, the left and right transition densities are not adjoints.

S3. Simple models

A. Jaynes-Cummings model

The coupling of an optical mode with a single excitation is often described by the Jaynes-Cummings Hamiltonian H_{JC}, where the molecule is described as a two-level system coupled to the photon by an electric dipole interaction in the rotating wave approximation[4]

$$H_{JC} = \omega_{mol} |e\rangle \langle e| + \tilde{g}_0 \left(|g\rangle \langle e| \, b^\dagger + |e\rangle \langle g| \, b \right) + \omega_{ph} b^\dagger b. \tag{28}$$

In Equation 28, $b^\dagger$ (b) is the creation (annihilation) operator for the photons, $|g\rangle$ and $|e\rangle$ are the ground and excited molecular states, with energy difference ω_{mol} (the ground state energy is arbitrarily set to zero), and $\tilde{g}_0$ is the coupling strength

$$\tilde{g}_0 = \sqrt{\frac{\omega_{ph}}{2}} \boldsymbol{d}_{ge} \cdot \boldsymbol{\lambda}, \tag{29}$$

where $\boldsymbol{\lambda}$ is the fundamental light-matter coupling strength directed along the polarization of the field and $\boldsymbol{d}_{ge}$ is the transition dipole moment. For the Hamiltonian in Equation 28, because of the rotating wave approximation, the

excitation number operator $\hat{\eta}$

$$\hat{\eta} = |e\rangle\langle e| + \sum_n |n\rangle\langle n|, \tag{30}$$

where $|n\rangle$ is the electromagnetic n-photon number state, commutes with H_{JC} and therefore the Hamiltonian is block diagonal in the subspaces $\{|e\rangle|n-1\rangle, |g\rangle|n\rangle\}$. We then focus on the "vacuum Rabi splitting," with the subspace containing at most one photon excitation (excitation number $\eta = 1$). Then, in the resonant case $\omega_{mol} = \omega_{ph}$, the eigenstates of H_{JC} are the cavity polaritons arising from the symmetric and antisymmetric combinations of the photon and the molecular excitation

$$|\pm\rangle = \frac{1}{\sqrt{2}}\big(|e\rangle|0\rangle \pm |g\rangle|1\rangle\big), \tag{31}$$

where $|1\rangle$ and $|0\rangle$ are respectively the one-photon and vacuum electromagnetic states. The upper and lower polaritons are separated by the vacuum Rabi splitting Ω

$$\Omega = 2\tilde{g}_0, \tag{32}$$

while the ground state is the unchanged molecular ground state in the electromagnetic vacuum $|g\rangle|0\rangle$. In the general subspace of excitation number $\eta = \tilde{\eta}$, the Rabi splitting scales as $\sqrt{\tilde{\eta}}$.

B. Tavis-Cummings model

In the case of N identical molecules coupled to the same photonic mode, the TC or Dicke Hamiltonian reads[5,6]

$$\begin{aligned} H_{TC} = &\omega_{mol}\sum_{k=1}^{N}|e_k\rangle\langle e_k| + \omega_{ph}b^\dagger b \\ &+\tilde{g}_0\sum_{k=1}^{N}\big(|g_k\rangle\langle e_k|\, b^\dagger + |e_k\rangle\langle g_k|\, b\big), \end{aligned} \tag{33}$$

where the molecules are assumed to have the same excitation energy ω_{mol}, the same coupling strength $\tilde{g}_0$, and the index k runs over the different replicas. We now assume the system to be resonant $\omega_{ph} = \omega_{mol} = \omega$, and the Hamiltonian in the first excited manifold is

$$\begin{pmatrix} \omega & \tilde{g}_0 & \tilde{g}_0 & \tilde{g}_0 & \cdots \\ \tilde{g}_0 & \omega & 0 & 0 & \cdots \\ \tilde{g}_0 & 0 & \omega & 0 & \cdots \\ \vdots & \vdots & \ddots & \ddots & \ddots \end{pmatrix}. \tag{34}$$

The system is more easily diagonalized by using the collective state basis for matter

$$|G\rangle = |g_1, g_2, g_3, \ldots\rangle \tag{35}$$

$$|E\rangle = \frac{1}{\sqrt{N}}\sum_{k=1}^{N}|e_k, g_{j\neq k}\rangle \tag{36}$$

$$|D_j\rangle = \frac{1}{\sqrt{N}}\sum_{k=1}^{N}e^{i\frac{2\pi}{N}j}|e_k, g_{j\neq k}\rangle \quad j = 1, \ldots, N-1. \tag{37}$$

The states $|D_j\rangle|0\rangle$ are eigenvectors of H_{TC} with eigenvalues ω and are referred to as *dark states* since they have no contributions from the excited photon field. The upper and lower polaritons $|\pm\rangle$ are

$$|\pm\rangle = \frac{1}{\sqrt{2}}\Big(|G\rangle|1\rangle \pm |E\rangle|0\rangle\Big) \tag{38}$$

and have contributions from both the photon field and the N molecules, and are separated by a Rabi splitting Ω_{TC}

$$\Omega_{TC} = \sqrt{N}\Omega, \tag{39}$$

that is, the Rabi splitting scales as the squared root of the number of coupled replicas. Notice that the sign of the transition dipole moment is arbitrary. If we flip the sign for one of the replicas, which we can assume without loss of generality to be the first one, the Hamiltonian reads

$$\begin{pmatrix} \omega & -\tilde{g}_0 & \tilde{g}_0 & \tilde{g}_0 & \cdots \\ -\tilde{g}_0 & \omega & 0 & 0 & \cdots \\ \tilde{g}_0 & 0 & \omega & 0 & \cdots \\ \vdots & \vdots & \ddots & \ddots & \ddots \end{pmatrix}. \tag{40}$$

The matrices in Equation 34 and Equation 40 are similarly equivalent, connected by the trans-

formation

$$\begin{pmatrix} 1 & 0 & 0 & 0 & \dots \\ 0 & -1 & 0 & 0 & \dots \\ 0 & 0 & 1 & 0 & \dots \\ \vdots & \ddots & \ddots & \ddots & \ddots \end{pmatrix} \tag{41}$$

Therefore, the new polaritonic states have the coefficients of $|e_1, g_2, g_3, \dots\rangle$ with a flipped sign compared to the other representation. This ensures that all of the transition dipoles constructively sum and not cancel with each other. Therefore, in the polaritons, all of the transition moments must be aligned to maximize the electric-dipole interaction with the cavity field. On the other hand, in the dark states, the phases of the molecular excitations are such that the transition moment sum vanishes.

C. Disordered TC

The molecular disorder affects both the molecular energies (inhomogeneous broadening) and the coupling strengths. Following Houdré et al.[7], the disordered TC Hamiltonian is

$$H_{DTC} = \sum_{k=1}^{N} \omega_k |e_k\rangle\langle e_k| + \sum_{k=1}^{N} \tilde{g}_k \big(|g_k\rangle\langle e_k|\, b^\dagger + |e_k\rangle\langle g_k|\, b \big) + \omega_{ph} b^\dagger b, \tag{42}$$

and the polaritonic energies E are defined by the equation

$$\sum_{k=1}^{N} \frac{\tilde{g}_k^2}{(\omega_k - E)} - (\omega_{ph} - E) = 0. \tag{43}$$

Each molecule is then endowed with a different excitation energy (inhomogeneous broadening) and coupling strength (orientational and positional disorder). If we disregard the inhomogeneous broadening of the excitations and set $\omega_k = \omega_{ph} = \omega$, the orientational and positional disorder only affects the coupling strengths $\tilde{g}_k$ via the scalar product $\boldsymbol{\lambda} \cdot \boldsymbol{d}_{ge}$ and the system have again $N-1$ degenerate states of energy ω and two polaritonic excitations

$$|\pm\rangle = \frac{1}{\sqrt{2}} \Bigg(|G\rangle\,|0\rangle \pm \sum_{k=1}^{N} \frac{\tilde{g}_k}{\sqrt{\sum_{i=1}^{N} \tilde{g}_i^2}} |e_k g_{j\neq k}\rangle\,|1\rangle \Bigg) \tag{44}$$

with a Rabi splitting

$$\Omega_{DTC} = 2\sqrt{\sum_{k=1}^{N} \tilde{g}_k^2}. \tag{45}$$

Therefore, the Rabi splitting is determined by the *quadratic average* of the different single-molecule couplings, and the contribution of each excited molecule to the polaritons is weighted by its coupling[7]. Notice that from Equation 44 we see that if all the molecules have the same module coupling $|\tilde{g}_k| \equiv \tilde{g}$ each contribution is weighted by $\tilde{g}_k/\tilde{g} = sgn(\tilde{g}_k)$, that is, it is weighted by the sign of the coupling strength so that all of the contributions from the transition moments add up. The Rabi splitting is smaller than in the absence of disorder, but the overall polariton effects are the same.
If the inhomogeneous broadening of the molecular excitation is introduced such that

$$\omega_k = \omega + \epsilon_k \qquad \epsilon_k \ll \omega, \tag{46}$$

we can resort to a perturbative solution for the polaritonic energies as discussed in Ref.[7]

$$\omega_\pm = \omega \pm \sqrt{\sum_k \tilde{g}_k^2} - \frac{1}{2} \frac{\sum_k \tilde{\epsilon}_k g_k^2}{\sum_k \tilde{g}_k^2} + O(\epsilon^2). \tag{47}$$

The remaining $N-1$ states are such that

$$E_k \in [\omega_k, \omega_{k+1}] \quad \omega_k < \omega_{k+1}, \quad k = 1, \dots, N-1. \tag{48}$$

Therefore, for the photon coupling to an inhomogeneously broadened set of oscillators, the TC predicts that the Rabi splitting is unchanged and, to first-order, the eigenstates are the same as in Equation 44[7].

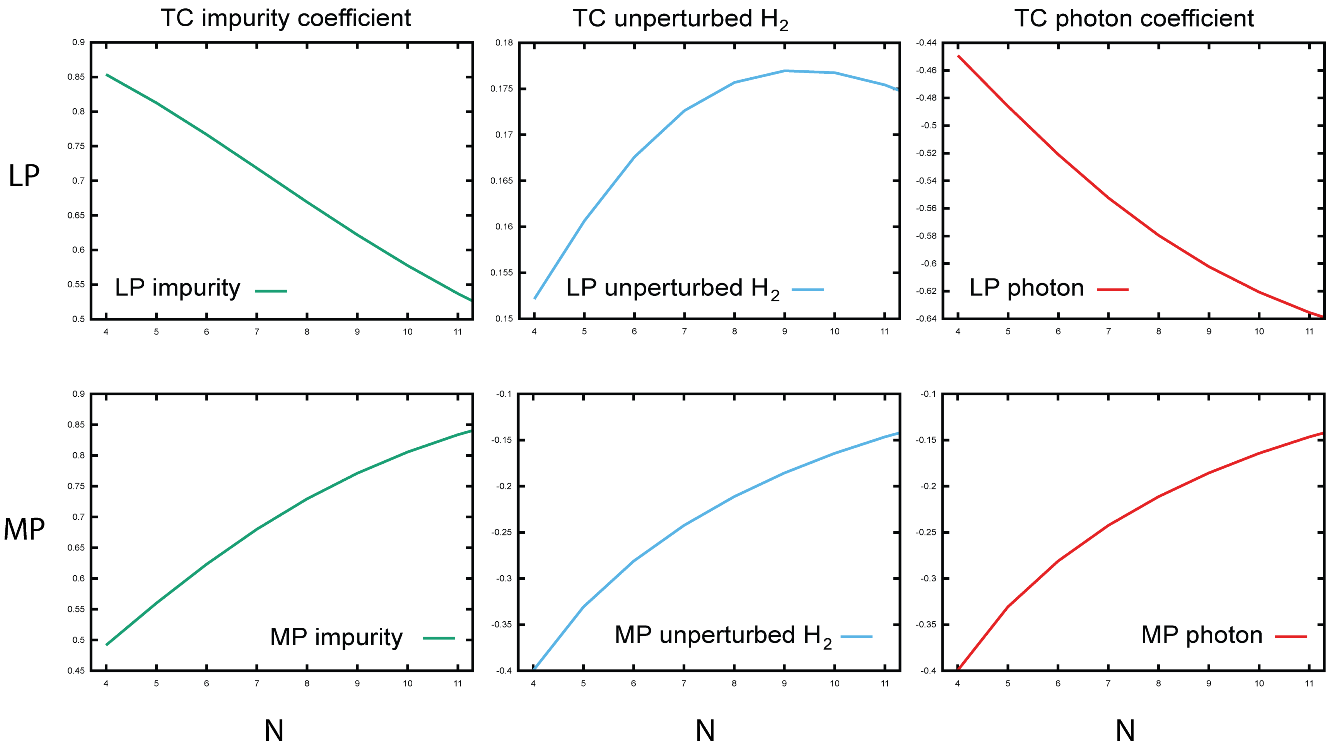

Figure S2: In the LP (upper panels), the perturbed and unperturbed H_2 excitations are in phase (same sign of the CI coefficients) while in antiphase with the photon contribution. The coefficient of the impurity decreases with N, and the photon character becomes more and more relevant, while the unperturbed dimer contribution reaches a maximum around $N = 9$ and then decreases again. In the MP (lower panels), the perturbed dimers are in antiphase with the photon and the unperturbed H_2 molecules. The polariton becomes more localized on the impurity while the photon and the impurity contribution approach zero.

D. TC analysis of the hydrogen chain

For the $(H_2)_N$ system under study, the TC Hamiltonian reads

$$\begin{aligned} H'_{TC} =& \omega \sum_{k=1}^{N-1} |e_k\rangle \langle e_k| + \omega b^\dagger b + \omega' |e'\rangle \langle e'| \\ &+ \tilde{g}_0 \sum_{k=1}^{N-1} \big(|g_k\rangle \langle e_k| \, b^\dagger + |e_k\rangle \langle g_k| \, b \big) \\ &+ \tilde{g}_0{}' \big(|g'\rangle \langle e'| \, b^\dagger + |e'\rangle \langle g'| \, b \big), \end{aligned} \tag{49}$$

where the primed quantities refer to the stretched dimer and the photon is resonant with the unperturbed H_2 molecules. The eigenstates of H'_{TC} are obtained by diagonalizing the matrix

$$\begin{pmatrix} \omega & \tilde{g}_0{}' & \tilde{g}_0 & \tilde{g}_0 & \cdots \\ \tilde{g}_0{}' & \omega' & 0 & 0 & \cdots \\ \tilde{g}_0 & 0 & \omega & 0 & \cdots \\ \vdots & \vdots & \ddots & \ddots & \ddots \end{pmatrix}. \tag{50}$$

In Figure S2, we plot the coefficient of the photon and H_2 excited states that contribute to the LP and MP from equation Equation 50 for $4 \leq N \leq 11$. Notice that different scales are applied to each panel. In the LP, the perturbed and unperturbed H_2 excitations are in phase (same sign of the CI coefficients), and are in antiphase with the photon contribution. The coefficient of the impurity decreases with N and the photon character becomes more and more relevant, while the unperturbed dimer contribution reaches a maximum around $N = 9$ and then decreases again. Notice that, since in the TC

the intermolecular forces are completely disregarded, each unperturbed H_2 will give the same contribution to the polaritonic states. The differences observed in the dimers A1 and A2 shown in the letter are due to the intermolecular forces. In the MP, the perturbed dimers are in antiphase with the photon and the unperturbed H_2 molecules. The polariton becomes more localized on the impurity while the photon and the impurity contribution approach zero.

S4. Hydrogen aggregates electronic structure properties (no QED)

A. H-aggregates

In Figure S3, we show the electronic (undressed) absorption spectra of the $(H_2)_{N\geq4}$ H-aggregate (see Figure S1) for different intermolecular separations. As the system enlarges, the upper excitation E2 is blueshifted due to the intermolecular forces; therefore, the shift is less significant for large intermolecular distances. Notice that E2 gains oscillator strength with N, suggesting that all the unperturbed dimers contribute to this excitation. The excitation E1 is almost independent of the chain length, and it redshifts and loses intensity for larger longitudinal couplings (shorter intermolecular separations). Therefore, this suggests that E1 is mainly associated with the perturbed dimers P, and the nearest dimers produce an effective screening of its transition dipole moment. The system also possesses $N-2$ quasi-dark states, which gain some oscillator strength due to the intermolecular forces. The contribution of each dimer to E1 and E2 can be extracted from the transition densities integrated around each H_2 as explained in section and section .

In Figure S4 and Figure S5, we show the left and right local transition densities of E1 for P, A1, and A2 for 5 Å and 6 Å intermolecular separation, respectively. As for the absorption spectra of Figure S3, the local densities for E1 are independent of N and are mainly localized on P. Due to the intermolecular forces, an exci-

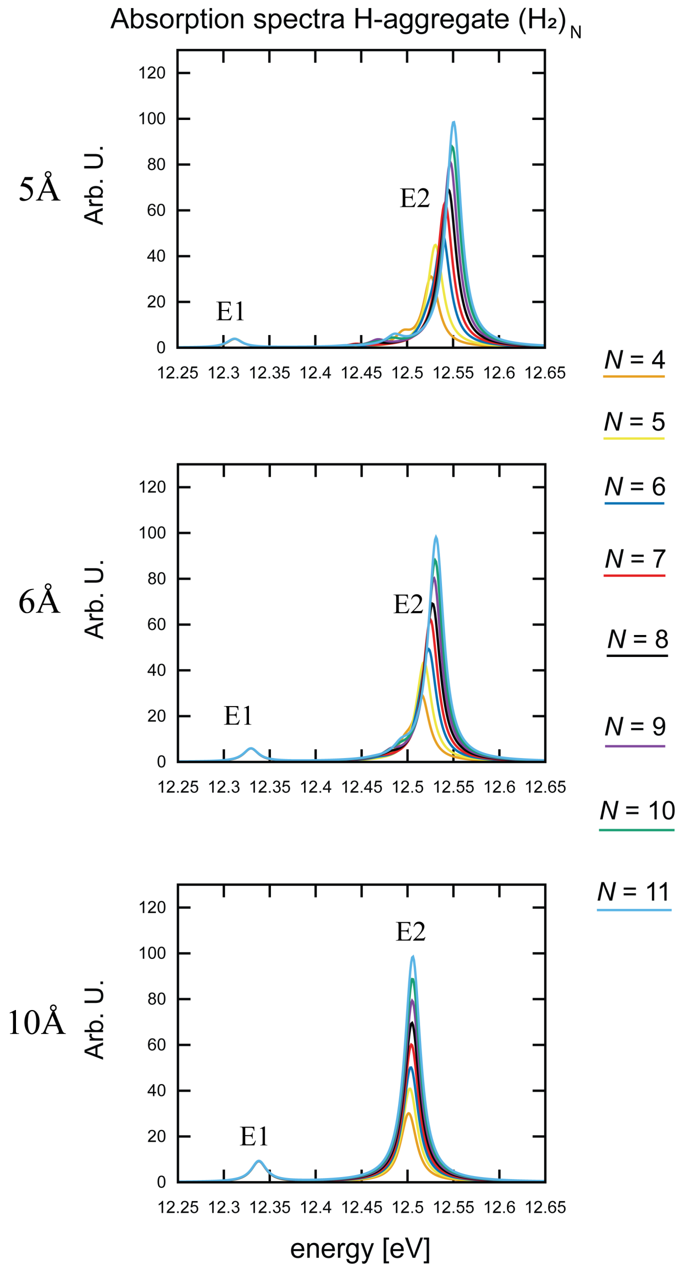

Figure S3: Absorption spectra for an H-aggregate $(H_2)_{N\geq4}$ for 5 Å (upper panel), 6 Å (central panel), and 10 Å (lower panel) intermolecular separation. The excitation E2 depends on the number of dimers in the chain, and it blueshifts with stronger intermolecular forces and larger N. The excitation E1 is mainly localized on P and its neighbor dimers and is almost independent of N, while it redshifts with the longitudinal forces. N-2 quasi-dark states gain oscillator strength due to the intermolecular couplings, showing smaller shoulder peaks near E2.

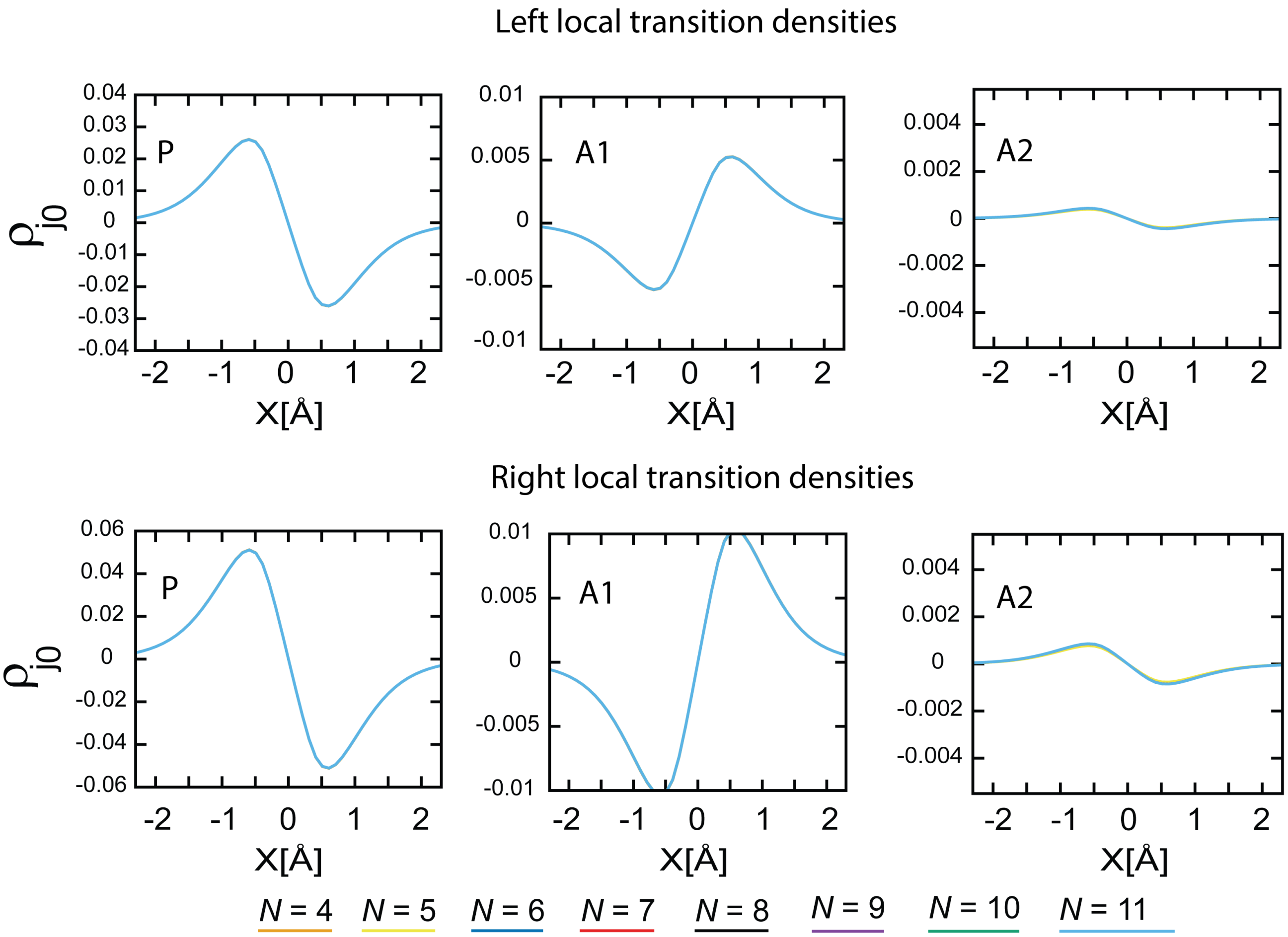

Figure S4: Local left and right transition densities for E1 for an intermolecular separation of 5 Å in an H-aggregate configuration (parallel-bond arrangement, see Figure S1). The excitation is independent of the chain length N and mainly localized on P, A1, and A2 with decreasing contributions. The local transition dipole moments are anti-aligned, producing an effective screening of the excitation compared to the isolated P.

tation of P induces a response on its neighbor dimers, with a less important effect for more distant dimers. As the intermolecular distances become larger, the excitation becomes more localized on P and less on the other dimers, as expected when decreasing the intermolecular couplings. Adjacent molecular transition dipoles are anti-aligned, partially screening the excitation, resulting in smaller oscillator strength than the isolated P. Notice that for A2, we only plot the local densities for $N \geq 6$ since for $N = 4, 5$, its "first solvation shell" is incomplete. Therefore, its properties have significant variations from $N = 4$ to 6 due to local intermolecular forces.

In Figure S6 and Figure S7, we show the left and right local transition densities of E2 for P, A1, and A2 for 5 Å and 6 Å intermolecular separation, respectively. In this case, the local transition dipoles are all aligned, and all the dimers in the chain give a similar contribution. Therefore, the properties of E2 strongly depend on the length of the whole chain and the intermolecular forces acting on each dimer. With larger intermolecular distances, the contribution of P to E2 decreases, while the other dimers maintain a similar local transition density.

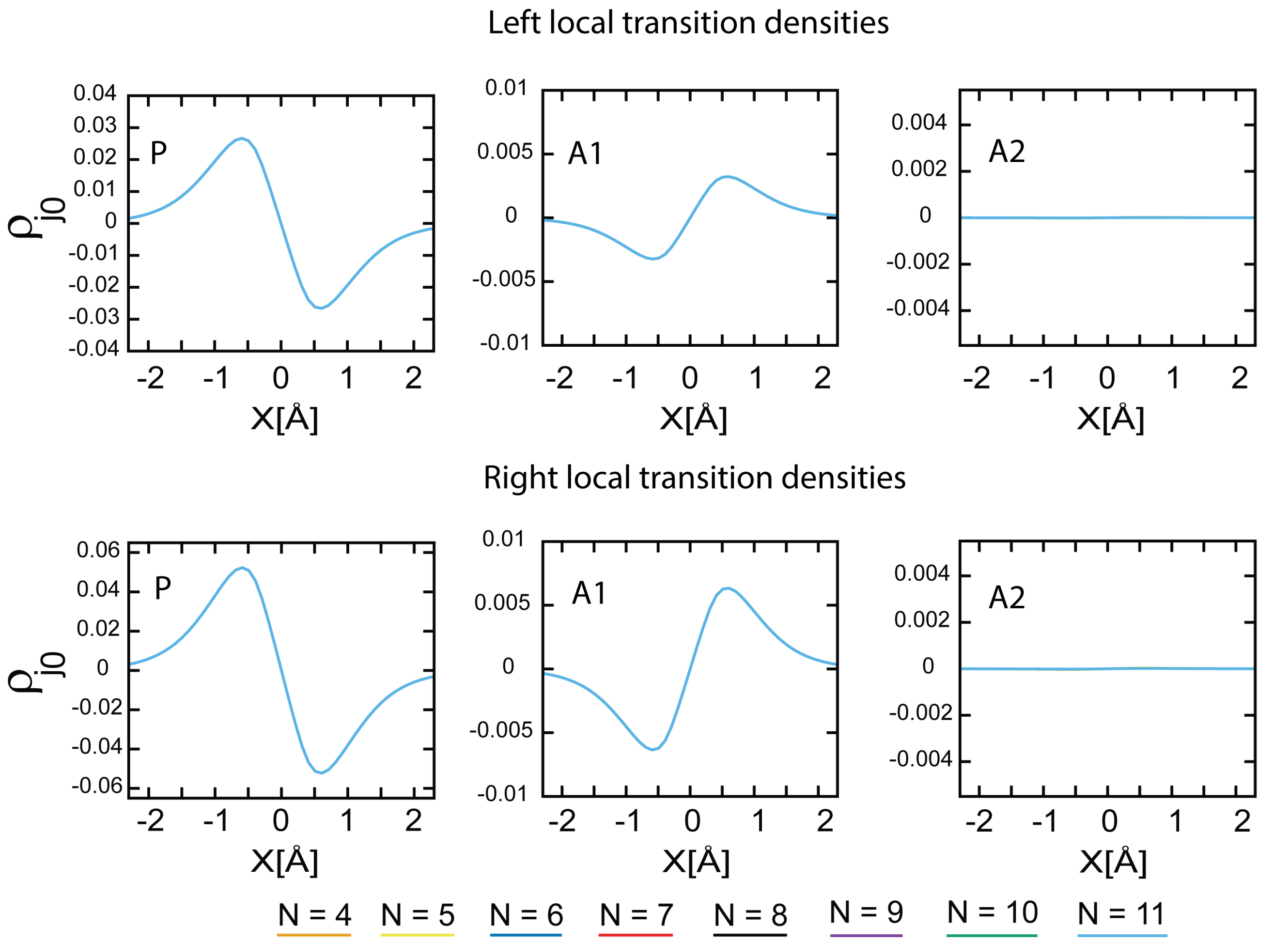

Figure S5: Local left and right transition densities for E1 for an intermolecular separation of 6 Å in an H-aggregate configuration (parallel-bond arrangement, see Figure S1). The excitation is almost independent of N and mainly localized on P, A1, and A2 with decreasing contributions. Compared to Figure S4, the intermolecular forces are less relevant, and therefore, the excitation is more localized on P and less on A1 and A2. Still, the overall anti-alignment pattern of the molecular transition dipoles is maintained.

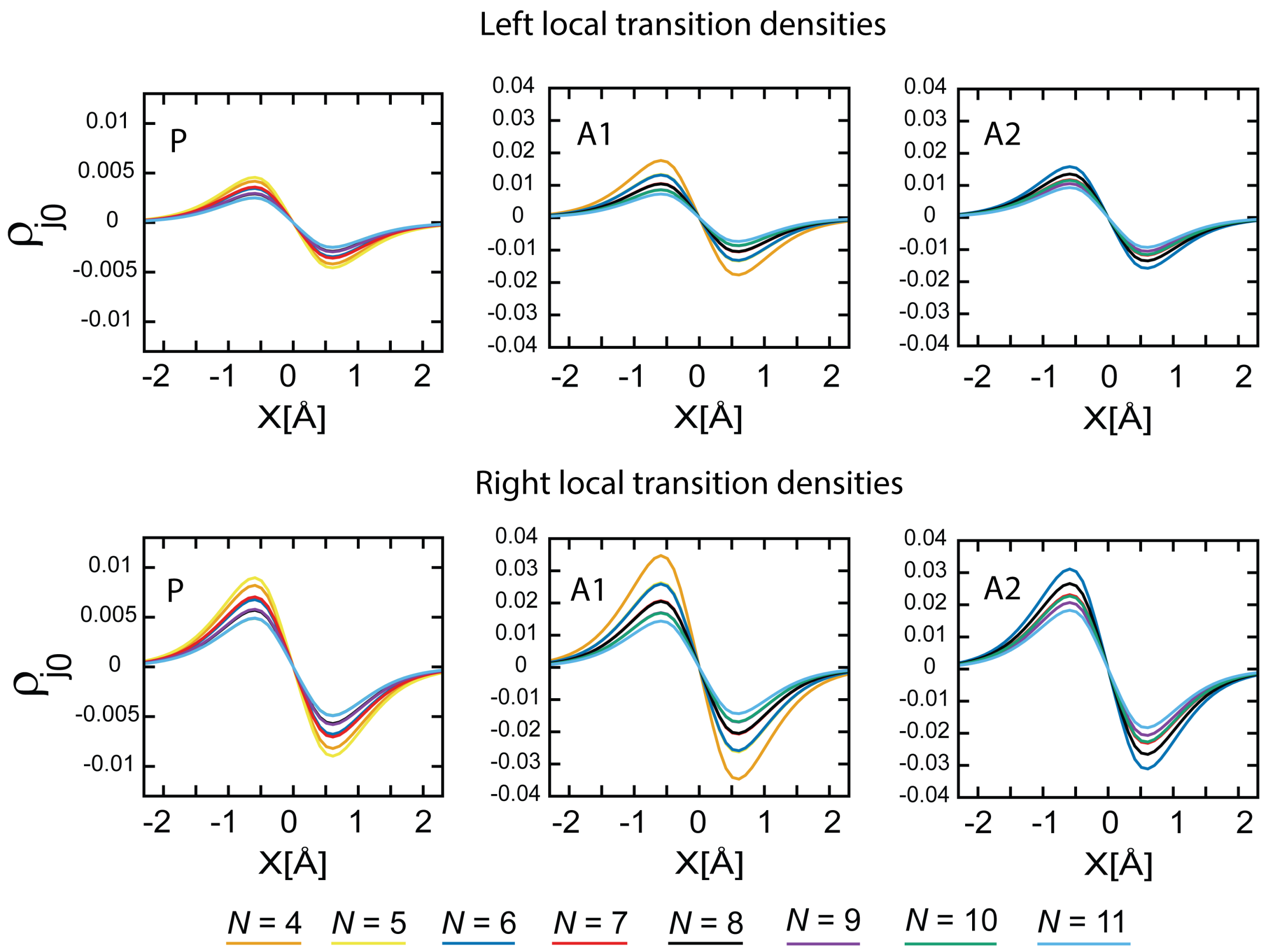

Figure S6: Local left and right transition densities for E2 for an intermolecular separation of 5 Å in an H-aggregate configuration (parallel-bond arrangement, see Figure S1). The excitation blueshifts and gains oscillator strength with the chain length N and is delocalized over the whole aggregate with a larger contribution from the unperturbed molecules. The local transition dipole moments are all aligned.

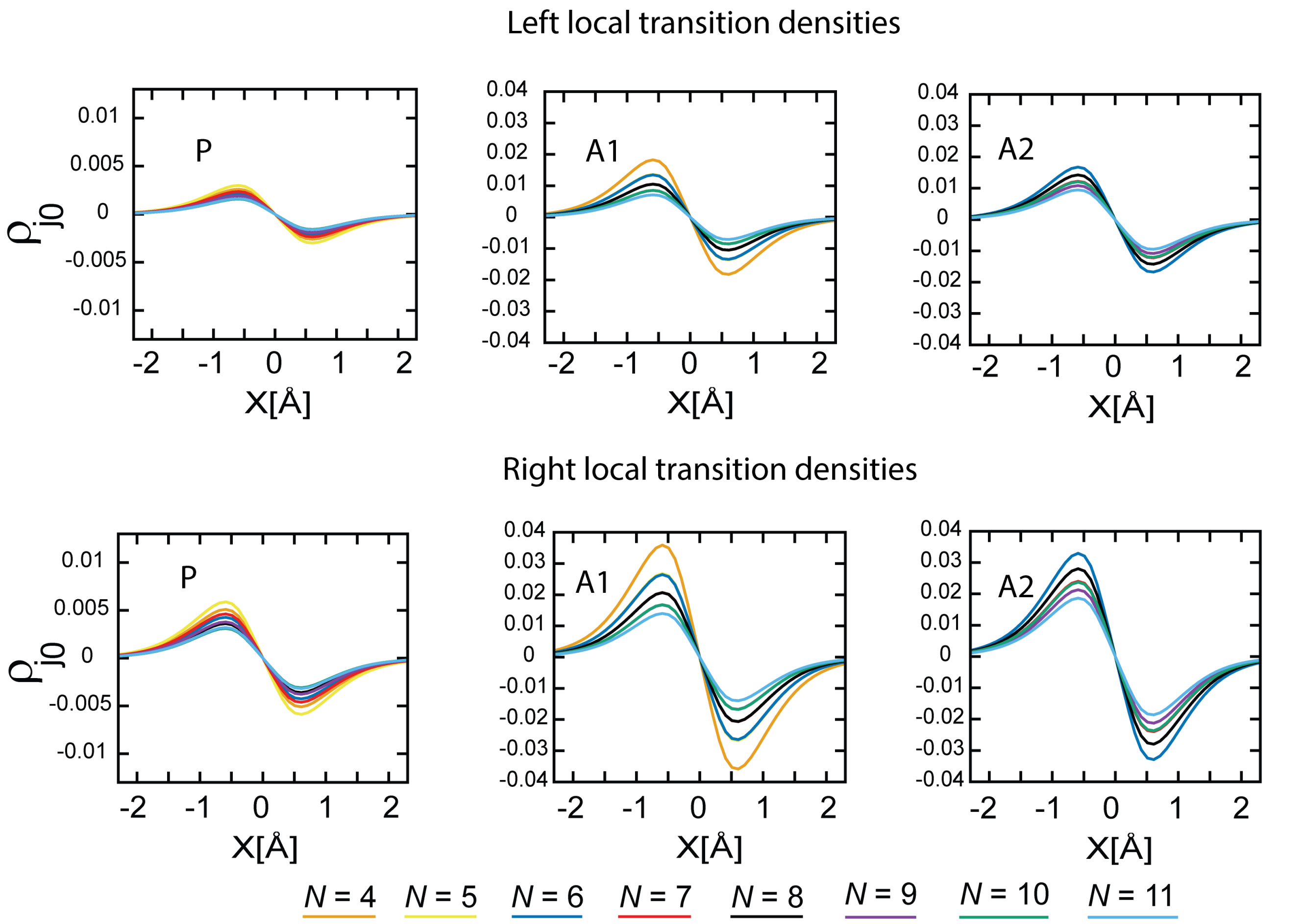

Figure S7: Local left and right transition densities for E2 for an intermolecular separation of 6 Å in an H-aggregate configuration (parallel-bond arrangement, see Figure S1). The excitation blueshifts and gains oscillator strength with the chain length N. It is delocalized over the whole aggregate, with a more significant contribution from the unperturbed molecules. The local transition dipole moments are all aligned, and compared to Figure S6, there is a smaller contribution from P and a similar contribution from the rest of the chain. For larger distances, that is, smaller intermolecular forces, the blueshift is less pronounced.

B. J-aggregates

Since the Coulomb forces are strongly directional, modifying the local arrangement severely affects the system's properties. In Figure S8, we show the absorption spectra for a head-tail configuration (J-aggregate, see Figure S1) for different intermolecular distances. Compared to the H-aggregate of Figure S3, the effects of the intermolecular forces are more pronounced, as seen from the more complicated structure of the spectra for 5 Å and 6 Å. For d = 5 Å and 6 Å, the N-2 quasi-dark state gained relevant oscillator strength, and the appreciable changes of E1 from $N = 4$ to $N = 6$ suggest that the dimer A2 and even father away give a significant contribution to E1. For larger intermolecular separations, E1 is almost independent of N as in Figure S3. The excitations E1 and E2 redshift for increasing chain length and decreasing intermolecular separation, while E2 blueshifts for the H-aggregate. Unlike the H-aggregate case, E1 gains oscillator strength from embedding in the H_2 chain, while E2 loses intensity with increasing intermolecular forces. Since the excitation energy E1 shows little dependence on the chain length, we deduce that it is still mainly localized on P, A1, and A2. E2 becomes more intense with N, suggesting that all dimers contribute to this excitation. The differences between the H- and J- aggregates result from the different effects of the local Coulomb (longitudinal) forces on the dimers. In Figure S9 and Figure S10, we report the left and right local transition densities of E1 for P, A1, and A2 for 6 Å and 8 Å intermolecular separation, respectively. Contrary to the H-aggregate arrangement, in this configuration, the local transition moments are all aligned, increasing the excitation's intensity. The densities are almost independent of the chain length N, and the contribution of the neighbor dimers A1 and A2 increases with decreasing intermolecular separation.

In Figure S11 and Figure S12, we show the left and right local transition densities of E2 for P, A1, and A2 for 6 Å and 8 Å intermolecular separation, respectively. In this configuration, the transition dipole moment of P is anti-aligned with the rest of the chain. As for the H-aggregates, E2 is delocalized over the whole chain with a more relevant contribution from the unperturbed dimers, and the intermolecular forces tend to increase the contribution of P. However, P's local transition dipole moment is anti-aligned with the rest of the hydrogens. Therefore, the alignment pattern of P and A1 in E1 and E2 is swapped compared to the H-aggregate arrangement. This reflects the severe consequences that intermolecular forces can have on the local properties of the system.

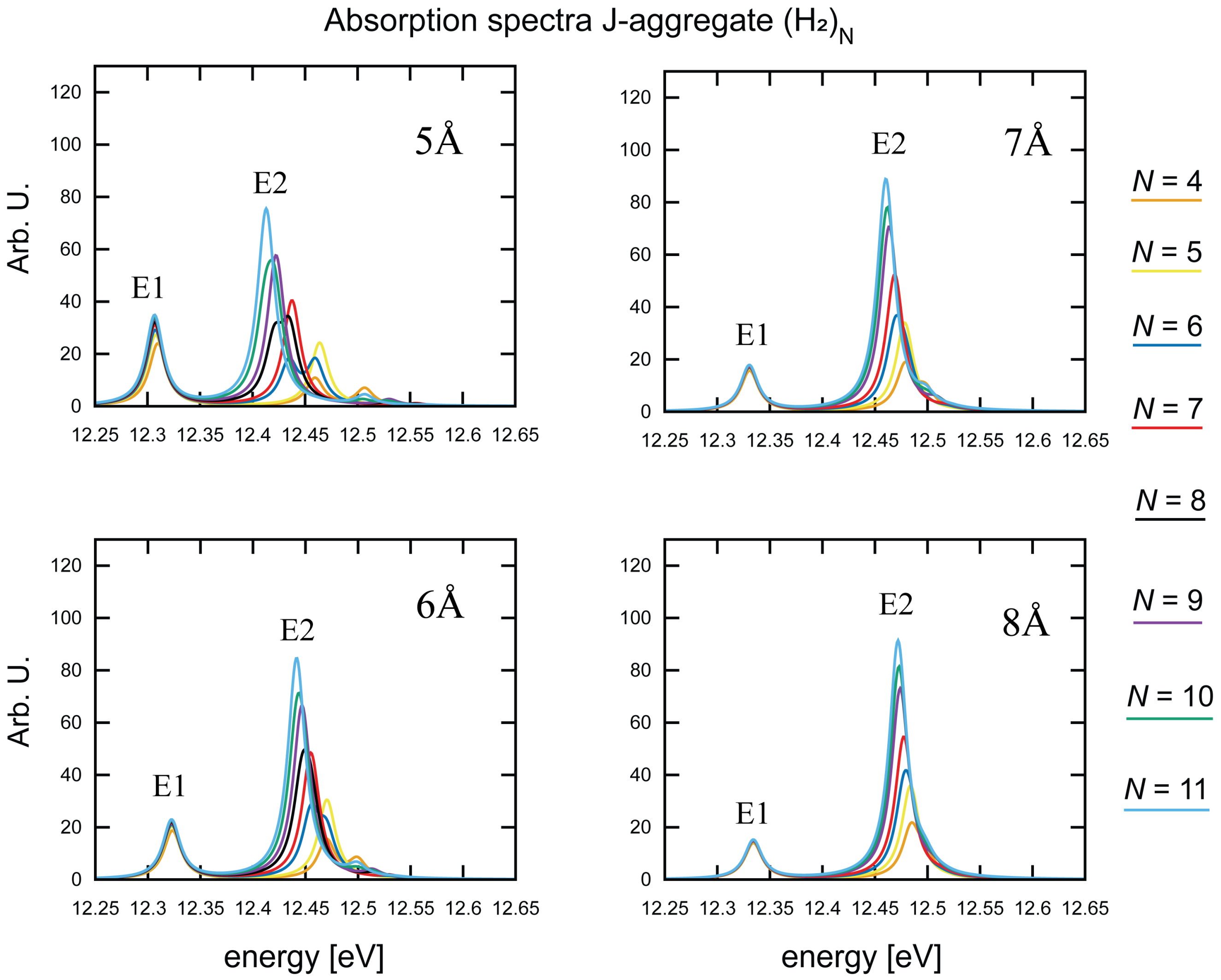

Figure S8: Absorption spectra for a head-tail aggregate (J-aggregate, see Figure S1) $(H_2)_{N\geq 4}$ for 5 Å (upper left panel), 6 Å (lower left panel), 7 Å (upper right panel), and 8 Å (lower right panel) intermolecular separation. The excitation E2 depends on the number of dimers and redshifts with stronger intermolecular forces and larger N. The excitation E1 is mainly localized on P and its neighbor dimers with little dependence N and redshifts with the longitudinal forces. N-2 quasi-dark states gain oscillator strength due to the intermolecular couplings, showing peaks near E2.

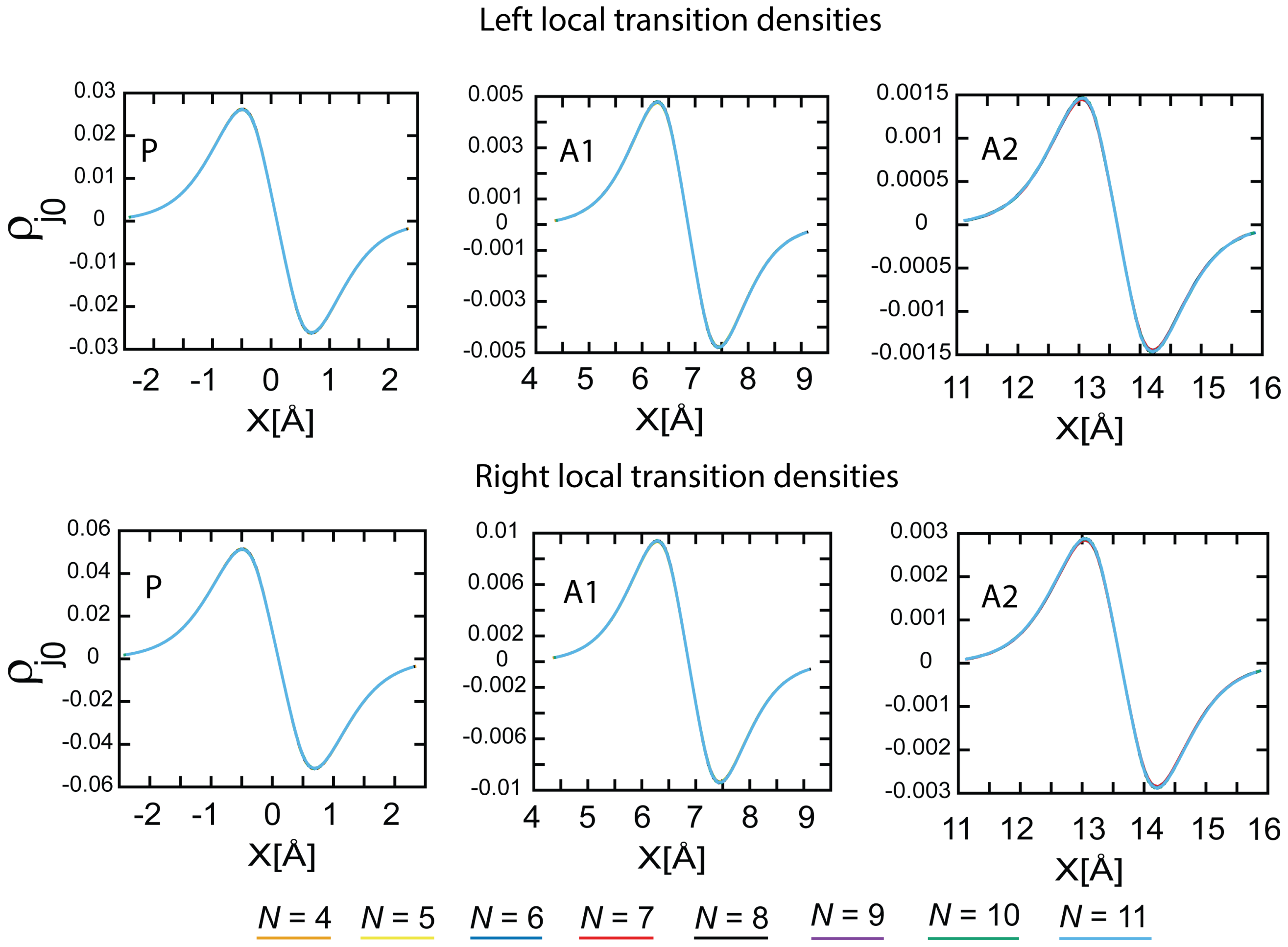

Figure S9: Local left and right transition densities for E1 for an intermolecular separation of 6 Å in a J-aggregate configuration (head-tail arrangement, see Figure S1). The excitation is independent of the chain length N and mainly localized on P, A1, and A2 with decreasing contributions. The local transition dipole moments are aligned, increasing the oscillator strength compared to the isolated P.

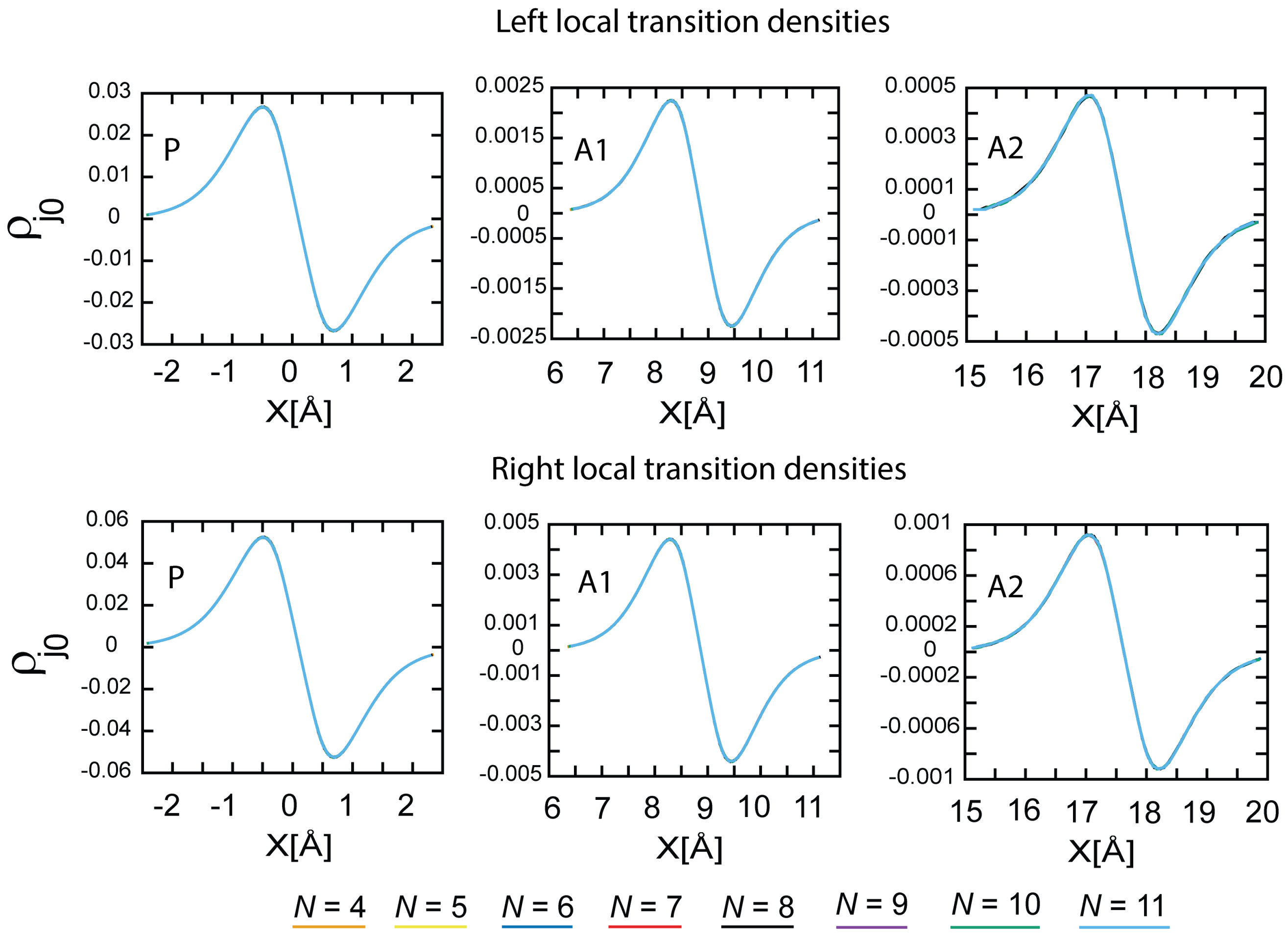

Figure S10: Local left and right transition densities for E1 for an intermolecular separation of 8 Å in a J-aggregate configuration (head-tail arrangement, see Figure S1). The excitation is almost independent of N and mainly localized on P, A1, and A2 with decreasing contributions. Compared to Figure S9, the intermolecular forces are less relevant, and therefore, the excitation is more localized on P and less on A1 and A2, but the overall alignment pattern of the molecular transition dipoles is maintained.

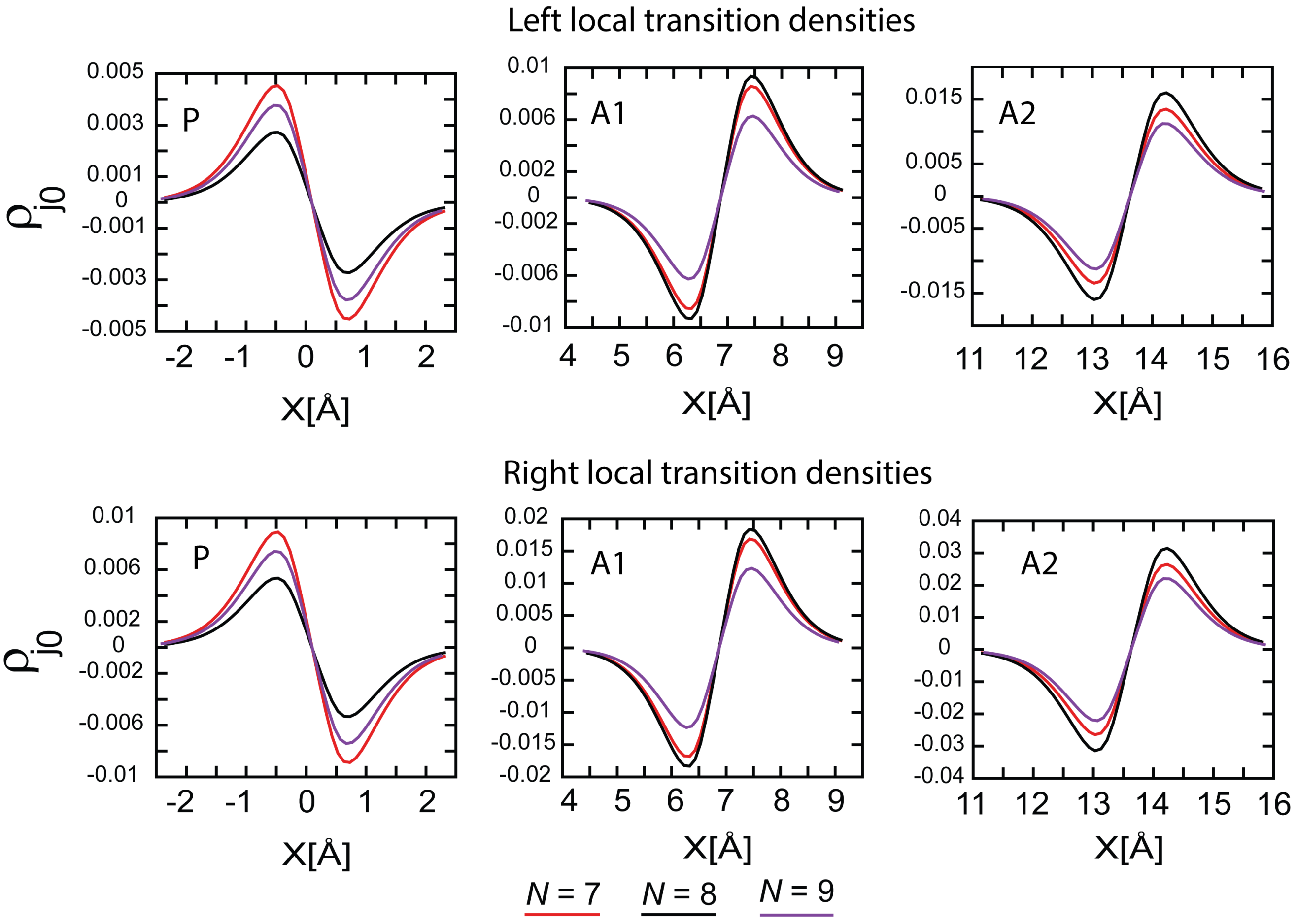

Figure S11: Local left and right transition densities for E2 for an intermolecular separation of 6 Å in a J-aggregate configuration (head-tail arrangement, see Figure S1). The excitation delocalized over the whole chain with a larger contribution from the unperturbed dimers. The local transition dipole moments are anti-aligned, contrary to E2 for the H-aggregate configuration.

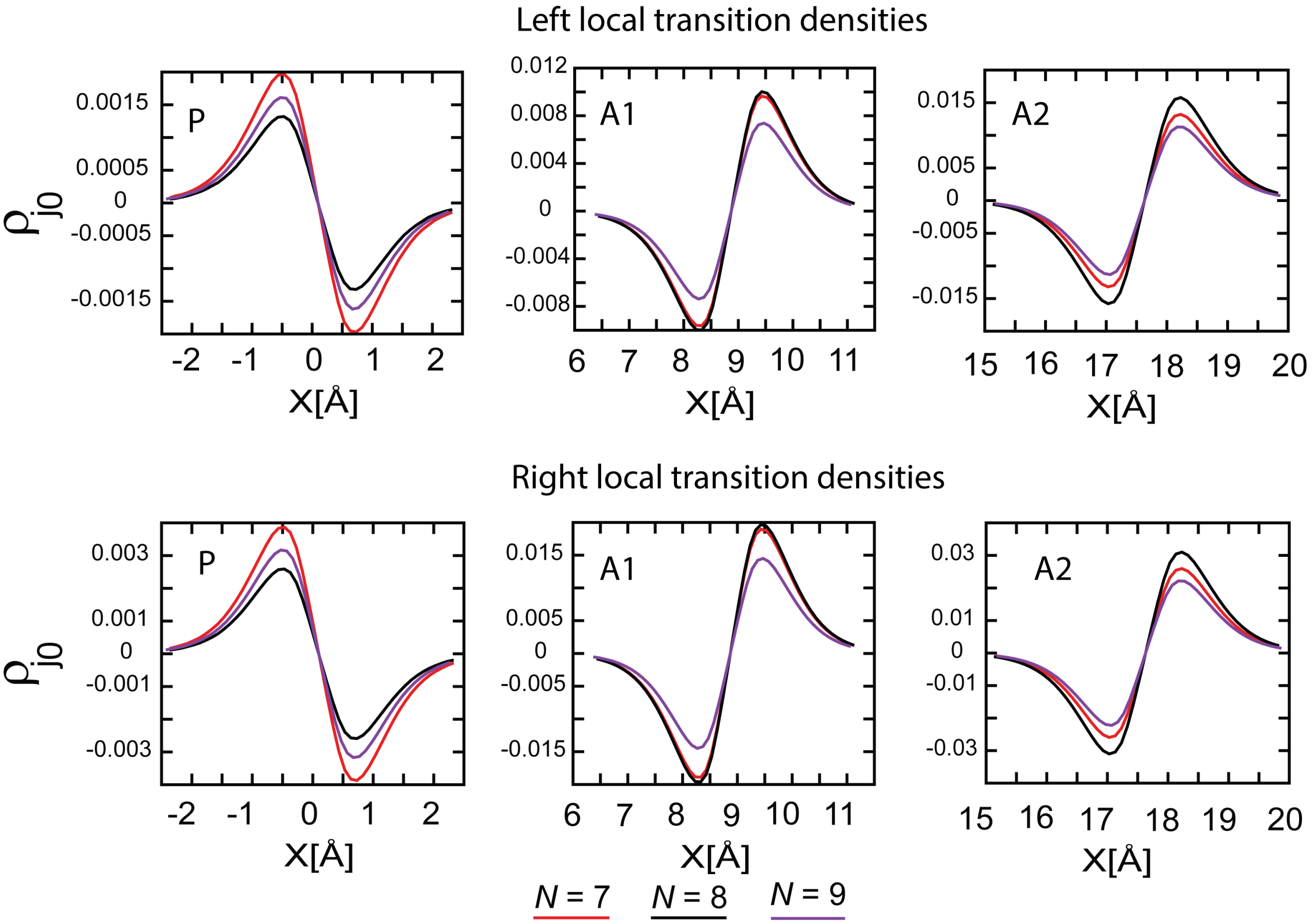

Figure S12: Local left and right transition densities for E2 for an intermolecular separation of 8 Å in a J-aggregate configuration (head-tail arrangement, see Figure S1). The excitation delocalized over the whole chain with a larger contribution from the unperturbed dimers. The local transition dipole moments are anti-aligned, contrary to E2 for the H-aggregate configuration, and compared to Figure S11 there is a smaller contribution from P and a similar contribution from the rest of the chain.

C. Kasha-Frenkel model for H- and J-aggregates

The spectral properties of the H- and J-aggregates can be qualitatively understood using the simple Kasha-Fernkel model[8–12]. The interplay between the excitation of different molecules is modeled by a dipole-dipole interaction V_{AB} between their transition dipoles

$$V_{AB} = \frac{(\boldsymbol{\mu}_A \cdot \boldsymbol{\mu}_B) R_{AB}^2 - 3(\boldsymbol{\mu}_A \cdot \boldsymbol{R}_{AB})(\boldsymbol{\mu}_B \cdot \boldsymbol{R}_{AB})}{\epsilon R_{AB}^5}, \quad (51)$$

where ϵ is the medium dielectric constant, A and B label the two interacting molecules with isolated transition dipole moments $\boldsymbol{\mu}_A$ and $\boldsymbol{\mu}_A$ (usually associated with the $S_0 \rightarrow S_1$ excitation), and $\boldsymbol{R}_{AB}$ is the vector, of length R_{AB}, connecting their center of mass. The simple Frenkel exciton Hamiltonian, which describes the first-excitation manifold, is

$$H_{ex} = E_{S_0 \rightarrow S_1} + \sum_{m,n} V_{mn} |m\rangle \langle n| \quad (52)$$

where the states $|n\rangle$ correspond to the n-th molecule excited while the others are in the ground state, and $E_{S_0 \rightarrow S_1}$ is the excitation energy of the molecular transition in the supramolecular aggregate, which includes an energy shift from the gas-phase excitation due to the molecular environment[12]. Diagonalizing the Hamiltonian H_{ex} for a fixed number N of molecules (or imposing periodic boundary conditions), we can reproduce the blueshift (redshift) of the excitation energy of the H-aggregates (J-aggregates). The difference in the energy shift is given by the sign of the intermolecular coupling V_{AB} in the different configurations, which is negative for J-aggregates and positive for H-aggregates. Such a simple model qualitatively accounts for the delocalized nature of the excitation and for the photoluminescence properties of these aggregates[10–12], and can be easily generalized including a different molecule (impurity) in the excitation manifold.

While this simple model is straightforward and qualitatively correct, several relevant chemical aspects are disregarded. First, we approximate the excitation manifold, including only the first excited states, while more excited states should be included to obtain a more realistic description. In addition, an *a priori* knowledge of the "environment shifted" excitation energy $E_{S_0 \rightarrow S_1}$ might not be known and could require an *ab initio* computation, for instance, via polarizable continuum models (PCM)[13–15]. The effect of molecular vibrations is also fundamental for an appropriate description of the photoluminescence properties of aggregates[12]. Moreover, the point-dipole coupling is a crude approximation and significant improvement is achieved by using extended charge distributions[16] or approaches based on quantum chemistry[17–21] which can capture the full complexity of the short-range interaction. Indeed, in Figure S13, we show the changes in the ground state density in the XY plane for an $(H_2)_2$ dimer, compared to the isolated molecules. As in the previous calculations, the two dimers have a bond length of 0.76 Å and 0.78 Å and form an H-aggregate. While at large distances, the intermolecular interaction is described by London forces and therefore decays fast with the distance R^{-6}, at smaller intermolecular distances, the intermolecular coupling has a complex form and depends on the wavefunction overlap. Similar modifications also occur in the excited state densities. In Figure S14, we show the difference between the first excited density of the H_2H'$_2$ dimer (mainly associated with the excitation of the perturbed dimer) and the sum of the *isolated* ground state density of the unperturbed H_2 and the excited state density of the impurity. As can be seen, the differences are more relevant than the ground state changes, and due to intermolecular forces, the excitation of one dimer polarized the other H_2. Analogous results are seen in Figure S15, where we plot the second excited state density of $H_2H'_2$ dimer, mainly associated with the excitation of the unperturbed dimer, and subtract the excited density of the unperturbed dimer and the ground state density of the impurity. Such density modifications include rescaling due to col-

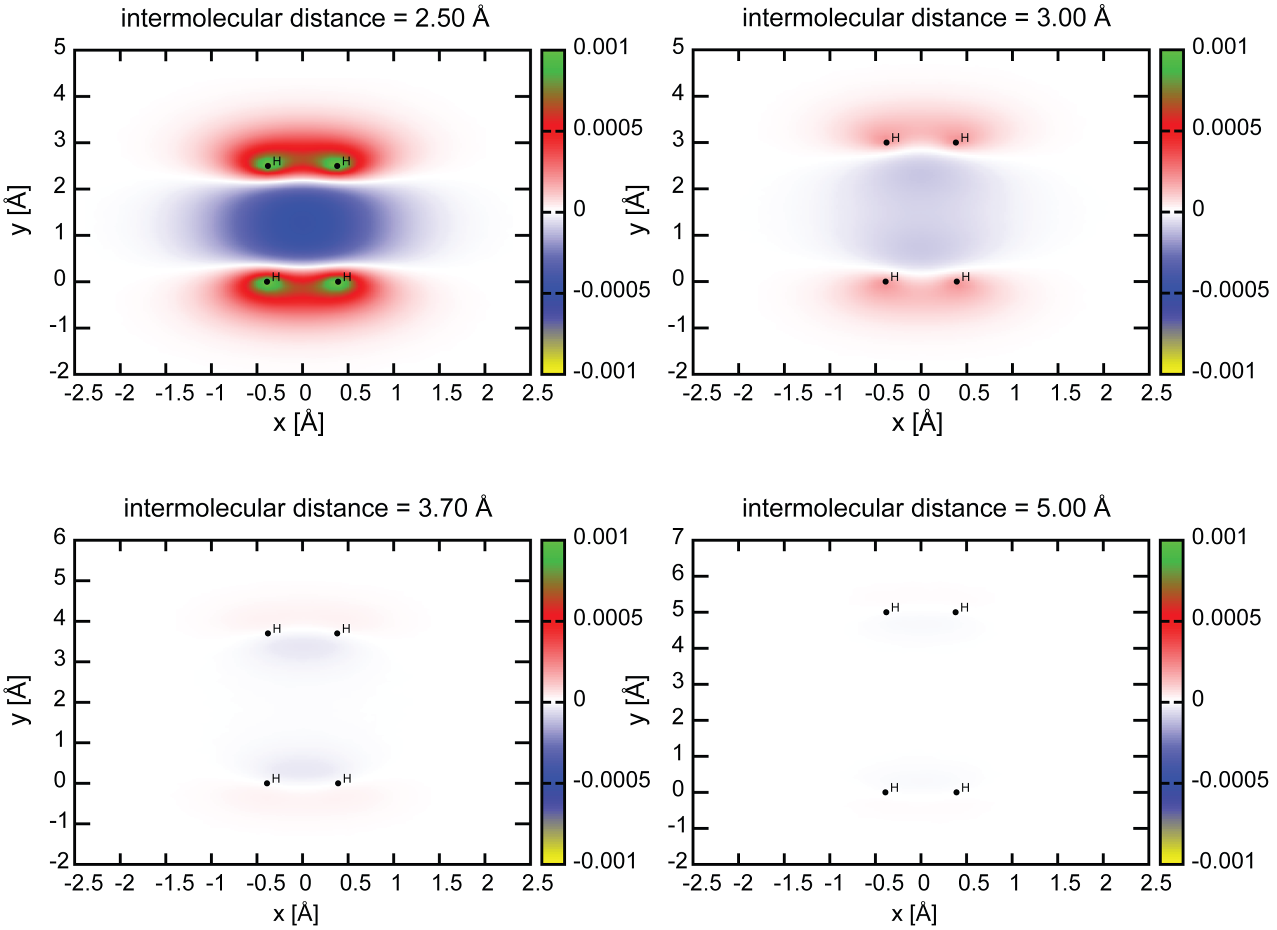

Figure S13: Variations in the ground-state densities of two parallel H_2 dimers (with bond length 0.76 Å and 0.78 Å placed on the XY plane, the perturbed dimer is placed at $y = 0$) with respect to the isolated dimers for different intermolecular distances. While the long-range interaction via the London dispersion force decays fast, there is a significant charge redistribution at small intermolecular distances. At small distances, the modifications are due to both Coulomb coupling and wavefunction overlaps, and therefore, their computation requires a proper quantum description. Similar modifications occur for the excited state densities. Density modifications also occur for larger distances for systems carrying permanent dipole moments. Moreover, specific interactions such as hydrogen bonds can significantly alter molecular densities.

lective delocalization and polarization due to intermolecular forces. While for the simple H_2 system the intermolecular forces are small, for different molecules with permanent dipole moments or carrying specific interaction sites such as hydrogen bonds or $\pi - \pi$ interactions, the density modifications can be more relevant and even influence chemical processes. These considerations apply to any supramolecular structure, including solute-solvent systems in which the solvation can significantly alter the molecular properties via hydrogen bonds, dipole-dipole, van der Waals, or other specific interactions, in addition to the effects of a different environment dielectric constant ϵ. Indeed, it is well known that solvation can modify chemical reactivity, and a suitable description of both the reactive molecule and the molecular surroundings is necessary to obtain qualitative and quantitative correct predictions. Therefore, a detailed expression of the interactions with the chemical environment is generally necessary in experimental setups such as solid matrices or solutions.

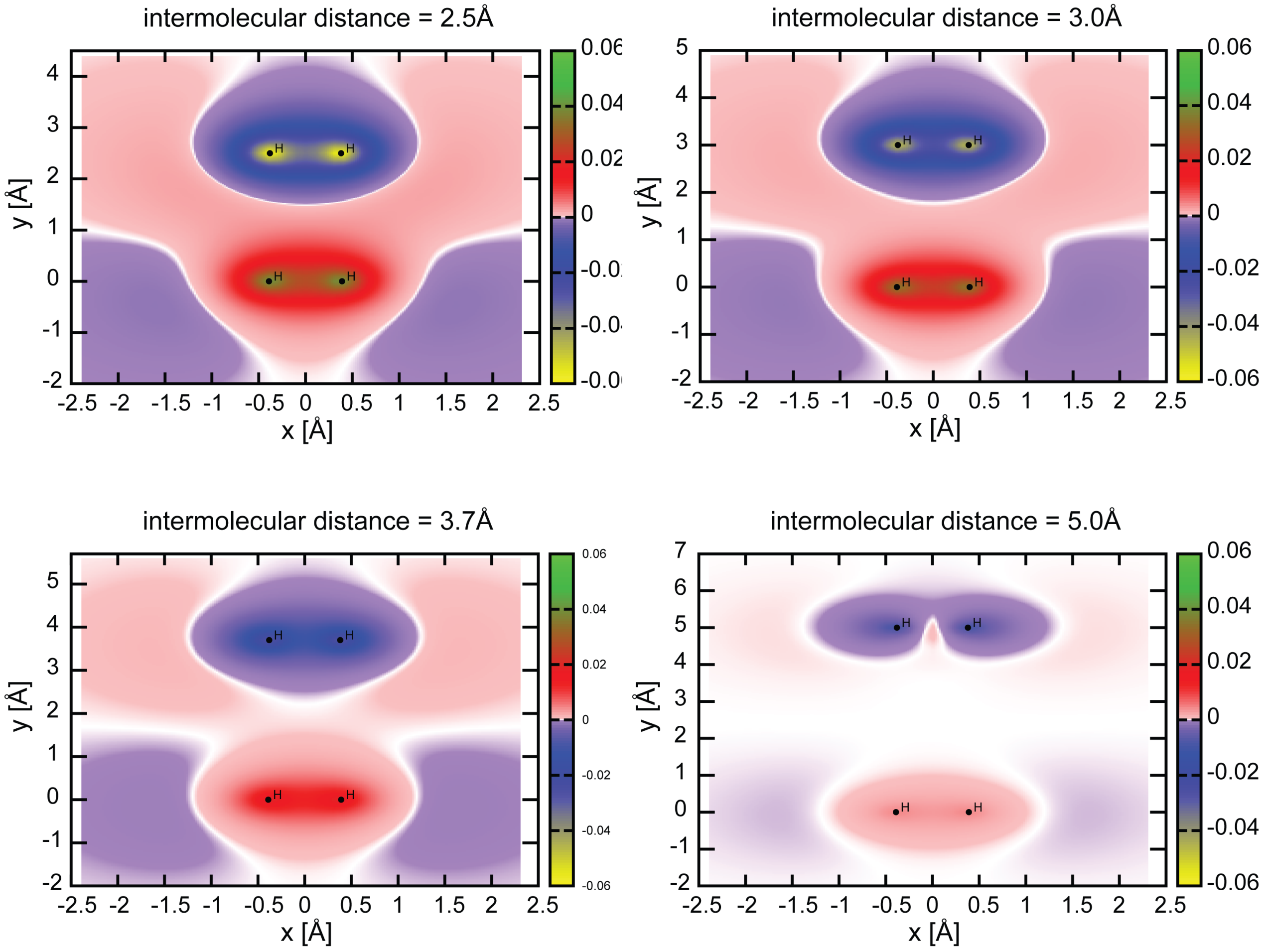

Figure S14: Variations in the first excited state (mainly associated with the impurity) density of two parallel H_2 dimers (with bond length 0.76 Å and 0.78 Å placed on the XY plane, the perturbed dimer is placed at $y = 0$) compared to the isolated ground state of the unperturbed H_2 and the excited state of the impurity, for different intermolecular distances. The differences are due to polarization and collective delocalization arising from the intermolecular coupling.

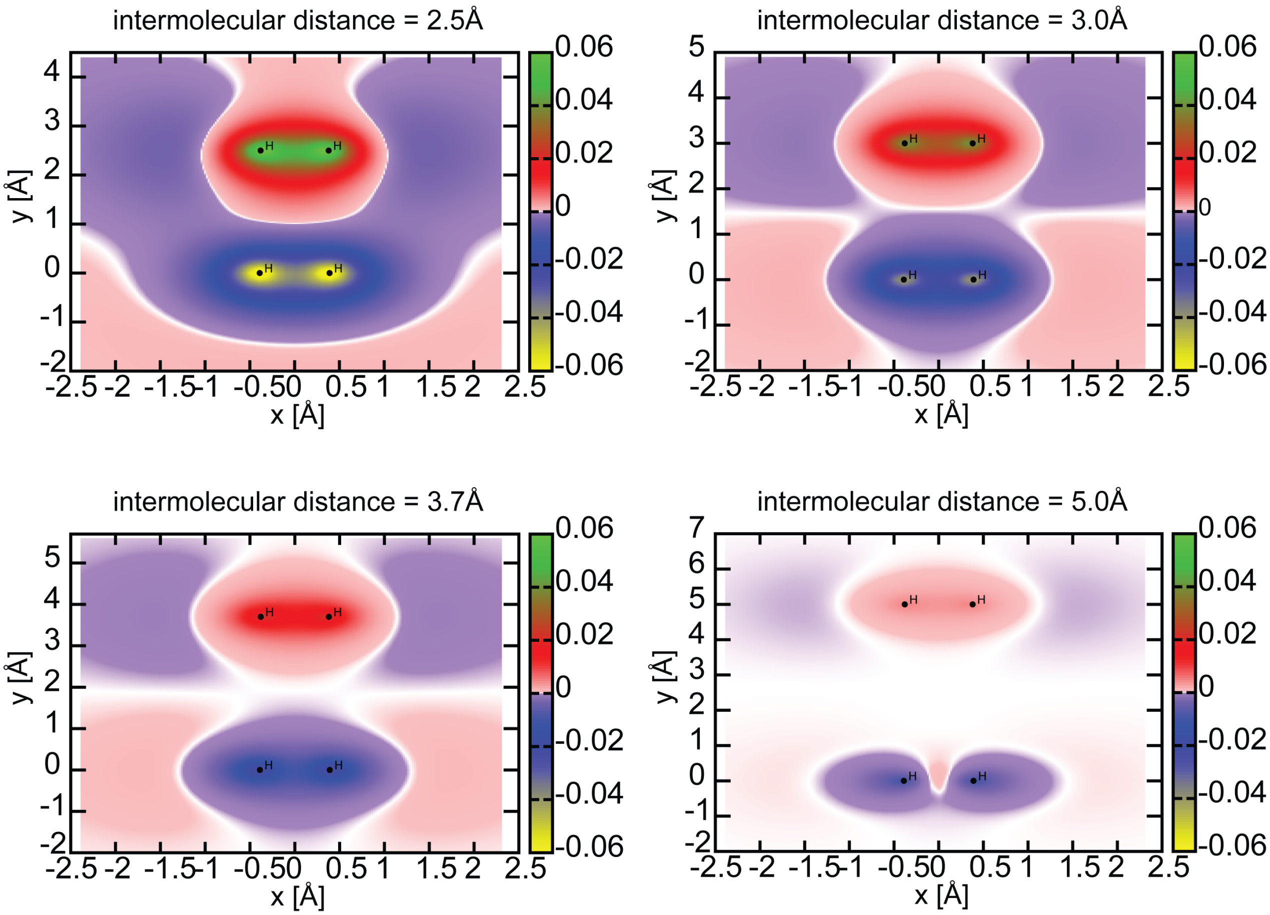

Figure S15: Variations in the second excited state (mainly associated with the unperturbed dimer) density of two parallel H_2 dimers (with bond length 0.76 Å and 0.78 Å placed on the XY plane, the perturbed dimer is placed at $y = 0$) compared to the isolated ground state of the impurity and the excited state of the unperturbed H_2, for different intermolecular distances. The differences are due to polarization and collective delocalization arising from the intermolecular coupling.

S5. H-aggregate polaritonic excitations

In Figure S16, we show the polaritonic (field-dressed) absorption spectra of the $(H_2)_{N \geq 4}$ H-aggregate (see Figure S1) for different intermolecular separations. The cavity is tuned to the undressed excitation of the (isolated) unperturbed dimers, and from the interaction between the photon field and the H-aggregate electronic excitations E1 and E2, three polaritonic branches, the lower, middle, and upper polariton emerge. As the intermolecular forces become more relevant (shorter distances), the MP gains oscillator strength while the LP loses

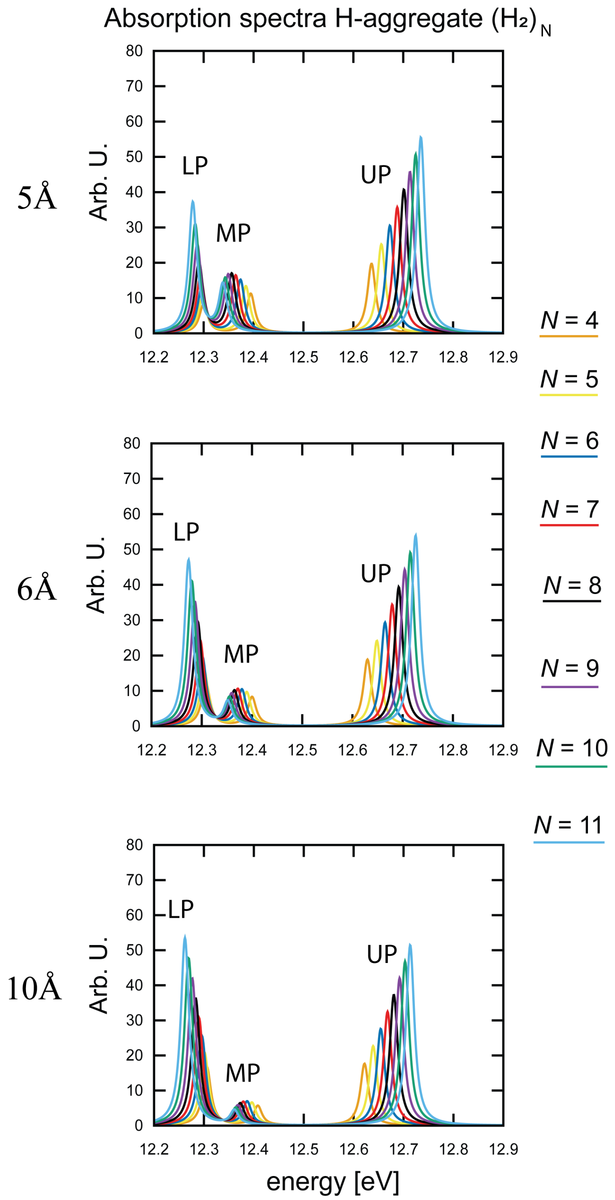

Figure S16: Polaritonic absorption spectra for an H-aggregate $(H_2)_{N \geq 4}$ for 5 Å (upper panel), 6 Å (central panel), and 10 Å (lower panel) intermolecular separation. From the interaction between the photon field and the H-aggregate electronic excitations E1 and E2, three polaritonic branches, the lower, middle, and upper polariton, emerge.

it. While the upper and lower polaritonic branches gain intensity with the chain length N, the MP shows a more involved trend. In Figure S17, we show the LP and MP photon characters computed from the transition densities for an intermolecular separation of 5 Å. The photon character of the MP steadily decreases with the chain length N, while the opposite

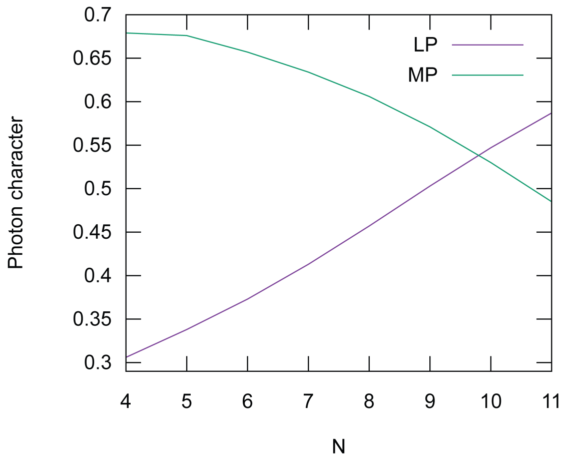

Figure S17: LP and MP photon character as a function of the chain length N for 5 Å intermolecular distance.

happens for the LP. The trends are consistent with the TC analysis of Figure S2, and analogous trends are found for other intermolecular distances. Therefore, the matter excitations E1 and E2 gain weight in the MP properties as N increases. In Figure S18, we report the energy of the LP and MP as a function of the number of dimers N in the chain for 5 Å intermolecular distance. The avoided crossing between the

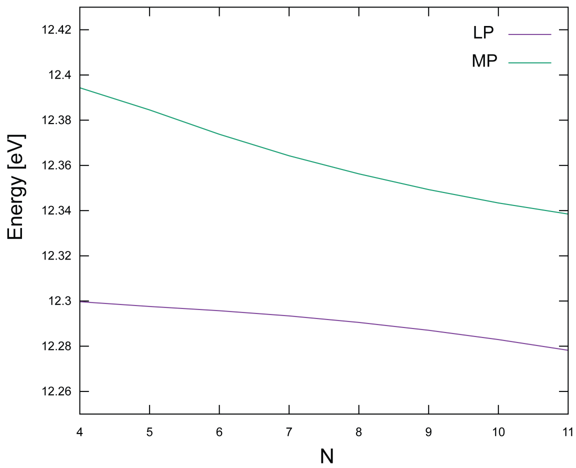

Figure S18: LP and MP energy as a function of the chain length N for 5Å intermolecular distance.

lower and upper polaritonic branches suggests relevant mixing between the E1 and E2 excitations. Analogous avoided crossings are obtained in the TC model and for different H_2 separations, but they occur at different chain lengths since the intermolecular forces modify the undressed excitations. We can then analyze the local effects of such mixing by extracting the integrated local transition densities, as explained in section and section .

In Figure S19 and Figure S20, we plot the LP left and right integrated local transition densities for the H-aggregate configuration with an intermolecular distance of 5 Å and 6 Å, respectively. The upper panels of Figure S19 are the same results as 2 in the main text. We see that the responses of A1 and A2 are different because of the intermolecular forces. In the undressed excitation E1, the local transition dipole moments are anti-aligned, while for E2, they are aligned. In the polaritonic branches, the coupling to the photon mixes E1 and E2 and tends to align the local transition moments as discussed in section for the TC model. Therefore, a competition between longitudinal and transverse fields results in a change in the alignment pattern of the transition dipole moments in Figure S19, for 5 Å intermolecular distance. For smaller intermolecular forces, at 6 Å distance, the photon effects prevail, and the transition moments are always aligned in the LP. This confirms that the pattern change results from the competition between intermolecular forces and collective strong coupling. Nevertheless, for 6 Å separation, we see significant changes in the response of A1.

In Figure S21 and Figure S22, we plot the MP left and right integrated local transition densities for the H-aggregate configuration with an intermolecular distance of 5 Å and 6 Å, respectively. Compared to the LP branch, the perturbed dimer P transition moment is anti-aligned with the rest of the chain for both distances. Notice that the MP is mainly localized on P, and the relation between the transition moments of A1 and P resembles E1. Nevertheless, the dimer A2 is now aligned to A1, contrary to E1. This is likely to be due to the mixing of E1 and E2 since, in E2, all of the dimers have a similar contribution in an aligned pattern. In contrast, the contribution of A2 in E1 is tiny and, therefore, easily overcome by E2 and the transverse field effects. Compared

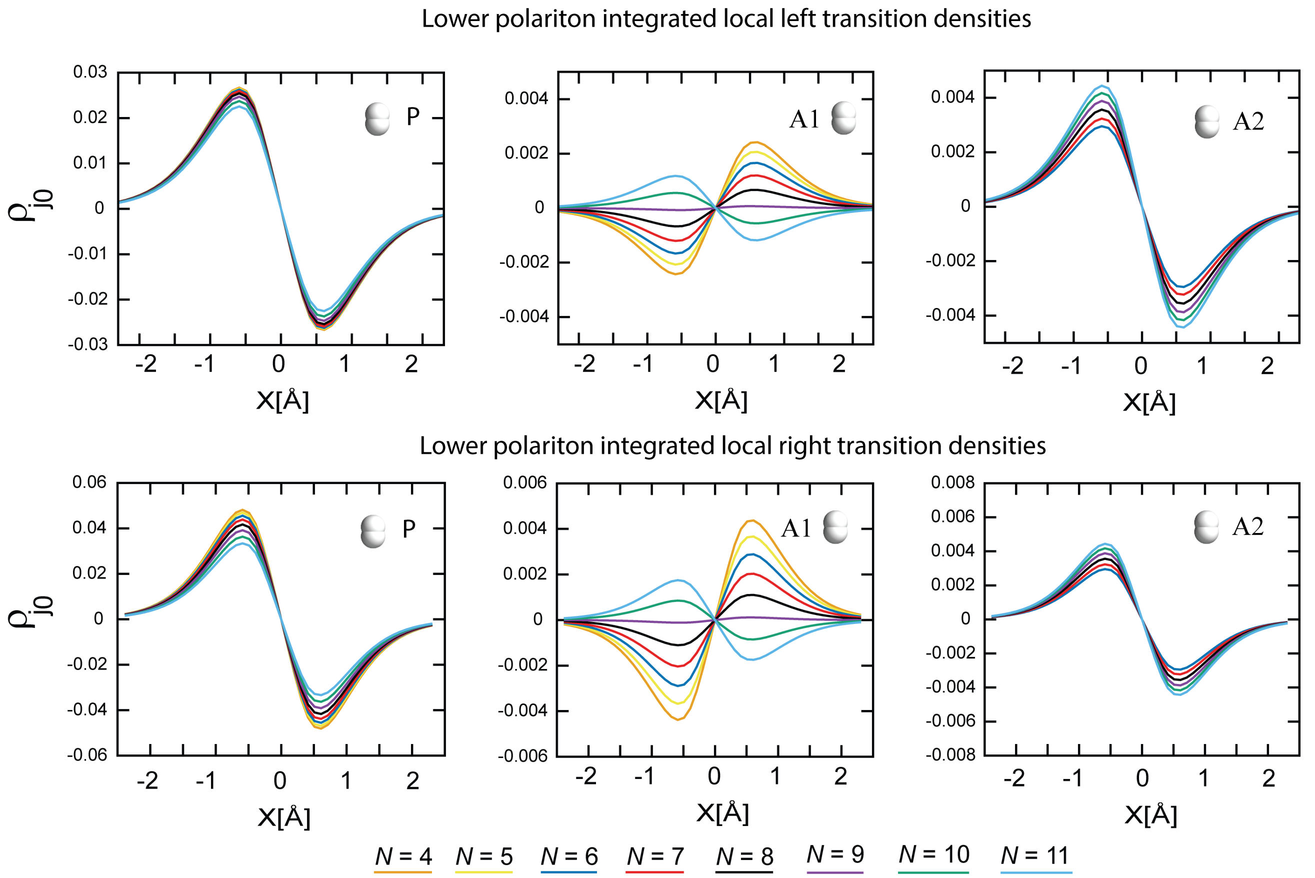

Figure S19: Local left and right transition densities for the lower polariton for an intermolecular separation of 5 Å in an H-aggregate configuration (parallel-bond arrangement, see Figure S1). As we increase the number of molecules coupled to the photon field, the local transition dipole moment of A1 changes sign. This change in the alignment patterns of the transition moments is a consequence of the competition between the longitudinal intermolecular forces and the collective coupling to the optical mode. This points out the local role of the concentration in polaritonic chemistry and hints at a modification of the solvation response under cooperative strong coupling.

to Figure S21, for 6 Å intermolecular separation, the MP is more delocalized with similar contributions from P and A1, while for 5 Å, it is more localized on P. Notice also that for 6 Å, the contribution of P increases with N, while it decreases for A1 and A2 (Figure S22). Instead, for 5 Å intermolecular separation (Figure S21), the changes with N are less evident, and the contribution of P decreases with N, behaving similarly to A1 and A2.

The cavity tuning is a fundamental experimental knob, and empirical observations show that detuning the photon disrupts the polaritonic modifications. Therefore, we investigate the effect of the photon frequency on our system. Notice that the definition of the resonance condition is nontrivial since the excitation E2 blueshifts with increasing N. We then consider four cavity frequencies for 5 Å intermolecular separation: i) tuned to the (undressed) excitation of the isolated perturbed dimer P, ii) tuned to the excitation E1 (basically independent of N), iii) tuned to E2 for the aggregate $(H_2)_7$, and iv) completely detuned at higher energies. Resonance with E1 or P provides a model for the photon tuned with the impurity excitation. For the resonance with E2, we expect effects similar to the above results. Finally, when the photon is completely detuned, we expect no modifications compared to the undressed case. In Figure S23, we show the LP local left transition densities of P, A1, and A2 (H-aggregate, intermolecular separation of 5 Å). When the

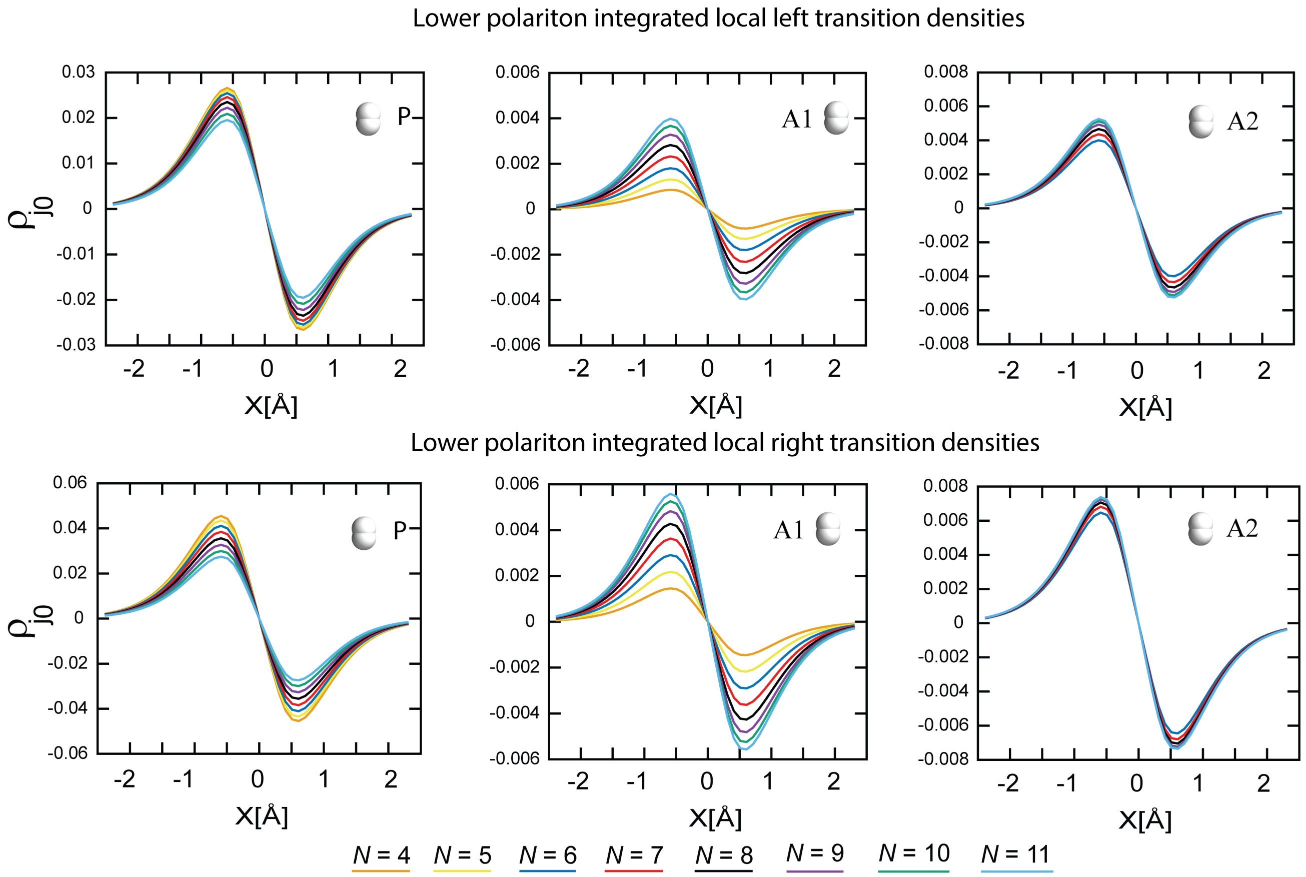

Figure S20: Local left and right transition densities for the lower polariton for an intermolecular separation of 6 Å in an H-aggregate configuration (parallel-bond arrangement, see Figure S1). Compared to Figure S19, the local transition dipole moments are already aligned even for short chains, so there is no sign-flip in the transition moment of A1. Nevertheless, we see that as N increases, the response of A1 becomes more and more relevant and more similar to A2. This behavior is still a consequence of the intermolecular forces between the dimers, but the collective photon effects here prevail since, at larger distances, the longitudinal coupling is smaller.

photon is tuned to the impurity (either resonant with the undressed excitation of the isolated P molecule or resonant to the excitation E1, first two columns), we observe small local changes that are more pronounced for P. The transition moments are aligned as in E2, and the changes in A1 and A2 are modest. When the cavity is tuned to E2 of $(H_2)_7$ (third column), we see significant changes in the local transition moment of A1, which resemble the results for the photon tuned to the undressed excitation of E1. Finally, when the photon is completely detuned to the excitation (rightmost panels of Figure S23), there is no local change in the transition densities, and the LP shows the same pattern as E1. Since the cavity has a much larger energy than the matter excitation, the hybridization is almost negligible, and the states are effectively the same as the undressed excitations. However, the collective strong coupling for such N is not enough to flip the sign of the A1 transition densities, and longer chains would be necessary. We then recover the resonance condition as a fundamental knob to observe local modifications of molecular properties under cooperative strong coupling. In our model, the cavity frequency determines the efficiency in the hybridization of E1, E2, and the cavity photon, therefore determining the changes in the alignment pattern of the transition dipoles. In Figure S24, we show the LP and MP energies as a function of N for the cavity resonant to A1 (left), E1 (center), and E2 in $(H_2)_7$(right). In all three cases, the en-

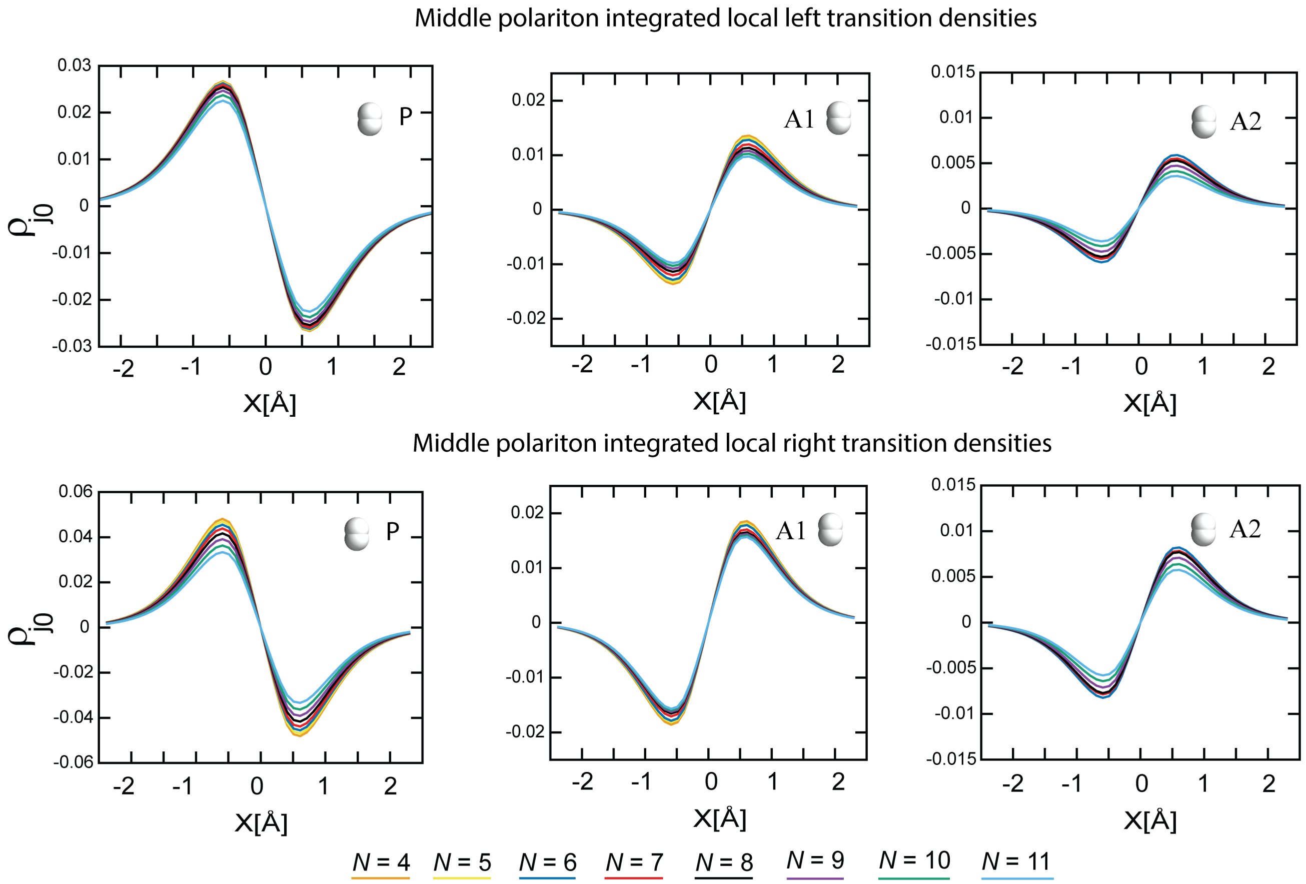

Figure S21: Local left and right transition densities for the middle polariton for an intermolecular separation of 5 Å in an H-aggregate configuration (parallel-bond arrangement, see Figure S1). The P and A1 local transition dipole moments are antialigned as in E1, but, on the opposite, A2 is aligned to A1. The alignment A1-A2 is due to photon field effects from mixing with E2 and the favored alignment of the dimers due to the photon field. Since the contribution of A2 to E1 is small, it is easily overcome by the transverse field effects. The MP is mainly localized on P but also shows significant contributions from A1 and A2.

ergies display an avoided crossing between the polaritonic branches. Still, when the photon is tuned to the undressed excitation of the isolated A1 (or to E2, first and last columns), the avoided crossing is more pronounced compared to the tuning to E1 (central column). This suggests a more efficient mixing of the undressed excitation and the one-photon state, resulting in the observed local modifications. On the other hand, when the cavity is tuned to E1, the avoided crossing is less pronounced and already overcome for small chain lengths, suggesting a less efficient mixing in the polaritonic branches. This is consistent with the changes in the patterns of the local transition moments.

Finally, we investigate the spectral effect of decreasing the coupling strength while increasing the number of dimers to keep the ratio N/V_{el} fixed, where V_{el} is the quantization volume (connected to the light-matter couplings strength $\lambda = \sqrt{\frac{1}{\varepsilon_0 V}}$). In Figure S25, we show the absorption spectra (LP and MP branches) for the 5 Å intermolecular separation H-aggregate. The left panel is the same as Figure S16, while the right panel is for a system with twice as much dimers and coupling strength rescaled by $1/\sqrt{2}$ to maintain the N/V_{el} ratio. The LP and MP excitation energies are the same in the two realizations, which is consistent with the TC predictions in the thermodynamic limit. At the same time, the peaks are more intense due to the larger number of oscillators contributing to the exci-

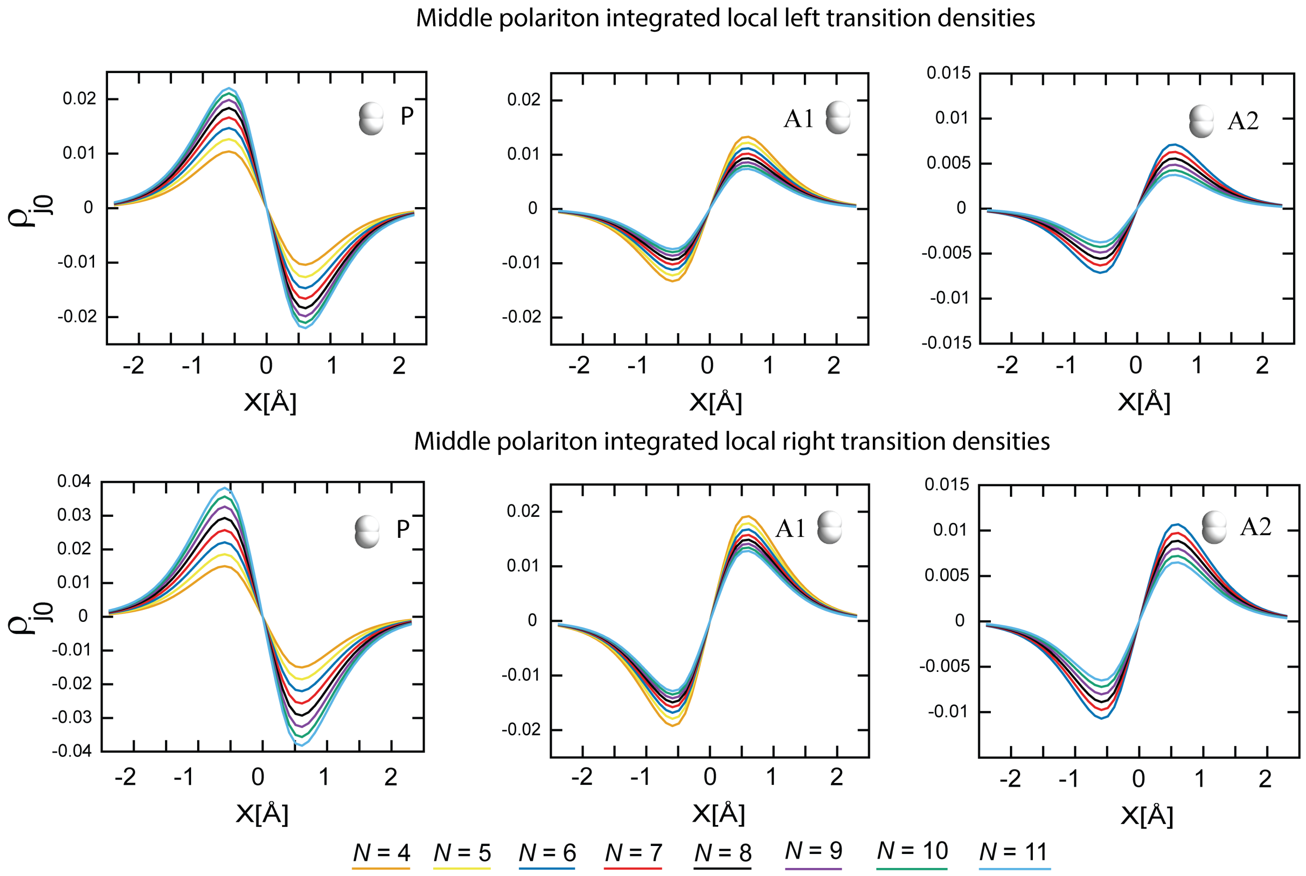

Figure S22: Local left and right transition densities for the middle polariton for an intermolecular separation of 6 Å in an H-aggregate configuration (parallel-bond arrangement, see Figure S1). The P and A1 local transition dipole moments are antialigned as in E1, but, on the opposite, A2 is aligned to A1. The alignment A1-A2 is due to photon field effects from mixing with E2 and the favored alignment of the dimers due to the photon field. Since the contribution of A2 to E1 is small, it is easily overcome by the transverse field effects. The pattern is the same as Figure S21, but the MP is more delocalized over different dimers, with more similar contributions from A1 and P.

tations.

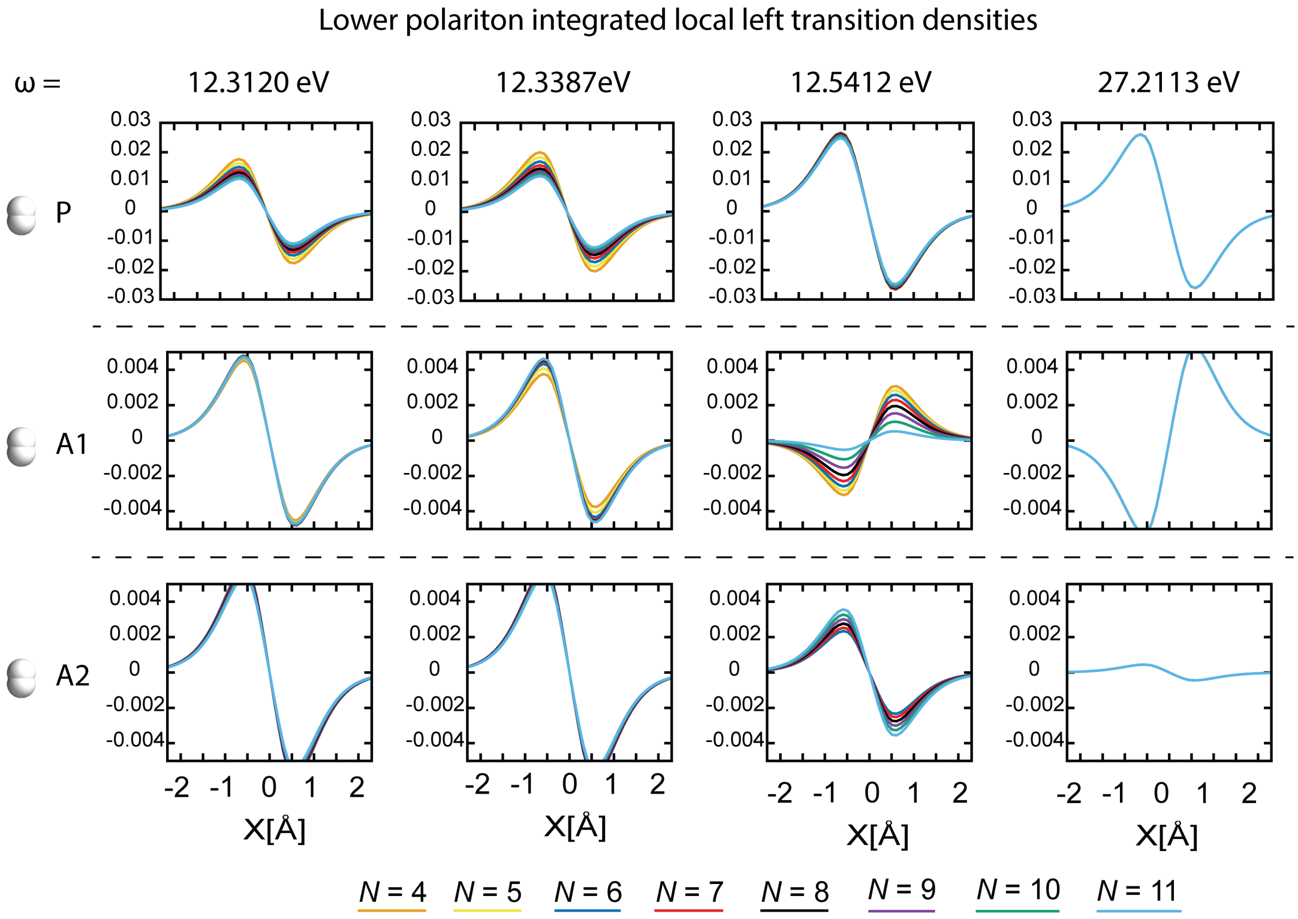

Figure S23: LP integrated local transition densities for P, A1 and A2 for different photon frequencies.

When the photon is resonant with the impurity (either E1 or the undressed P excitation, first two columns), there are some modifications in the P transition density and smaller changes in A1 and A2. The excitations resemble E2, and no change in the alignment pattern of the transition moments is predicted.

When the excitation is resonant with E2 (third column), we observe a trend similar to the cavity tuned to the undressed excitation of A1. Still, the collective strong coupling is insufficient to achieve a flip in the A1 transition dipole, and longer chains are necessary.

Finally, when the photon is completely detuned (rightmost column), there is negligible mixing between E1, E2, and the electromagnetic states, and therefore the excitations are analogous to the undressed ones. Therefore, we recover the resonant condition as a fundamental knob to observe local modifications of collective strong coupling since it determines the efficiency in the mixing of the undressed states.

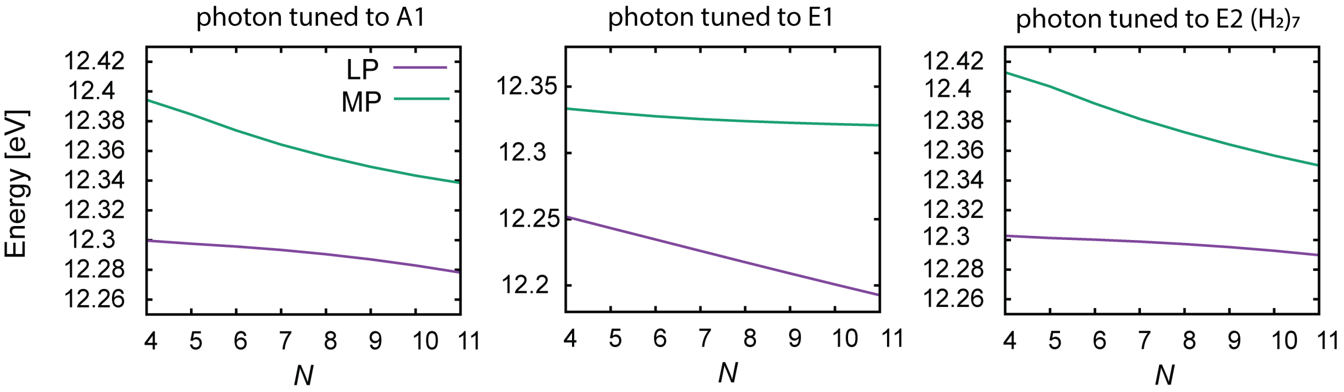

Figure S24: LP and MP energies for three different photon frequencies as a function of the chain length. The avoided crossings between the polaritonic branches indicate a relevant mixing between the molecular states. In resonance with the perturbed excitation (central panel), the avoided crossing is less pronounced and already overcome for small N, suggesting a less efficient interaction. This result is consistent with the less pronounced local transition density modifications.

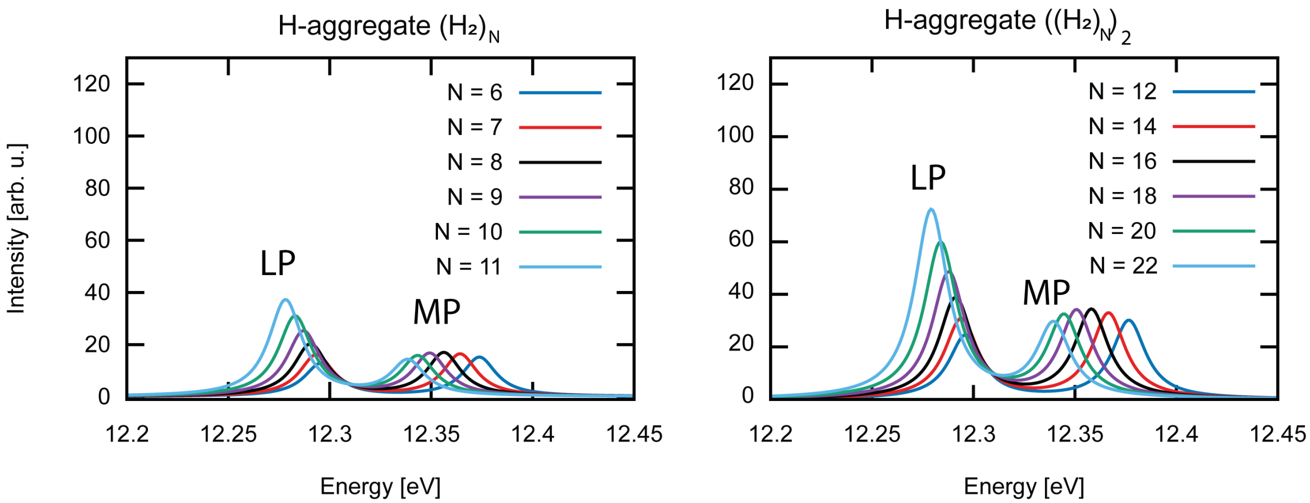

Figure S25: Left, polaritonic absorption spectra for a 5 Å intermolecular separation H-aggregate $(H_2)_{N\geq 6}$ (same as Figure S16). Right, spectra for a system with double dimension compared to left, $(H_2)_{N\geq 12}$, and coupling strength rescaled by $1/\sqrt{2}$. The LP and MP excitation energies are the same in these two realizations, consistent with the TC predictions in the thermodynamic limit.

A. TC-Kasha model for the molecular polaritons

The sign flip in the local transition density of A1 in Figure S19 can be modeled using a simplified picture that merges the TC and the Frenkel-Kasha models. Following the same procedure as the TC model, we consider the first excited state manifold of the N hydrogen dimers, each with the same excitation energy as the isolated molecules. Each H_2 is then coupled to the photon field via its isolated transition dipole moment as in Equation 34. Following the same assumptions as in the Kasha model in Equation 52, we introduce the intermolecular interactions via the transition dipole-dipole coupling of Equation 51. For simplicity, we couple only the nearest dimers, and the Hamiltonian matrix for the TC-Kasha model then reads

$$\begin{pmatrix} \omega & \tilde{g}_0{}' & \tilde{g}_0 & \tilde{g}_0 & \tilde{g}_0 & \cdots \\ \tilde{g}_0{}' & \omega' & V' & 0 & 0 & \cdots \\ \tilde{g}_0 & V' & \omega & V & 0 & \cdots \\ \tilde{g}_0 & 0 & V & \omega & \ddots & \ddots \\ \vdots & \vdots & \ddots & \ddots & \ddots & \ddots \end{pmatrix}. \tag{53}$$

Diagonalizing the Hamiltonian in Equation 53, we obtain the eigenvalues and eigenvectors corresponding to 3 polaritonic branches and N-2 (quasi) dark states. Compared to the *ab initio* simulations, which are limited by the steep computational costs of the CC methods, it is possible to perform simulations with large N. In Figure S26, we show the coefficients of P (left), A1 (center), and A2 (right) for the LP from Equation 53 for coupling strengths $\lambda = 0.002$ a.u., 0.003 a.u., 0.004 a.u. and 0.005 a.u. as a function of the intermolecular separation and the chain length N. The qualitative behavior and the order of magnitude of the coefficients are the same in all cases and agree with the *ab initio* results in Figure S19. The effects of rescaling the coupling strength λ for a fixed intermolecular separation by a factor α shifts the avoided crossing from length N roughly to $N' \sim N/\alpha^2$, which is consistent to the thermodynamic limit requirement $N_p/V_{el} =$ const. for $N_p, V_{el} \to \infty$, where V_{el} is the quantization volume $\lambda \propto \sqrt{V_{el}^{-1}}$. Therefore, this local sign flip involving A1, which stems from the competition between longitudinal and transverse fields, is persistent even in the thermodynamic limit. In Figure S27, we show the coefficients of P (left), A1 (center), and A2 (right) for the LP obtained from the matrix Equation 53 where the bare coupling strength is $\lambda_0 = 0.0$ a.u., 0.01 a.u., 0.0125 a.u. and 0.015 a.u. further rescaled by $1/\sqrt{N}$, that is, $\lambda = \lambda_0/\sqrt{N}$. In agreement with the *ab initio* simulations, outside the cavity (first row of Figure S27), the coefficients of P, A1, and A2 display alternate signs due to the intermolecular forces, and the contribution of A1 and A2 to the lowest excitation, mainly associated with P, increases for shorter distances. When the photon coupling is switched on, the coefficient of A1 still displays a sign flip, and the order of magnitude of the coefficients is the same in the three simulations. The position at which such a sign flip occurs depends on λ_0, the intermolecular separation, the chain length N, and the excitation energy of the impurity. Therefore, the described effects are resistant to the thermodynamic limit for a single impurity P.

The simplified TC-Kasha model successfully reproduces the spectral behavior of the QED-CC results. However, in this framework, the transition and excited state densities can be reconstructed from Equation 53 only from the *isolated* H_2 densities, which are available as an input for the model. Moreover, the ground state is assumed to be unchanged. For the H_2 system under consideration, the modifications of the ground and excited state densities are non-negligible for intermolecular distances below 5 Å, as can be seen from Figure S13, S14, and S15. Therefore, the mixing and rescaling of a few isolated densities coming from the eigenvectors of Equation 53 could be unsuited for describing such local modifications, which can be responsible for alterations of chemical processes such as molecular reactivity. On the other hand, QED-CC includes all the quantum chemistry effects due to wavefunction overlap and Coulomb forces. Therefore, when the effect of the solvent is non-perturbative, although

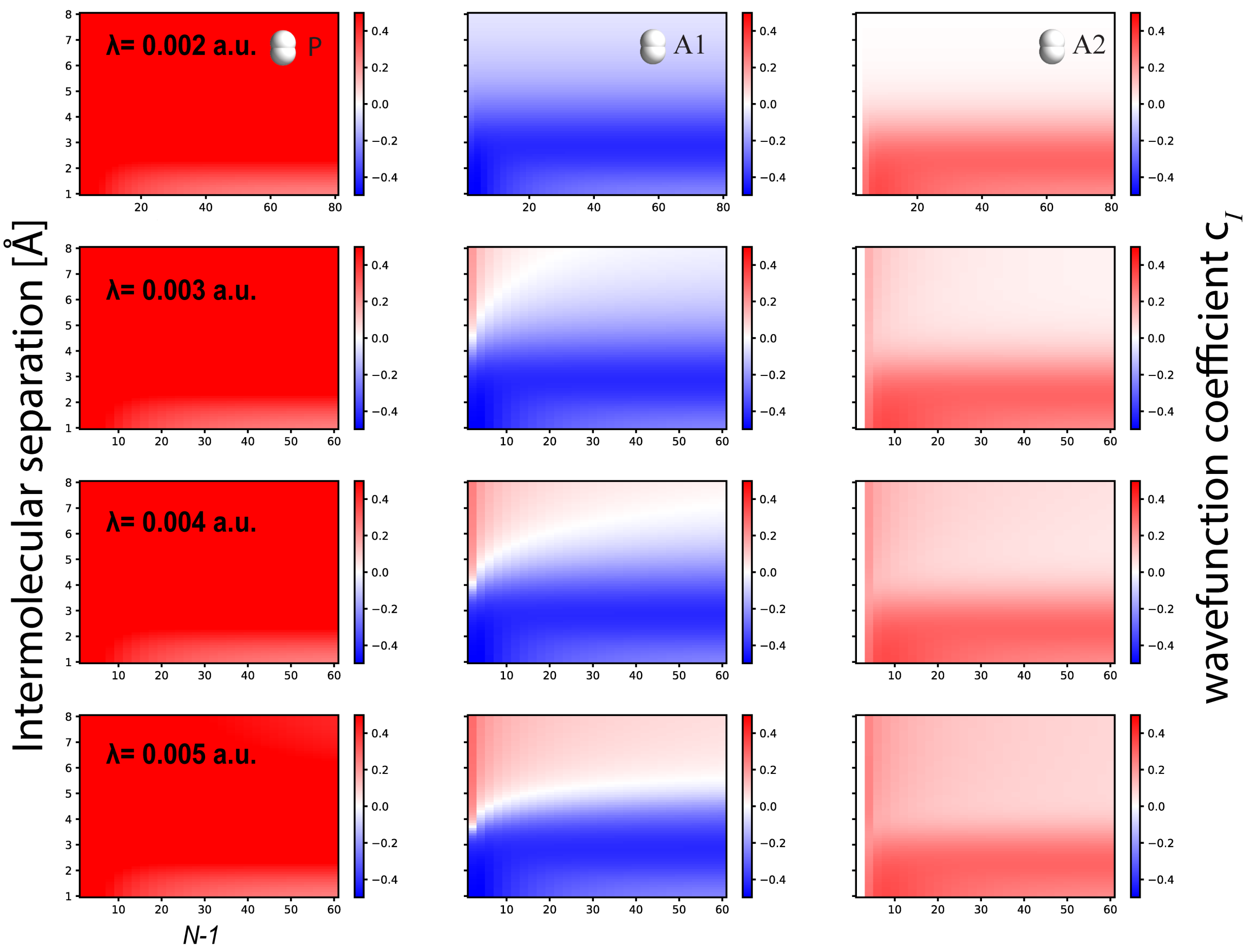

Figure S26: Lp wave function coefficients c of P, A1, and A2 (left to right) with $\lambda = 0.002$ a.u., 0.003 a.u., 0.004 a.u. and 0.005 a.u. from top to bottom.

a simplified optical model can be used to reproduce the spectral polaritonic properties, an *ab initio* description of the solute-solvent will be necessary for a detailed investigation of the single-molecule (local) chemistry changes observed in several polaritonic chemistry experiments. While the current discussion has focused on electronic strong coupling, the same considerations hold for vibrational excitations. Quantum chemistry is then necessary to model the potential energy surfaces on which the nuclei move. An analogous interplay between longitudinal and transverse field effects is also expected in the framework of vibrational strong coupling. In particular, solute-solvent interactions, such as hydrogen bonds, will significantly modify the molecular vibrations. Moreover, the light-matter strong coupling can modify intermolecular forces, and it is suggested in particular that van der Waals forces can be particularly affected[22–25]. While a few-level CI model might not capture such electron-photon correlation effects, they are naturally included in *ab initio* QED.

So far, we have focused on a single perturbed dimer P. However, the TC-Kasha model also allows us to study N_{rep} replicas of the H_2 chain. In Figure S28, we show the TC-Kasha eigenvector coefficients for P, A1, and A2 (from left to right) for coupling strengths $\lambda_0 = 0.004$ a.u. (first three panes) and $\lambda_0 = 0.002$ a.u. (last three panels) for number of chain replicas N_{rep} = 1, 4 and 16 (from top to bottom for each λ_0). The light-matter coupling strength is then rescaled by $1/\sqrt{N_{rep}}$, that is, $\lambda = \lambda_0/\sqrt{N_{rep}}$. For each λ_0, the results show the same qualita-

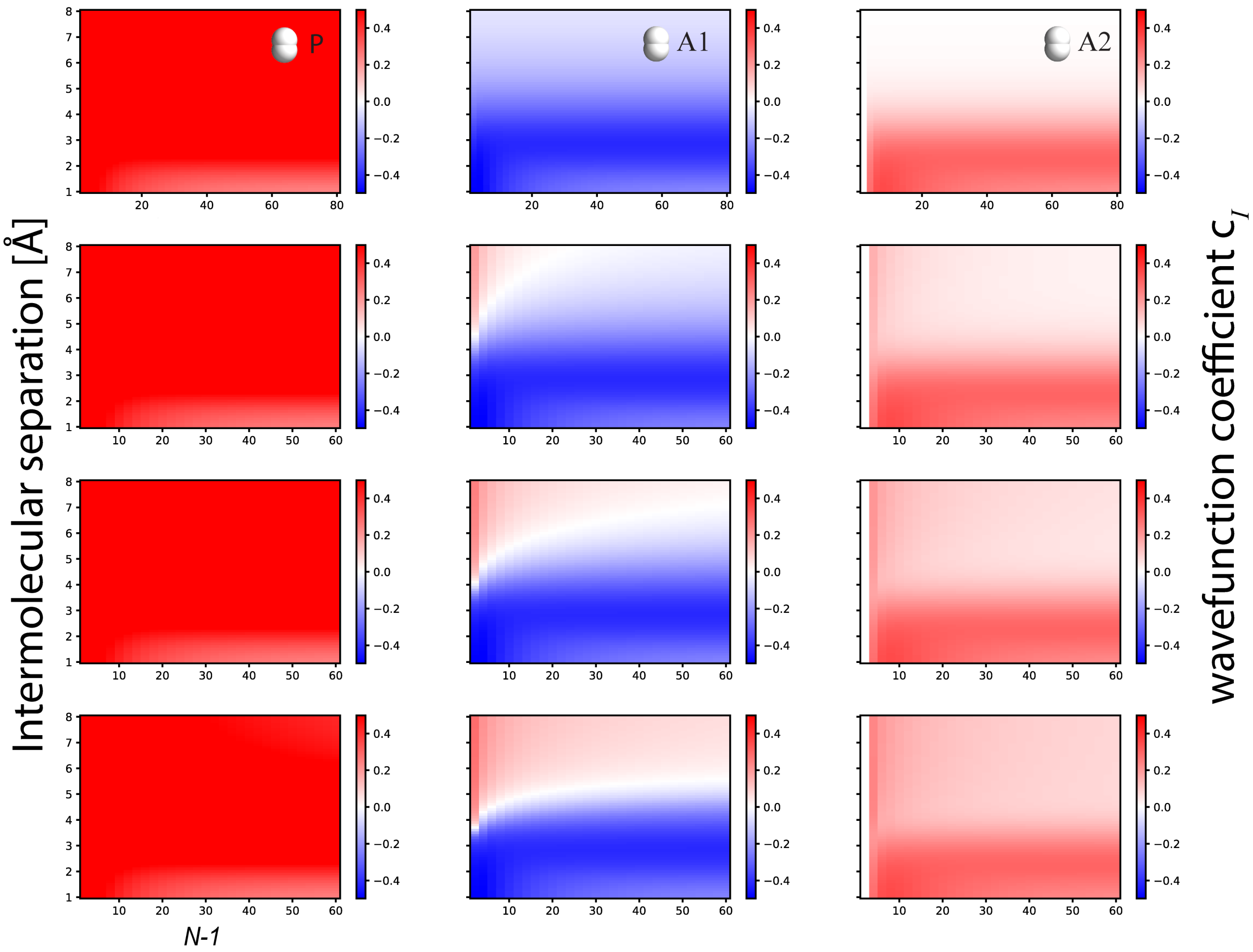

Figure S27: Lp wave function coefficients c of P, A1, and A2 (left to right) without (top) cavity, where the cavity strength is scaled as $\lambda = \lambda_0/\sqrt{N}$ with λ_0 = 0.0 a.u., 0.01 a.u., 0.0125 a.u. and 0.015 a.u.

tive behavior for every N_{rep}. We still predict a sign flip in the A1 coefficients, which means the qualitative behavior resists the thermodynamic limit. Nevertheless, notice that the coefficients in this case decrease of a factor $(\sqrt{N_{rep}})^{-1}$ and the coefficients will asymptotically approach zero. Similar results are obtained from the *ab initio* simulations, although the computational costs limit the number of replicas we can study using QED-CC. In Figure S29, we show the LP integrated local transition density matrix for a chain containing 2 perturbed dimers for 5Å intermolecular distance, with coupling strength rescaled by the factor $1/\sqrt{2}$. The two perturbed dimers are separated by 6 unperturbed H_2 so that their direct influence is negligible. For comparison, the transition densities for a single perturbed dimer and unscaled light-matter coupling strength are shown in dotted lines. In agreement with the TC-Kasha model, the qualitative sign-inversion behavior of A1 is reproduced, the position N at which the sign-flip occurs is also the same, and the contribution of each dimer decreases by roughly the same scaling factor as the coupling strength. Nevertheless, we point out that the number of replicas is smaller than the number of coupled dimers, that is, $N_{tot} = N \times N_{rep}$. Therefore, the presented results show a slower decay in the thermodynamic limit as $1/N_{rep}$ instead of $1/N_{tot}$.

In Figure S30, we plot the coefficients for P, A1, and A2 (left, center, and right panels, respectively) for up to 80 unperturbed dimers

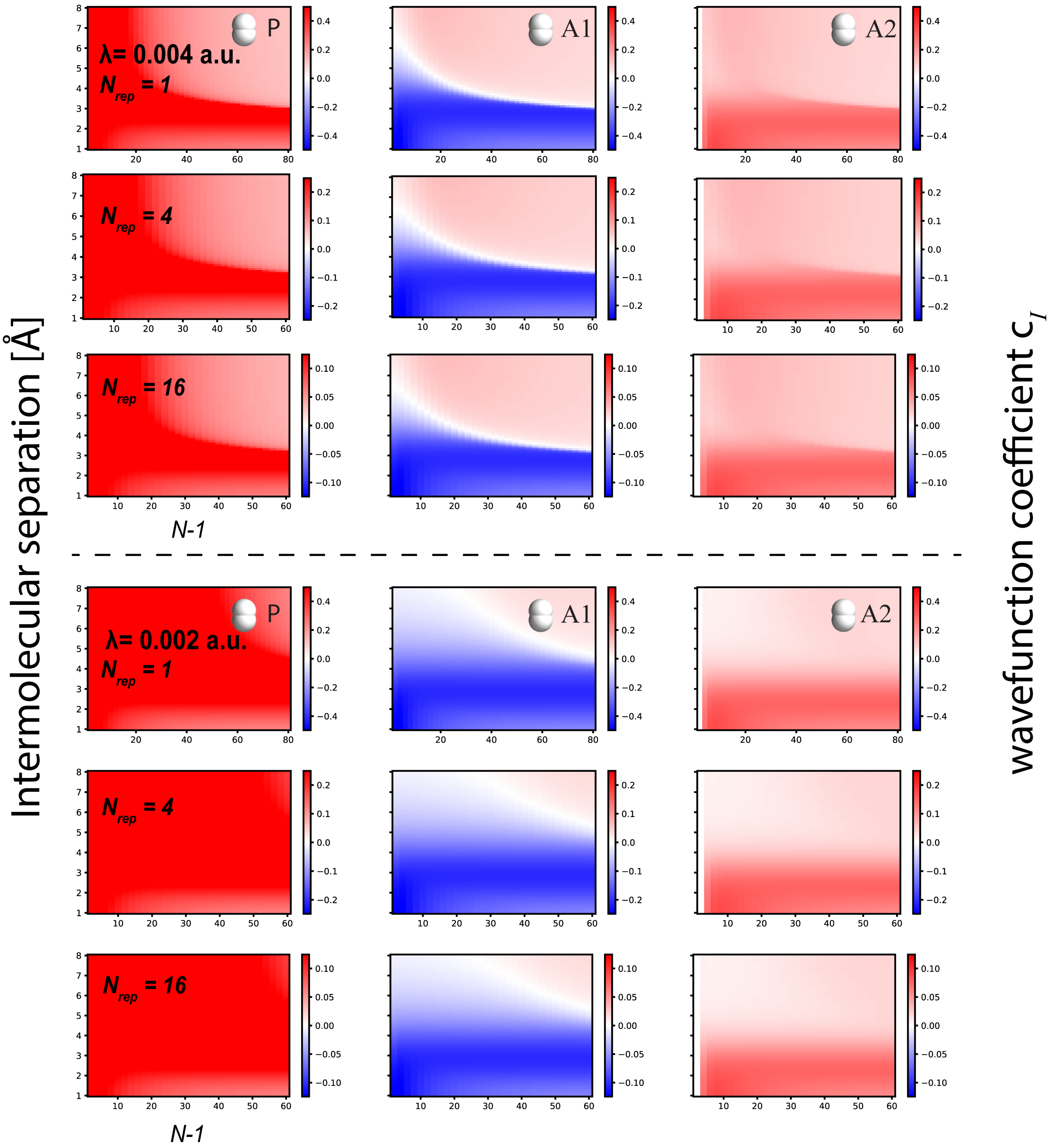

Figure S28: LP wave function coefficients c of P, A1, and A2 (left to right). The first three panels refer to a bare coupling strength of $\lambda_0 = 0.004$ a.u., while for the last three $\lambda_0 = 0.002$ a.u.. For each λ_0, the calculations are repeated for 1, 4, and 16 duplicates of the chain (from top to bottom), and the coupling strength is rescaled by $1/\sqrt{N_{rep}}$ i.e. $\lambda = \frac{\lambda_0}{\sqrt{N_{rep}}}$.

(upper panels) for a bare light-matter coupling strength λ of 0.01 a.u. and different intermolecular distances. In the second and third rows of Figure S30, we then duplicate the system, adding 5 (second row) and 10 (last row) identical replicas and consequently rescaling the fundamental coupling strength $\lambda/\sqrt{N \times N_{rep}}$. The figures show the same qualitative sign flip

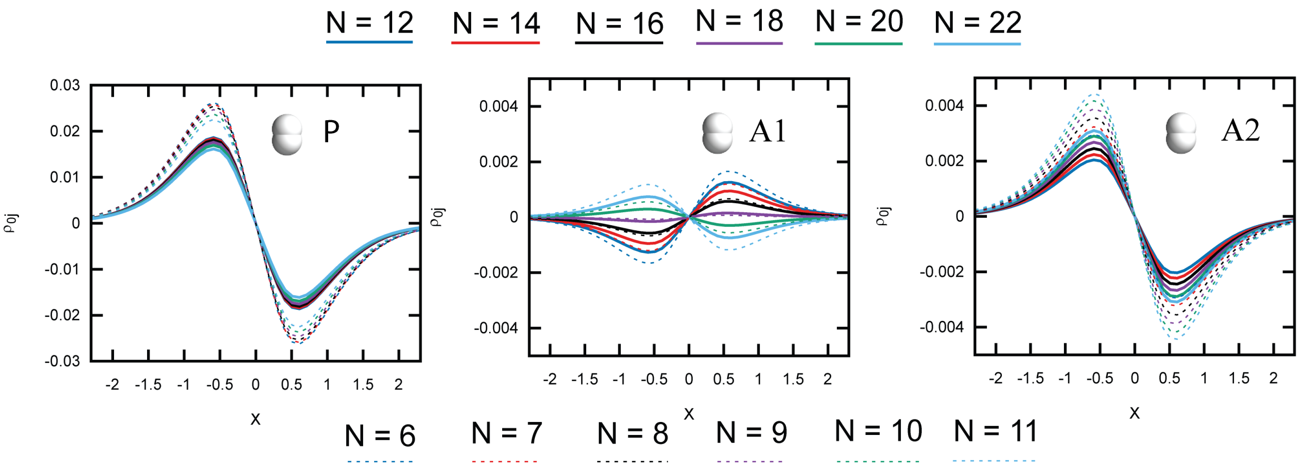

Figure S29: The solid lines show the local transition densities for P, A1, and A2 for a chain containing two perturbed dimers and intermolecular separation of 5Å. Compared to Fig.Figure S19, the coupling strength has been rescaled by $(\sqrt{2})^{-1}$, and the two perturbed H_2 are separated by 6 dimers so that their mutual influence is negligible. The dotted lines show for reference the same results as in Figure S19 for a chain with a single perturbed dimer. The results for these two realizations are qualitatively the same and display the same order of magnitude. The differences are likely due to different intermolecular forces arising from the different number of dimers, dipole self-energy, and electron-photon correlation.

behavior as a function of N and the intermolecular separation. This confirms again that such a local change, stemming from the interplay between longitudinal (intermolecular forces) and transverse (photon) fields, is resistant to the thermodynamic limit.

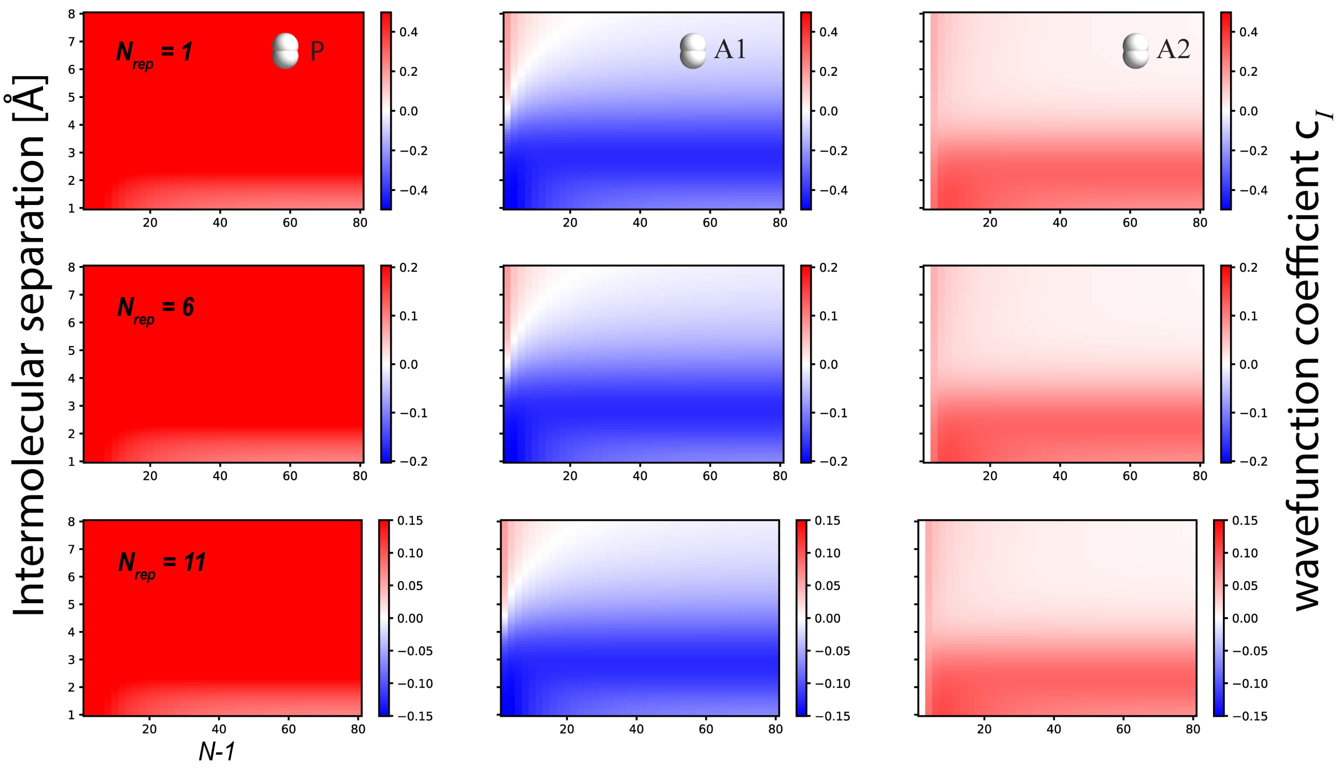

Figure S30: Lp wave function coefficients c of P, A1, and A2 (left to right) for 1, 6, and 11 duplicates (from the top down to the bottom) of the H-aggregate system with up to 40 bare molecules. The cavity strength is scaled as $\lambda = 0.01/\sqrt{N_{tot}}$ to investigate the thermodynamic limit $N, V_{el} \rightarrow \infty,\ N/V_{el} = c$.

Using a simplified TC-Kasha model also allows us to study the effect of disorder in the system. To this end, we consider P and its first solvation shell (A1) to be fixed while introducing disorder in the remaining dimers. Therefore, we model only long-range disorder while short-range disorder (in the vicinity of P) is suppressed for simplicity. A Gaussian distribution of the excitation energies, centered at the unperturbed dimers' excitation energies, introduces an energy broadening. Such an energy broadening can be thought of as a distribution of bond lengths in the dimers caused by thermal fluctuations. Moreover, the orientational disorder is introduced by assuming the dimers can form an angle $\theta_k \in [0, \pi/2]$ with the X axis (k labels the different disordered dimers). At the same time, for simplicity, we keep them orthogonal to the Y axis. The restriction $\theta_k \in [0, \pi/2]$ (instead of $\theta_k \in [0, \pi]$) ensures that the local transition dipole moments are still aligned in the same direction to avoid fictitious flipping of the wavefunction coefficients (see Equation 44). We also assume all the dimers to be kept at a fixed distance. Therefore, the Hamiltonian to be diagonalized here reads

$$\begin{pmatrix} \omega & \tilde{g}_0{}' & \tilde{g}_0 & \tilde{g}_0(3) & \tilde{g}_0(4) & \cdots \\ \tilde{g}_0{}' & \omega' & V' & 0 & 0 & \cdots \\ \tilde{g}_0 & V' & \omega & V(2,3) & 0 & \cdots \\ \tilde{g}_0(3) & 0 & V(2,3) & \omega(3) & V(3,4) & \ddots \\ \tilde{g}_0(4) & 0 & 0 & V(3,4) & \omega(4) & \ddots \\ \vdots & \vdots & \ddots & \ddots & \ddots & \ddots \end{pmatrix}. \tag{54}$$

In Equation 54, the dimer labeled 1 is P, A1 is labeled 2, and the remaining N-2 dimers have energy disorder embedded in the excitation energy on the diagonal $\omega(k)$, and orientational disorder which causes a decrease of the light-matter coupling strength compared to the ordered case $\tilde{g}_0(k) = \tilde{g}_0 \cos(\theta_k)$. Moreover, the orientational disorder also affects the dipole-dipole couplings $V(m,n)$, as seen in Equation 51. Refinement of such simplified disorder can be obtained by relaxing the rotation constraints and permitting a displacement of the H_2. In Figure S31, we show the LP coefficients obtained from the matrix in Equation 54 for P, A1, and A2 (from left to right) for coupling strengths $\lambda = 0.002\,\text{a.u.}$, $0.003\,\text{a.u.}$ and $0.004\,\text{a.u.}$ (from the top down to the bottom). These results are analogous to Figure S26. The order of magnitude of the wave function coefficients and the qualitative sign flip of the A1 coefficients are again obtained, although for a larger number of dimers N. Indeed, the collective coupling of the system is decreased because of disorder since the coupling to the cavity, compared to the perfectly ordered case, is scaled by $\cos\theta_k$ for each dimer. Therefore, a larger number of coupled molecules is generally necessary to reach the avoided crossing between the LP and the MP. Nevertheless, the fact that this effect resists the introduction of long-range disorder proves that it stems from the interplay between two distinct molecular environments: the local chemical environment (solvation shell) and the collective optical environment (polaritonic dressing). It also confirms the local nature of such changes. Therefore, we expect the presented results always to be relevant for systems characterized by short-range order, such as solute-solvent systems or aggregates.

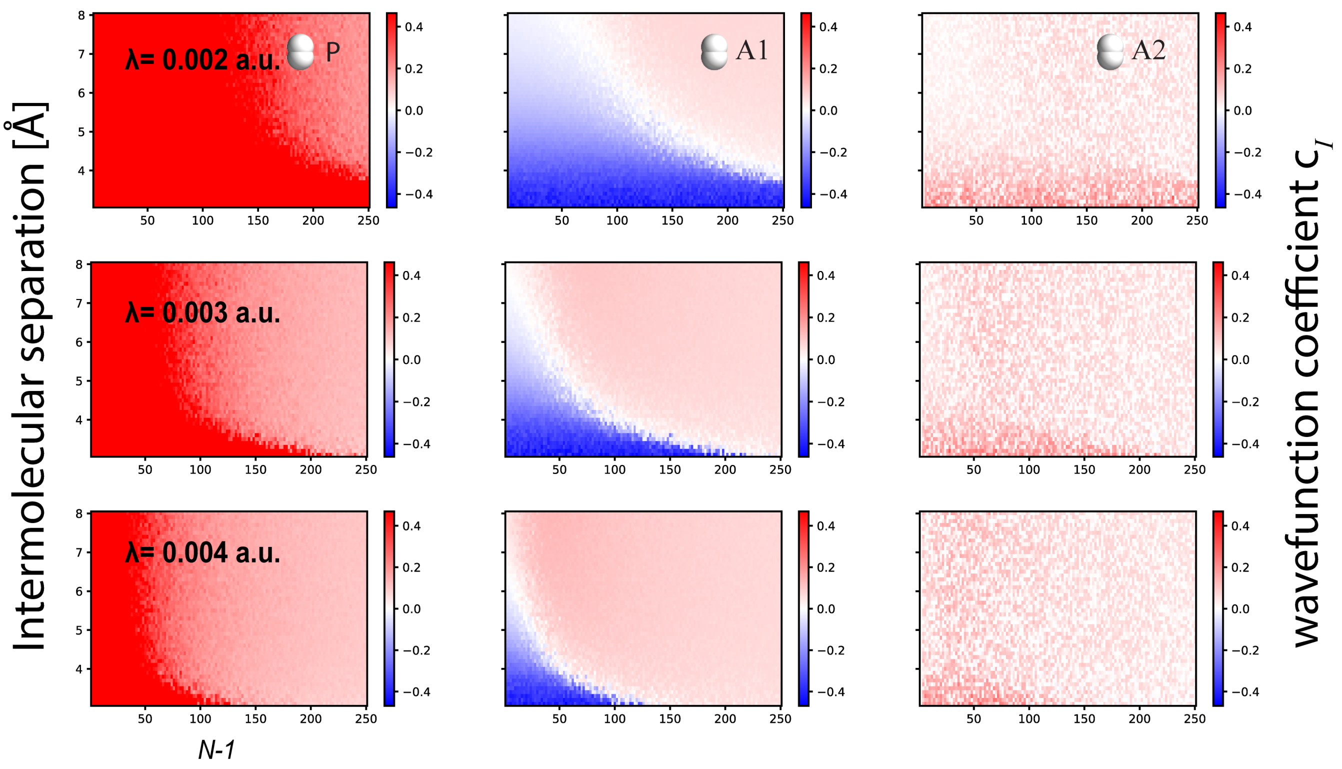

Figure S31: LP wave function coefficients c of P, A1, and A2 (left to right) with coupling strengths $\lambda = 0.002\,$a.u., $0.003\,$a.u. and $0.004\,$a.u. (from the top down to the bottom) of the H-aggregate system with up to 250 bare molecules. Disorder is introduced in the system via a Gaussian energy broadening of the dimers (except for P and A1), with Gaussian width $0.02\,$eV. Moreover, the same dimers are given a random angle with the X axis (while keeping them perpendicular to Y) $\theta_k \in [0, \pi/2]$.

S6. J-aggregate polaritonic excitations

In Figure S32, we show the dressed absorption spectra for J-aggregate (see Figure S1) for 5 Å and 6 Å intermolecular separation. The cavity frequency is tuned to the undressed excitation of the isolated unperturbed dimers. Compared to the H-aggregates (see Figure S16), the middle polariton branch is less intense and loses oscillator strength with shorter intermolecular separations. This reflects the different effects the local arrangement can have on a solute due to the strong directionality of intermolecular forces. As the intermolecular forces become more relevant (shorter distances), the excitations redshift, in agreement with the predictions from the Kasha model (See section). In Figure S33, we plot the LP left and right transition densities for the J-aggregate with an intermolecular distance of 6 Å. Contrary to the H-aggregates, the local transition dipole moments are aligned for every N and show a smaller dependence on the chain length. In Figure S34, we show the corresponding middle polariton. As N increases, the transition dipole moment of A1, initially antialigned with P as in E2, changes sign. As for the H-aggregate, this proves again a local (single-molecule) change stemming from longitudinal and transverse fields. Moreover, the local changes here are more relevant for the middle polaritonic branches (instead of the lower branch as the H-aggregate), which provides a simple proof-of-concept example of how different chemical environments can influence chemical properties due to the directionality of the intermolecular forces.

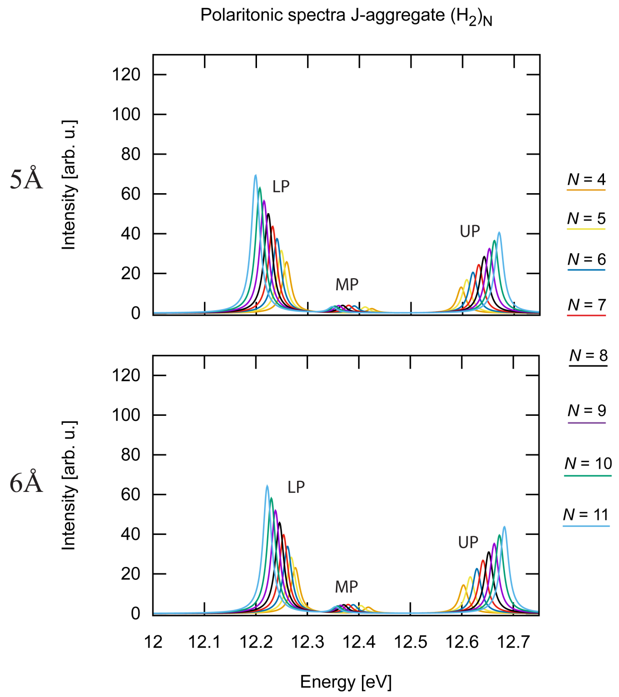

Figure S32: Polaritonic absorption spectra for a J-aggregate $(H_2)_{N\geq4}$ for 5 Å (upper panel) and 6 Å (lower panel) intermolecular separation. From the interaction between the photon field and the H-aggregate electronic excitations E1 and E2, three polaritonic branches, the lower, middle, and upper polariton, emerge.

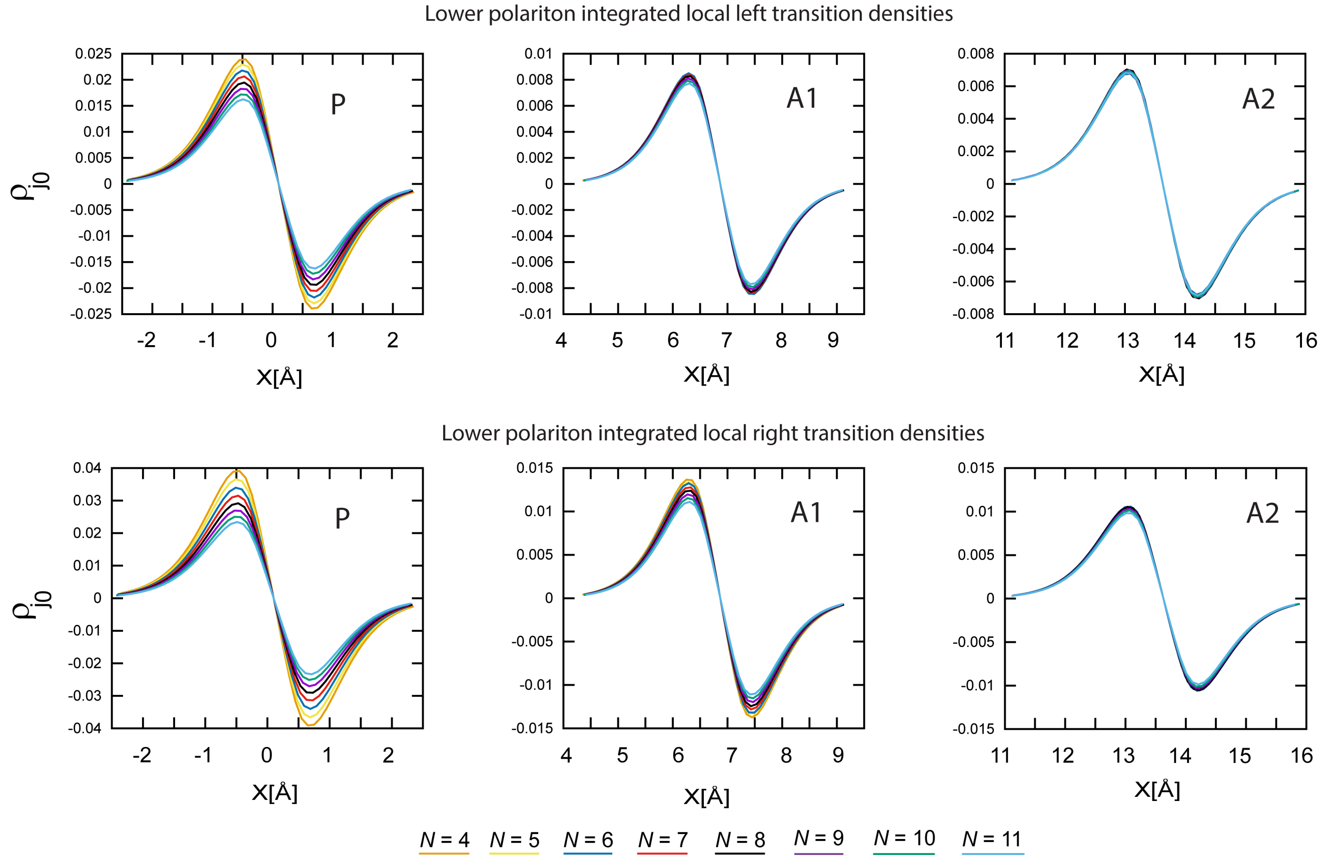

Figure S33: Local left and right transition densities for the lower polariton for an intermolecular separation of 6 Å in a J-aggregate configuration head-tail arrangement, see Figure S1). The local transition dipole moments are all aligned for all N.

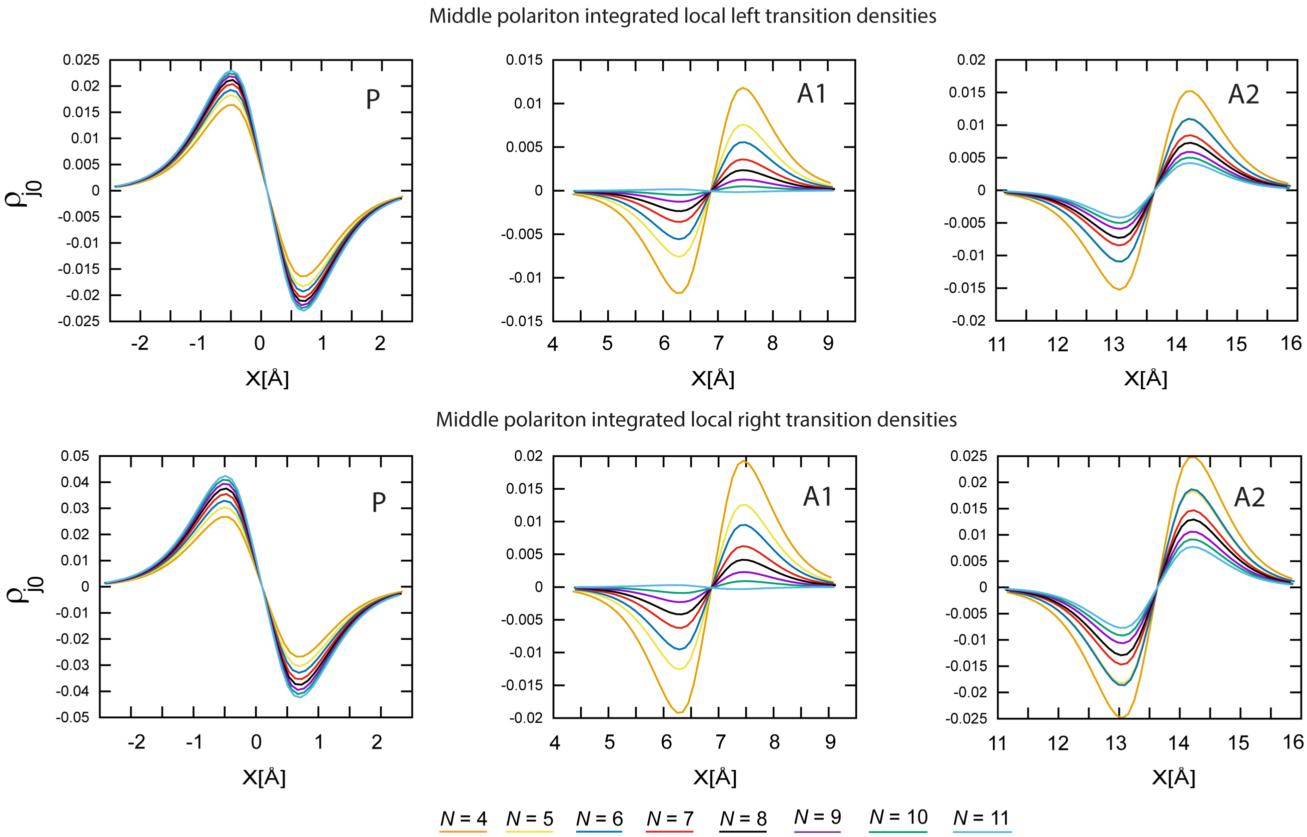

Figure S34: Local left and right transition densities for the middle polariton for an intermolecular separation of 6 Å in a J-aggregate configuration head-tail arrangement, see Figure S1). The local transition dipole moment of A1 changes sign with N.

References

(1) Folkestad, S. D.; Kjønstad, E. F.; Myhre, R. H.; Andersen, J. H.; Balbi, A.; Coriani, S.; Giovannini, T.; Goletto, L.; Haugland, T. S.; Hutcheson, A. et al. e T 1.0: An open source electronic structure program with emphasis on coupled cluster and multilevel methods. *The Journal of Chemical Physics* **2020**, *152*, 184103.

(2) Helgaker, T.; Jørgensen, P.; Olsen, J. *Molecular electronic-structure theory*; John Wiley & Sons, 2014.

(3) Haugland, T. S.; Ronca, E.; Kjønstad, E. F.; Rubio, A.; Koch, H. Coupled cluster theory for molecular polaritons: Changing ground and excited states. *Phys. Rev. X* **2020**, *10*, 041043.

(4) Jaynes, E.; Cummings, F. Comparison of quantum and semiclassical radiation theories with application to the beam maser. *Proceedings of the IEEE* **1963**, *51*, 89–109.

(5) Tavis, M.; Cummings, F. W. Exact solution for an N-molecule—radiation-field Hamiltonian. *Physical Review* **1968**, *170*, 379.

(6) Dicke, R. H. Coherence in spontaneous radiation processes. *Physical review* **1954**, *93*, 99.

(7) Houdré, R.; Stanley, R.; Ilegems, M. Vacuum-field Rabi splitting in the presence of inhomogeneous broadening: Resolution of a homogeneous linewidth in an inhomogeneously broadened system. *Physical Review A* **1996**, *53*, 2711.

(8) Kasha, M.; Rawls, H.; El-Bayoumi, M. A. The exciton model in molecular spectroscopy. *Pure and applied Chemistry* **1965**, *11*, 371–392.

(9) Kasha, M. Energy transfer mechanisms and the molecular exciton model for molecular aggregates. *Radiation research* **1963**, *20*, 55–70.

(10) McRae, E. G.; Kasha, M. Enhancement of phosphorescence ability upon aggregation of dye molecules. *The Journal of Chemical Physics* **1958**, *28*, 721–722.

(11) Kasha, M. Characterization of electronic transitions in complex molecules. *Discussions of the Faraday society* **1950**, *9*, 14–19.

(12) Hestand, N. J.; Spano, F. C. Expanded theory of H-and J-molecular aggregates: the effects of vibronic coupling and intermolecular charge transfer. *Chemical reviews* **2018**, *118*, 7069–7163.

(13) Tomasi, J.; Cammi, R.; Mennucci, B. Medium effects on the properties of chemical systems: An overview of recent formulations in the polarizable continuum model (PCM). *International journal of quantum chemistry* **1999**, *75*, 783–803.

(14) Amovilli, C.; Barone, V.; Cammi, R.; Cancès, E.; Cossi, M.; Mennucci, B.; Pomelli, C. S.; Tomasi, J. Recent advances in the description of solvent effects with the polarizable continuum model. *Advances in Quantum Chemistry* **1998**, *32*, 227–261.

(15) Tomasi, J.; Mennucci, B.; Cammi, R. Quantum mechanical continuum solvation models. *Chemical reviews* **2005**, *105*, 2999–3094.

(16) Czikklely, V.; Forsterling, H.; Kuhn, H. Extended dipole model for aggregates of dye molecules. *Chemical Physics Letters* **1970**, *6*, 207–210.

(17) Gierschner, J.; Park, S. Y. Luminescent distyrylbenzenes: tailoring molecular structure and crystalline morphology. *Journal of Materials Chemistry C* **2013**, *1*, 5818–5832.

(18) Beljonne, D.; Cornil, J.; Silbey, R.; Millié, P.; Brédas, J. Interchain interactions in conjugated materials: The exciton model versus the supermolecular approach. *The Journal of Chemical Physics* **2000**, *112*, 4749–4758.

(19) Wong, C. Y.; Curutchet, C.; Tretiak, S.; Scholes, G. D. Ideal dipole approximation fails to predict electronic coupling and energy transfer between semiconducting single-wall carbon nanotubes. *The Journal of chemical physics* **2009**, *130*.

(20) Krueger, B. P.; Scholes, G. D.; Fleming, G. R. Calculation of couplings and energy-transfer pathways between the pigments of LH2 by the ab initio transition density cube method. *The Journal of Physical Chemistry B* **1998**, *102*, 5378–5386.

(21) Madjet, M. E.-A.; Muöh, F.; Renger, T. Deciphering the influence of short-range electronic couplings on optical properties of molecular dimers: application to "special pairs" in photosynthesis. *The Journal of Physical Chemistry B* **2009**, *113*, 12603–12614.

(22) Haugland, T. S.; Schäfer, C.; Ronca, E.; Rubio, A.; Koch, H. Intermolecular interactions in optical cavities: An ab initio QED study. *J. Chem. Phys.* **2021**, *154*, 094113.

(23) Cao, J.; Pollak, E. Cavity-Induced Quantum Interference and Collective Interactions in van der Waals Systems. *arXiv preprint arXiv:2310.12881* **2023**,

(24) Philbin, J. P.; Haugland, T. S.; Ghosh, T. K.; Ronca, E.; Chen, M.; Narang, P.; Koch, H. Molecular van der Waals fluids in cavity quantum electrodynamics. *arXiv preprint arXiv:2209.07956* **2022**,

(25) Haugland, T. S.; Philbin, J. P.; Ghosh, T. K.; Chen, M.; Koch, H.; Narang, P. Understanding the polaritonic ground state in cavity quantum electrodynamics. *arXiv preprint arXiv:2307.14822* **2023**,
