# Supplementary material for: Collective Strong Coupling Modifies Aggregation and Solvation
Source: J Phys Chem Lett. 2024 Jan 30;15(5):1428–34. doi: 10.1021/acs.jpclett.3c03506 (PMC10860139; doi:10.1021/acs.jpclett.3c03506)
Supplement: Supplementary file 2 — jz3c03506_si_002.pdf [file jz3c03506_si_002.pdf]

jz-2023-03506z.R1

Name: Peer Review Information for "Collective Strong Coupling Modifies Aggregation and Solvation"

First Round of Reviewer Comments

Reviewer: 1

Comments to the Author

Vibrational Strong Coupling (VSC) has emerged as a prominent subject within the realm of physical chemistry, attracting attention due to its substantial influence on various chemical phenomena, including reactions, self-assembly, and crystallization. Despite its significance, the fundamental mechanism underlying the effects of VSC remains incompletely understood. Recently, there has been a growing discussion on the impact of VSC on intermolecular interactions, particularly as the VSC of solvent molecules influences self-assembly and crystallization processes.

In this context, this paper describes theoretical simulations, specifically exploring the effects of VSC on the intermolecular interactions of a chain of N hydrogen molecules. Although a chain of N hydrogen molecules differs from the practicality of solvent molecules employed in experiments, the simplicity of this model enables the separation of intermolecular effects and cavity-induced effects. Furthermore, the simplicity of the model system is justified by considering the computational cost associated with N molecules. The development of theoretical frameworks is imperative to gain a profound understanding of the intricate effects caused by VSC. From my perspective, this theoretical work constitutes an important contribution towards deepening the comprehension of phenomena under the influence of VSC. I recommend the acceptance of this paper, but the following issues need to be addressed.

1)

The authors have defined the value of lambda (light-matter coupling strength) as 0.005 a.u. Does this value closely align with the parameters in experimental systems? I suggest that the authors provide insights into the realism of these parameters. If the specified value significantly deviates from those observed in actual experimental systems, it is essential to comment on the disparity between the chosen value and the values employed in experimental work.

2)

This simulation is based on the model of a chain of H2. The strong coupling of a single cavity mode and N molecules gives N-1 dark states and 2 polaritonic states (upper and lower polaritons). However, a middle polariton emerges in the simulation. Why does the middle polariton emerge through the strong coupling of a single cavity mode and the stretching vibration of N molecules (H2)?

3) The authors posit the impact of VSC on the dynamic polarizability of the first solvation shell. To what extent do the authors anticipate this effect? Although chemical reactions involve substantial energy, particularly in the formation and dissociation of covalent bonds, self-assembly and crystallization can be influenced by minor adjustments in intermolecular interactions. Establishing a correlation between theoretical work and actual experiments is pivotal to enhance the understanding of the VSC effect.

4)

I do not understand the exact meaning of the following sentence in the introduction. Provide clarification to enhance the readability of the manuscript.

>In the following, solvation, aggregation, and nucleation are considered synonymously due to their conceptual similarity.

Reviewer: 2

Comments to the Author

This study explores theoretically how collective strong coupling may affect intermolecular interactions using state-of-the-art QED-CC approach. While many of the experimental studies involve VSC, here the authors use ESC as a model which already reveals many interesting findings about the polarization of the first solvation shell. For instance, the solvent shell will experience much larger effects than the bulk of the solvent molecules and that the target solute (P) does not have to be coupled for the observed effects. Such finding fit well with recent experiments and forms a natural evolution on work in references 62, 65 etc. and in view of how the polaritonic chemistry field is rapidly evolving and expanding, the work will be of interest to many people and therefore merits publication.

Minor comments:

-The reference list is way too long as some of the reference have little to do with the content of the manuscript and should be removed, in particular references 28 thru 36.

- the word impurity will be confusing to some, especially as it appears in the abstract. The best is to define it in the abstract and/or the introduction: a single solute molecule present at the impurity level

Author's Response to Peer Review Comments:

Chalmers University of Technology
Department of Physics

Chalmers, SE-412 96 Gothenburg

Editor
JPCLetter

Christian Schäfer
Gibraltarvallsvägen 7
SE-412 96 Gothenburg

Gothenburg, January 23, 2024

Dear Prof. Editor,

Please find attached the revised manuscript entitled "Collective Strong Coupling Modifies Aggregation and Solvation" by Matteo Castagnola, Tor S. Haugland, Enrico Ronca, Henrik Koch, and Christian Schäfer. A short collection of major additions and a detailed response to the reviewers' comments (relevant comments repeated in italics) is given below. Additionally, we attached a version of the manuscript in which changes are highlighted.

The manuscript has received improvements in the form of:

- a better clarification for the usage of 'impurity' in the context of our work and the relation of this effect to solvation/nucleation/aggregation effects
- an extended discussion for the relevance of the light-matter coupling strength
- a brief discussion on the expected changes for different molecular systems
- various stylistic adjustments to adhere to the guideline set out by JPCLetter

**Reviewer 1:**

*Vibrational Strong Coupling (VSC) has emerged as a prominent subject within the realm of physical chemistry, attracting attention due to its substantial influence on various chemical phenomena, including reactions, self-assembly, and crystallization. Despite its significance, the fundamental mechanism underlying the effects of VSC remains incompletely understood. Recently, there has been a growing discussion on the impact of VSC on intermolecular interactions, particularly as the VSC of solvent molecules influences self-assembly and crystallization processes. In this context, this paper describes theoretical simulations, specifically exploring the effects of VSC on the intermolecular interactions of a chain of N hydrogen molecules. Although a chain of N hydrogen molecules differs from the practicality of solvent molecules employed in experiments, the simplicity of this model enables the separation of intermolecular effects and cavity-induced effects. Furthermore, the simplicity of the model system is justified by considering the computational cost associated with N molecules. The development of theoretical frameworks is imperative to gain a profound understanding of the intricate*

*effects caused by VSC. From my perspective, this theoretical work constitutes an important contribution towards deepening the comprehension of phenomena under the influence of VSC. I recommend the acceptance of this paper, but the following issues need to be addressed.*

We thank the reviewer for the very positive report that led to clear improvements in the revised manuscript, and we are grateful that the reviewer shares our vision for the significance of this work.

*1) The authors have defined the value of lambda (light-matter coupling strength) as 0.005 a.u. Does this value closely align with the parameters in experimental systems? I suggest that the authors provide insights into the realism of these parameters. If the specified value significantly deviates from those observed in actual experimental systems, it is essential to comment on the disparity between the chosen value and the values employed in experimental work.*

Figures 2, 4, and 5 in the main manuscript are obtained with a fundamental coupling strength of 0.005 a.u., which corresponds to values achievable with plasmonic cavities, but we would like to draw the reviewers attention to figure 3 and the SI, where we illustrate results for dynamically changing (and much smaller) coupling strength. It is important to consider, that the "screening" or "sign-flip" effect that the solute exerts on the first solvation shell depends foremost on the collective coupling strength. As illustrated in Figure 3 (a), where we reduce the fundamental coupling for an increasing number of solvent molecules, the qualitative behavior also exists at much smaller coupling values. Smaller coupling values shift the "complete screening" (white domain in Figure 3 a) towards larger intermolecular separations, which could be interpreted as changes in the tail of dispersive interactions. In other words, a central conclusion of our work is that changes in intermolecular interaction can originate from *collective* strong coupling, suggesting that the effect remains relevant at much smaller coupling values. Note that molecules with strong optical transitions will allow to observe comparable effects at much smaller fundamental coupling strength $\lambda$ since the effective coupling $g \sim \sqrt{N}\mu\lambda$ can be much larger for molecules with strong optical transitions (large $\mu$). Furthermore, the intermolecular interactions, especially those between dipolar or fractionally charged molecules, are substantially more long-ranged than in our hydrogen system. We now emphasize those aspects stronger in the manuscript and added a brief discussion to the conclusion.

*2) This simulation is based on the model of a chain of H2. The strong coupling of a single cavity mode and N molecules gives N-1 dark states and 2 polaritonic states (upper and lower polaritons). However, a middle polariton emerges in the simulation. Why does the middle polariton emerge through the strong coupling of a single cavity mode and the stretching vibration of N molecules (H2)?*

The single optical cavity mode couples strongly to the electronic transition of N-1 identical molecules (the solvent or aggregate molecules, labelled as A1 etc) and a single perturbed molecule (the solute, labelled as P throughout the manuscript). This leads to two excitations

in the matter spectrum (see Fig. 1, E1 for the solute and E2 for the solvent molecules). The cavity can now mix those states which results in N-2 dark states and 3 polaritonic states (LP, MP and UP).

*3) The authors posit the impact of VSC on the dynamic polarizability of the first solvation shell. To what extent do the authors anticipate this effect? Although chemical reactions involve substantial energy, particularly in the formation and dissociation of covalent bonds, self-assembly and crystallization can be influenced by minor adjustments in intermolecular interactions. Establishing a correlation between theoretical work and actual experiments is pivotal to enhance the understanding of the VSC effect.*

We agree entirely with the reviewers perspective on the significance of this question and a faithful connection between theory and experiments. Based on our current investigations, we can conclude that the dynamic polarization that participates in the collective polaritonic states changes for the 1st solvation shell due to the interplay with intermolecular interactions. Such a change suggests adjustments in the intermolecular forces, but to which extend the effect impacts experiments in VSC remains speculative. However, that intermolecular interactions play a considerable role to obtain a finite and realistic refractive index in atomic ensembles has been observed in the past[1]. Furthermore, compensating effects between self-polarization contributions and Coulomb interactions are important arguments in the derivation of quantum optical models[2]. The precise interplay for molecular systems, its connection to polaritonic features, and its impact on VSC remains to be fully understood but our manuscript adds an important new facet to this discussion. We are confident that the here discussed effect will play a role in VSC experiments.

*4) I do not understand the exact meaning of the following sentence in the introduction. Provide clarification to enhance the readability of the manuscript.*
*> In the following, solvation, aggregation, and nucleation are considered synonymously due to their conceptual similarity.*

The observed effect originates from the interplay of local longitudinal interactions (dispersive, dipole-dipole, ...) and the collective transversal interaction mediated by the polaritonic states. While solvation, aggregation, and nucleation are clearly different processes, they all describe the emergence of structure in molecular ensembles via intermolecular forces. Our discussion can, therefore, be transferred to all three realizations. Nonetheless, we agree with the reviewer that the sentence was not sufficiently clear. The revised sentence reflects now correctly the intended meaning: ~~In the following, solvation, aggregation, and nucleation are considered synonymously due to their conceptual similarity.~~ Our simple model - a stretched molecule

[1] Francesco Andreoli, Michael J. Gullans, Alexander A. High, Antoine Browaeys, and Darrick E. Chang, Phys. Rev. X 11, 011026, 2021.

[2] The community has seen an extensive discussion on the need for self-polarization contributions, e.g., `https://pubs.acs.org/action/showCitFormats?doi=10.1021/acsphotonics.9b01649&ref=pdf`. Nonetheless, a seamless connection from microscopic theory to optical features in the disordered ensemble is yet to be established.

embedded in a perfectly ordered environment - can represent a solute in a solvent, a nucleation center, or an impurity in an aggregate. Indeed, these physical realizations are associated with intermolecular interactions with a surrounding chemical environment. Therefore, solvation, aggregation, and nucleation are used interchangeably in the following due to their conceptual similarity.

**Reviewer 2:**

*This study explores theoretically how collective strong coupling may affect intermolecular interactions using state-of-the-art QED-CC approach. While many of the experimental studies involve VSC, here the authors use ESC as a model which already reveals many interesting findings about the polarization of the first solvation shell. For instance, the solvent shell will experience much larger effects than the bulk of the solvent molecules and that the target solute (P) does not have to be coupled for the observed effects. Such finding fit well with recent experiments and forms a natural evolution on work in references 62, 65 etc. and in view of how the polaritonic chemistry field is rapidly evolving and expanding, the work will be of interest to many people and therefore merits publication.*

We thank the reviewer for the positive report and we are grateful that the reviewer shares our vision.

*Minor comments: -The reference list is way too long as some of the reference have little to do with the content of the manuscript and should be removed, in particular references 28 thru 36.*

We reduced the number of references according to the reviewers suggestions.

*- the word impurity will be confusing to some, especially as it appears in the abstract. The best is to define it in the abstract and/or the introduction: a single solute molecule present at the impurity level*

We thank the reviewer for pointing out this potential confusion. The revised version includes now such a clarifying statement as suggested by the reviewer.

We thank the reviewers again for their very positive and constructive comments. We are confident that the revised manuscript is a significant improvement over the original version and provides a valuable addition to the scientific literature.

Yours sincerely,

Christian Schäfer,
on behalf of all authors.
